# Choosing an appropriate probiotic product for your patient: An evidence-based practical guide

**DOI:** 10.1371/journal.pone.0209205

**Published:** 2018-12-26

**Authors:** Jason C. Sniffen, Lynne V. McFarland, Charlesnika T. Evans, Ellie J. C. Goldstein

**Affiliations:** 1 Department of Internal Medicine, Infectious Disease Section, Florida Hospital Orlando, Orlando, FL, United States of America; 2 Department of Medicinal Chemistry, School of Pharmacy, University of Washington Medical Center, Seattle, Washington United States of America; 3 Department of Preventive Medicine and Center for Healthcare Studies, Feinberg School of Medicine, Northwestern University, Chicago, IL, United States of America; 4 Department of Veterans Affairs (VA), Center of Innovation of Complex Chronic Healthcare (CINCCH), Edward Hines, Jr. VA Hospital, Hines, IL, United States of America; 5 RM Alden Research Laboratory and David Geffen School of Medicine at UCLA, Los Angeles, CA, United States of America; Universidade Federal do Rio de Janeiro, BRAZIL

## Abstract

**Introduction:**

Clinicians and patients face a daunting task when choosing the most appropriate probiotic for their specific needs. Available preparations encompass a diverse and continuously expanding product base, with most available products lacking evidence-based trials that support their use. Even when evidence exists, not all probiotic products are equally effective for all disease prevention or treatment indications. At this point in time, drug regulatory agencies offer limited assistance with regard to guidance and oversight in most countries, including the U.S.

**Methods:**

We reviewed the current medical literature and sources on the internet to survey the types of available probiotic products and to determine which probiotics had evidence-based efficacy data. Standard medical databases from inception to June 2018 were searched and discussions with experts in the field were conducted. We graded the strength of the evidence for probiotics having multiple, randomized controlled trials and developed a guide for the practical selection of current probiotic products for specific uses.

**Results:**

We found the efficacy of probiotic products is both strain-specific and disease-specific. Important factors involved in choosing the appropriate probiotic include matching the strain(s) with the targeted disease or condition, type of formulation, dose used and the source (manufacturing quality control and shelf-life). While we found many probiotic products lacked confirmatory trials, we found sufficient evidence for 22 different types of probiotics from 249 trials to be included. For example, several types of probiotics had strong evidence for the prevention of antibiotic-associated diarrhea [*Saccharomyces boulardii* I-745, a three-strain mixture (*Lactobacillus acidophilus* CL1285, *L*. *casei* Lbc80r, *L*. *rhamnosus* CLR2) and *L*. *casei* DN114001]. Strong evidence was also found for four types of probiotics for the prevention of a variety of other diseases/conditions (enteral-feed associated diarrhea, travellers’ diarrhea, necrotizing enterocolits and side-effects associated with *H*. *pylori* treatments. The evidence was most robust for the treatment of pediatric acute diarrhea based on 59 trials (7 types of probiotics have strong efficacy), while an eight-strain multi-strain mixture showed strong efficacy for inflammatory bowel disease and two types of probiotics had strong efficacy for irritable bowel disease. Of the 22 types of probiotics reviewed, 15 (68%) had strong-moderate evidence for efficacy for at least one type of disease.

**Conclusion:**

The choice of an appropriate probiotic is multi-factored, based on the mode and type of disease indication and the specific efficacy of probiotic strain(s), as well as product quality and formulation.

**Trial registration:**

This review was registered with PROSPERO: CRD42018103979.

## Introduction

As the use and diversity of probiotic products grows, so does the confusion on how to choose the best probiotic for your patient. Because probiotics may be available as dietary supplements or over-the-counter products in many countries, national drug regulatory agencies cannot provide the same level of clinical guidance that they do for prescription medications. The responsibility falls on medical providers and the public, who are faced with a plethora of probiotic products and health claims.

The use of probiotics has become increasing popular across the world and surveys report probiotic use ranges from 5% in one general public survey in the United States to 25% in another survey done in New Zealand [1,2]. In one survey of patients at a tertiary medical center in California, as many as 55% of the patients reported probiotic use in the past three months [3]. Probiotics are also being used more frequently in hospitalized patients to prevent infections (2.6% of all hospital visits reported probiotic use) [4]. The popularity of probiotic products has increased exponentially over the recent years and now has reached 35 Billion dollars in sales in the U.S. [5].

Healthcare providers and laypeople are often confused by the wide range of probiotic products available and the high-pitched marketing claims found on many websites [3,6]. Probiotics are marketed for a variety of diseases, ranging from preventing diarrheal diseases to controlling chronic diseases and treating obesity [7,8]. But the number of publications and the availability of on-line websites has led to a knowledge-overload that is difficult to navigate. There has been a seven-fold increase in the number of published articles on probiotics, reaching over 1,400 in 2014 [7]. One survey found only 12% of probiotic users relied on their physician to recommend a probiotic for them [3]. In a survey of 632 physicians, although 61% recommended probiotics, 40% left the choice of the probiotic product up to their patient [6]. As the internet has increased access to information, the public are increasingly relying on recommendations found on different websites, but there is no regulated validation on these sources and the accuracy varies greatly from one website to another [9–11]. A group of probiotic experts also reported rampant confusion on the different types of probiotic products and label information commonly did not represent the strains or quantity found in the product [11].

Choosing an appropriate probiotic is challenging, as a variety of factors are involved: the strain-specific and disease-specific efficacy probiotic products, differences in the mechanisms-of-action for different probiotic strains, differences in manufacturing processes and quality control of the products and differences in international regulatory requirements. International guidelines from infectious disease or pediatric disease organizations do not always agree with which probiotics should be used for each type of disease condition [12–15].

This paper reviews the current literature for a wide range of disease indications for studies with a well-defined probiotic intervention for either pediatric or adult patients to provide practical recommendations on how healthcare providers and the public can choose the most appropriate probiotic product(s) for their needs.

## Materials and methods

### Literature search

A literature search was conducted using PRISMA (Preferred Reporting Items for Systematic reviews and Meta-Analysis) statement guidelines [16] from date of database inception to June 3, 2018 (S1 PRISMA Checklist). We undertook systematic searches of PubMed (1960–2018), EMBASE (1974–2018), Cochrane Database of Systematic Reviews (1990–2018), ISI Web of Science (2000–2018) and two on-line clinical trial registries: Cochrane Central Register of Controlled trials (http://www.cochrane.org) and National Institutes of Health (http://www.clinicaltrials.gov). We updated previous literature searches from meta-analyses on probiotics for various diseases using the same protocol previously described [17–19]. This review was registered with PROSPERO: CRD42018103979. We used bibliographies of all relevant studies to do a recursive search. Additionally, we conducted an extensive grey literature search including abstracts from annual infectious disease and gastroenterology meetings, probiotic product websites, experts in the field and communication with published authors on probiotics. Search terms included: probiotics, randomized clinical trials (RCTs), antibiotic associated diarrhea (AAD), *Clostridium difficile* infection (CDI), inflammatory bowel disease (IBD), irritable bowel syndrome (IBS) and *H*. *pylori* (example of search strategy provided in S1 Text). We also explored internet websites of probiotic products to determine the validity of their health claims and types of supporting evidence. In addition to examine what other factors are important for probiotic choice, we also reviewed systematic reviews, meta-analyses, pre-clinical studies, studies on formulation, safety, regulations and quality control.

### Study eligibility for efficacy

Inclusion criteria for articles assessing probiotic efficacy included: randomized controlled (placebo or open control) trials (RCTs) of a probiotic intervention (single strain or multi-strain mixture), in adults or children, reported duration of intervention and/or follow-up, for either prevention or treatment of disease and peer-reviewed publication (language unrestricted). We included RCTs with primary outcomes of either prevention or treatment of various diseases and, if present, secondary outcomes and additional treatment arms in this review. As the efficacy of probiotics is both strain-specific and disease-specific [20], each type of probiotic was required to have at least two RCTs for each disease indication being considered. The probiotic intervention was required to be well defined by strain or strains if a mixture of species were used and the daily dose. Exclusion criteria for efficacy assessments included: reviews, kinetic or safety studies, non-randomized trials, duplicate reports, and trials with insufficient descriptions of the type of probiotic. Probiotic products with less than two RCTs per disease indication were excluded. Disease indications with less than two RCTs per probiotic type were also excluded. Pre-clinical studies of mechanisms of action or safety studies were also excluded from the efficacy assessments.

### Data extraction

Initial screening of studies was done independently by two researchers following the protocols used in prior meta-analyses of the co-authors [17–19]. Inclusion of studies into the review was determined with agreement of three authors (LVM, CTE, EJCG). The data extracted from each study used a standard data extraction form for PICOS data: (1) patient population (adult/pediatric, age, gender), (2) intervention (type of probiotic and controls used, daily doses, duration and follow-up), (3) comparisons (type of control group either placebo or open, unblinded), (4) outcomes (for prevention trials, the incidence of disease or for treatment trials, the reduction of symptom or scores or recurrence of disease) and (5) study design (all were randomized, controlled trials, either double blinded or open). Risk of bias was not determined in this review. Necessary data (probiotic strain, dose, outcome measures, etc.) not reported in published articles was collected by contacting authors whenever possible.

### Grading of efficacy evidence

Efficacy was based on documenting at least two RCTs that found a significant reduction of either the incidence of disease (prevention trials) or a reduction in clinical symptoms (treatment trials). The strength of the evidence was graded based on standard methodology to assess intervention trials [21, 22]. For this assessment, the total number of RCTs with significant efficacy findings were summed and the total number of RCTs with non-significant (p>0.05) efficacy was subtracted. The *net* number of RCTs with significant (or non-significant findings) determined the strength of the efficacy evidence. The grading of the strength was done for each sub-group of probiotic strain or mixture by type of disease. For our paper, *strong* strength of evidence was defined as finding ≥ 2 more RCTs with significant (p<0.05) efficacy findings compared to the total number of RCTs with non-significant findings; *moderate* strength was defined as finding a total of one more significant RCT than the total number of non-significant RCTs and *weak* strength was defined as finding either an equal number or fewer total RCTs with significant efficacy compared to the total RCTs non-significant efficacy findings. A finding of *not effective* was defined as finding a *net* of ≥2 studies with non-significant efficacy compared to the number of studies with significant efficacy outcomes.

### Other factors impacting probiotic choice

We also re-examined the literature for studies examining various other factors that might influence the efficacy of a specific probiotic. Inclusion criteria included: studies on dose ranging, types of formulations, manufacturing processes, product stability, quality control, mis-labeling issues, safety studies and regulatory issues.

## Results

**Fig 1:**
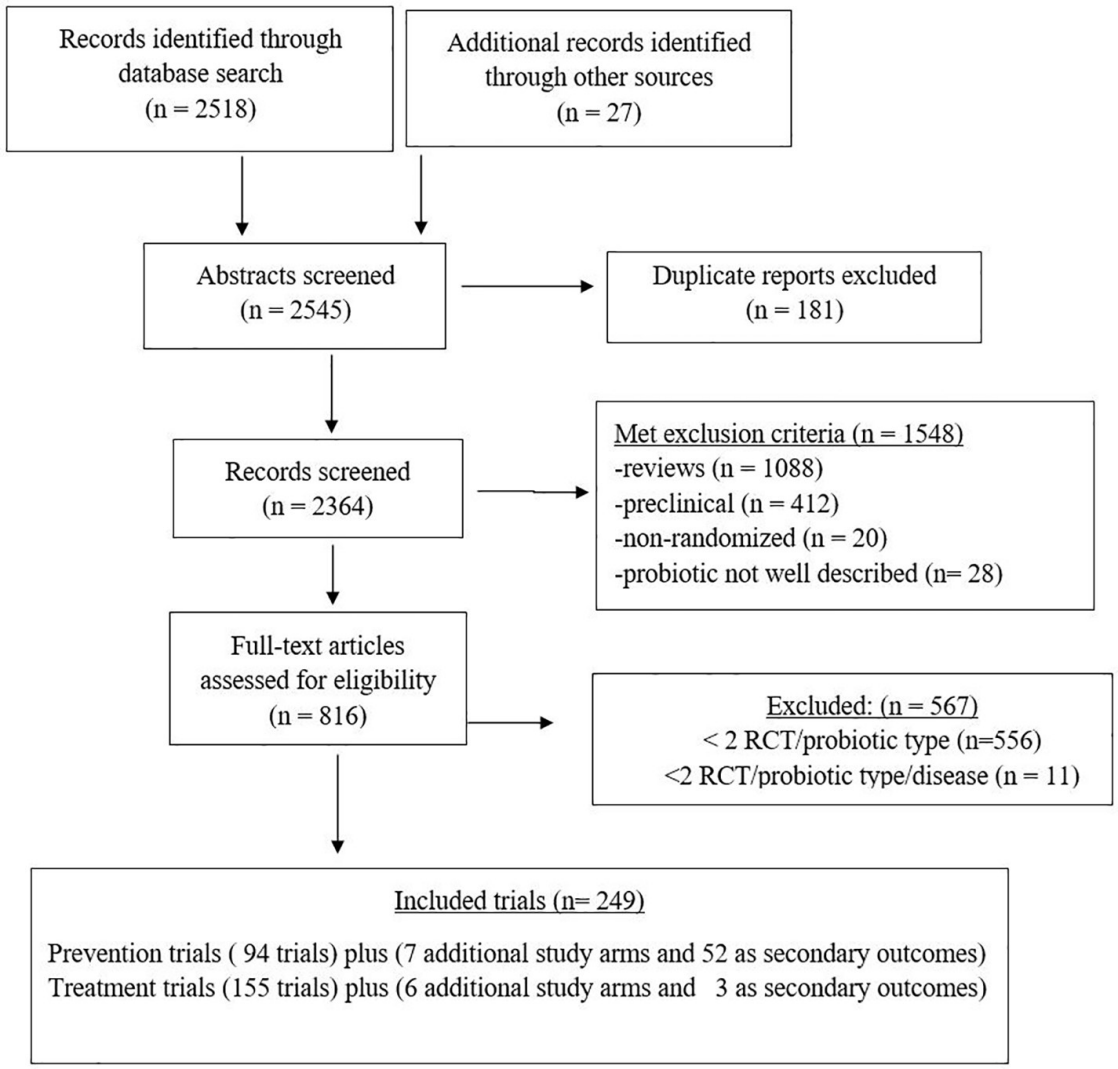
PRISMA flow diagram of evaluated studies for randomized controlled trials for efficacy of specific probiotics for the prevention or treatment of various diseases, searched from inception of databases to June 2018. Outcomes were extracted from randomized controlled trials (RCT) with a primary outcome and may have included additional secondary outcomes and/or additional treatment arms per trial.

Our search found 2545 articles relating to probiotics and clinical disease, and after excluding duplicate reports (n = 181), reviews (n = 1088), pre-clinical studies (n = 412), non-randomized clinical trials (n = 20) and studies with incomplete descriptions of the probiotic (n = 28), 816 full articles were reviewed for efficacy assessments (Fig 1). Those trials having fewer than two RCTs per type of probiotic (556 RCTs) or probiotic types with fewer than two RCTs per type of disease (11 RCTs) were then excluded. As a consequence, we excluded eight probiotics [*Bacillus coagulans*, *L*. *casei* Shirota, *L*. *casei rhamnosus* Lcr35, *L*. *johnsonii* La1, and three mixtures (Lactinex, Protectis, Linex)] and ten different disease indications (acne, asthma, arthritis, cholera, cystic fibrosis, dental infections, general gastrointestinal health, hypertension, pancreatitis and weight control) with insufficient numbers of RCTs from our review.

We included a total of 249 RCTs in this review either for the prevention or treatment of various diseases. In 94 RCTs, the main aim was for the prevention of one of 11 types of disease indications. As many RCTs also included secondary outcomes and additional treatment arms (with a different dose or probiotic type), we also included those outcomes. To assess probiotics for the prevention of these 11 diseases, we included primary outcome data from the 94 preventive trials, data from 7 additional study arms and data from 52 other trials with prevention as a secondary outcome, as shown in S1 References and S1 Dataset. For example, efficacy data for the prevention of antibiotic-associated diarrhea (AAD) was gathered from 45 study arms where AAD was the primary outcome and 16 other trials AAD was reported as a secondary outcome (15 *H*. *pylori* treatment trials and one *C*. *difficile* trial).

In contrast, most of the outcomes for the prevention of CDI (21/23) were extracted as secondary outcomes in trials for the prevention of AAD or respiratory tract infections and only two trials had CDI as their primary outcome. Most of the study outcomes for the prevention of adverse reactions arising from *H*. *pylori* therapy (12/14 trials) were obtained as secondary outcomes from *H*. *pylori* treatment trials.

In 155 RCTs, the main aim was for the treatment of one of eight types of diseases. A total of 164 outcomes were assessed (primary outcomes from 155 treatment trials, three as secondary outcomes and six additional treatment arms (S1 Dataset). Trials with more than one treatment arm were found. Two trials for the eradication of *H*. *pylori* tested an additional probiotic in a separate treatment arm and one study for the treatment of acute pediatric diarrhea tested three additional probiotic types (S1 Dataset).

### Diversity of available probiotic products

The first challenge in choosing an appropriate probiotic is the diversity of products available from which to choose and the lack of guidance on which products may be more effective. Probiotics are defined as ‘live microorganisms that, when administered in adequate amounts, confer a health benefit on the host” [23]. However, the burden of proof for health benefits varies by country. We found a variety of types of probiotic products available on the market as prescription products, as over-the-counter products, or as dietary supplements. In the U.S.A., probiotics currently are available as ‘dietary supplements’, which are only allowed generalized health claims that relate to host structure or function. Twenty-five types of probiotic products commonly found as over-the-counter dietary supplements or on-line are shown in Table 1. Unfortunately, the health claims on the labels of dietary supplements (shown in Table 1) often do not provide practical clinical guidance for healthcare providers nor for the general public. Several probiotic products are being developed as potential drugs under the F.D.A. regulated pathway, but none have been approved currently as prescription products. Probiotics have a long history of use in Europe and Asia, but in 2013, the use of ‘probiotic’ was disallowed on the label due to the disparity of evidence for various claims for health benefits [24]. Currently, regulations relating to probiotics are in flux and differ dramatically from country to country.

**Table 1:** Examples of 25 probiotic products available on the commercial market.

Probiotic description (genus, species and strain)	Formulation	Examples of brand names (Manufacturer, Country)	Examples of claims on label
**Single strain probiotics**			
*Bacillus coagulans* nr	capsules	ProbioSlim (ProbioSlim, Canada, USA)	“Promotes healthy digestion, helps you lose weight”
*B*. *lactis (animalis)* DN-173010 (CNCM I-2494)	yogurt	Activia (Danone, France)	“Supports digestive health”
*B*. *animalis lactis* Bb-12 (CNCM I-3446)	capsules, powder in sticks, fermented milk	BB-12 (Chr Hansen, USA)	“Supports bowel function, reduces crying in infants”
*B*. *infantis* 35624	drink, capsules	Align (Proctor & Gamble, USA)	“Maintains digestive balance”
*Clostridium butyricum* 588	tablets, drink	MIYA-BM (Miyarisan Pharm, Japan)	“Intestinal health”
*Enterococcus faecium* SF 68	powder, sachets	Bioflorin (Sanofi, Germany)	“To treat diarrhea”
*Escherichia coli* Nissle 1917	capsules	Mutaflor (Pharma-Zentiale, Germany)	“For chronic constipation or ulcerative colitis”
*L*. *acidophilus* LB	sachet, capsules	Lacteol (Mirren, South Africa)	“Preserves intestinal peristalsis”
*L*. *casei* Shirota	fermented milk	Yakult (Yakult, USA)	“Balance your digestive system”
*L*. *casei* DN-114001 (CNCM I-1518)	fermented drink, yogurt	Actimel (in Europe) or DanActive (in USA) (Danone, France)	“Supports normal function of immune system”
*L*. *casei rhamnosus* Lcr35	vaginal capsules	Gynophilus (Biose, France)	“Helps to recover vaginal flora”
*L*. *johnsonii* La1	milk	Bioamicus johnsonii (BioAmicus Probiotics, Canada)	“Assists child’s immune system from allergies, eczema, asthma”
*L*. *plantarum* 299v (DSM9843)	fermented oat gruel in fruit drink, capsules	ProViva (Probi AB, Sweden)	“Relieve symptoms of IBS”
*L*. *reuteri* DSM 17938	capsules, yogurt	Protectis (BioGaia, Sweden)	“For digestive comfort”
*L rhamnosus* GG (ATCC 53013)	yogurt, capsules	Culturelle (i-Health, Inc, USA)	“Improves digestive health” “Boosts immune system”
*S*. *boulardii* CNCM I-745 (ATCC 74012)	capsules, sachets	Florastor (Biocodex, France)	“Strengthens digestive balance” “Boosts immune response”
**Mixtures of probiotic strains**			
*L*. *acidophilus* CL1285 *+L*. *casei* Lbc80r + *L*. *rhamnosus* CLR2	fermented drink, capsules	Bio K+ (BioK+ Intl, Canada)	“Maintains healthy intestinal flora”
*L*. *helveticus* R52 (CNCM I-1722) + *L*. *rhamnosus* R11 (CNCM I-1720)	capsules, sachets	Lacidofil (Institut Rosell, Canada)	“Maintains normal healthy intestinal microflora”
*L*. *helveticus (bulgaricus)* 33409 *+ L*. *gasseri* 4962	tablets or sachets	Lactinex (Becton, Dickinson and Comp., USA)	“Replaces intestinal flora”
*L*. *reuteri* DSM17938 *+ L*. *reuteri* PTA5289	lozenges, powder, capsules	Protectis (BioGaia, Sweden)	“Helps restore balance in digestive tract”
*L*. *acidophilus* La5 *+ B*. *lactis* Bb12	yogurt	AB Yogurt (President Enterprise Company, Taiwan)	“Improves normal flora”
*L*. *acidophilus* nr *+B*. *infantis* nr	capsules	Infloran Berna (Berna, Switzerland)	“Helps restore balance of intestinal bacteria” “Used for diarrhea, vaginal and urinary tract infections”
*L*. *acidophilus gasseri +* *B*. *infantis* nr	capsules	Linex (Sandoz, Bulgaria)	“Normalizes normal flora”
*Bacillus clausii* (4 strains, O/C, N/R84, T84, Sin8)	capsules, spores in vial	Enterogermina (Sanofi-Aventis, France)	“Management of pediatric diarrhea, for intestinal flora balance disturbed by diarrhea, poisoning and antibiotics”
*Bifido*.*longum* BL03, *Bifido*. *infantis* subsp. *lactis* BI04, *Bifido*. *breve* BB02, *L*. *acidophilus* BA05, *L*. *plantarum* BP06, *L*. *paracasei* BP07, *L*. *helveticus* BD08, *Strept thermophiles* BT01	sachets	VSL#3 (VSL Pharm, Inc., Italy)	“Management of ulcerative colitis, ileal pouch and IBS”

**Abbreviations**: ATCC, American Type Culture Collection; B., *Bifidobacterium*; CNCM, Collection Nationale de Cultures de Microorganisms; DSM, Deutsche Sammlung von Mikroorganismen; IBS, irritable bowel syndrome; L., *Lactobacillus*; nr, strain not reported; S., *Saccharomyces boulardii*; St., *Streptococcus*.

Only one health claim for probiotics has been approved in the European Union, that is for yoghurt to improve lactose tolerance [25]. Examples of commercial health claims on European and Asian products may not conform to specific regulations, but they may provide more useful guidance for specific uses from evidence-based efficacy trials. For example, probiotic products available in Europe and Asia state their products may ‘treat *H*. *pylori* infections’ or ‘treat diarrhea’. In contrast, dietary supplements found in U.S.A. are allowed only structure/function health claims, such as ‘maintains intestinal balance’ or ‘promotes immune health’. As the regulations differ by country and are in flux, for this paper we will refer to ‘dietary supplements’ or ‘novel foods with health benefits’ as ‘probiotics’ only if they are supported by randomized, controlled trials showing a significant efficacy for preventing or treating a disease or condition being studied.

### Factors influencing probiotic efficacy

**Fig 2:**
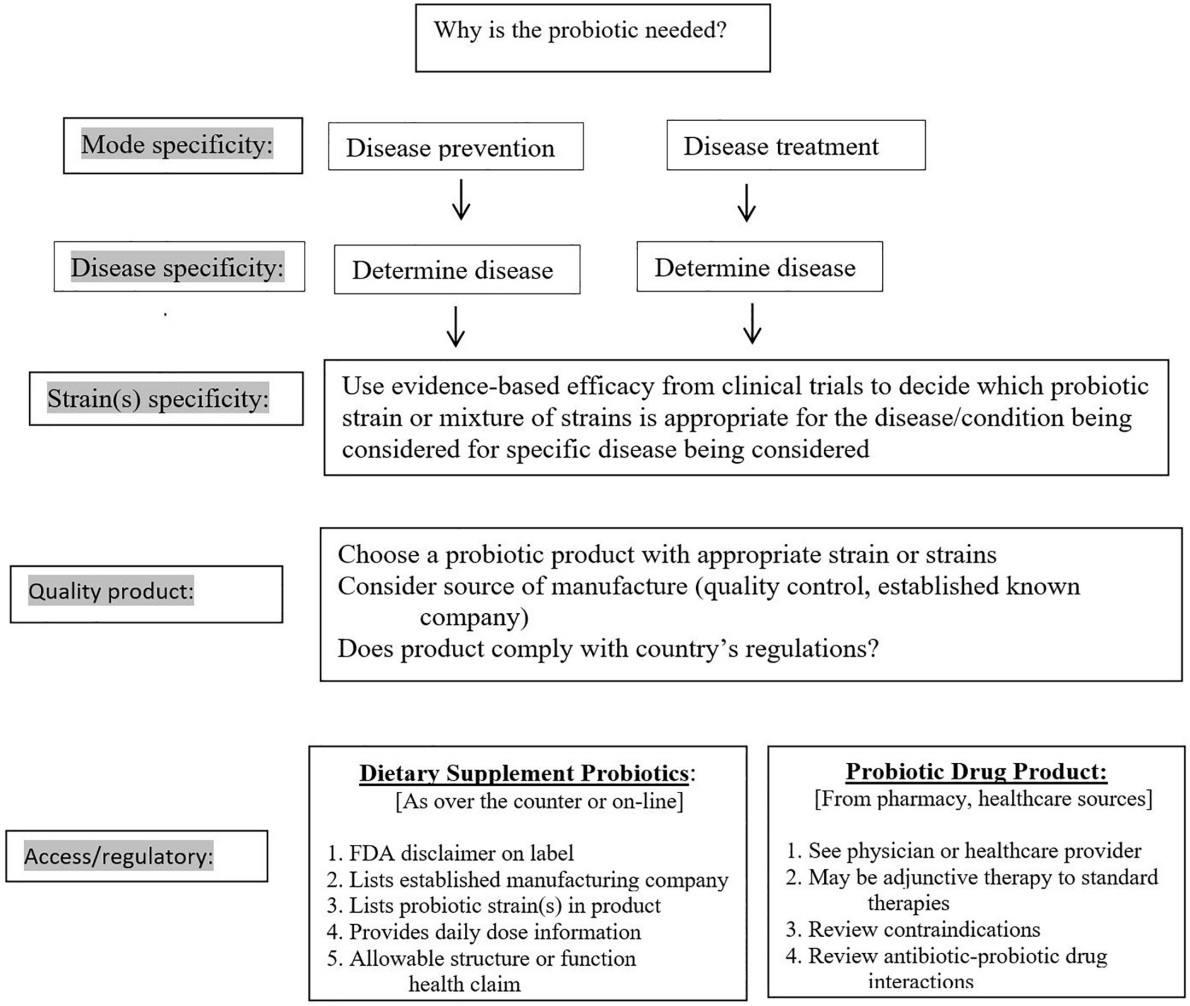
Clinical decision algorithm for choosing an appropriate probiotic product.

Our review of the literature found efficacy of probiotics may vary according to several factors: mode of therapy, disease indication and strain or strains of probiotic. The choice of an appropriate probiotic is dependent upon all three of these factors, as shown in Fig 2. The first important factor is the mode of therapy, in that, while a specific probiotic may be effective for the treatment of a disease, it may not show the same degree of efficacy as prevention of the same disease. An example of this mode specificity is seen with acute pediatric diarrhea. One strain of probiotic, *L*. *rhamnosus* GG was found to be ineffective for the prevention of acute pediatric diarrhea in one meta-analysis pooling the results of five RCTs (RR = 0.60, 95% confidence interval (CI) 0.30–1.09) [26], but was significantly effective for the treatment of acute pediatric diarrhea [27].

The second factor is disease specificity, in that one probiotic may be effective for one disease, yet not in another. An example of disease specificity is shown by *L*. *rhamnosus* GG, which was found in one meta-analysis to be significantly effective for the prevention of pediatric antibiotic-associated diarrhea (AAD) (RR = 0.44, 95 C.I. 0.21, 0.96), but not effective for other diseases including Crohn’s disease, *Clostridium difficile* infections (CDI), nosocomial infections or traveler’s diarrhea [20]. The efficacy of probiotics is also strain specific, decades of research have shown that only specific strains of bacteria or fungus have health benefits [7, 20, 28, 29]. Shifting taxonomy of the species and strains are also challenging and add to the confusion when evaluating the literature [30–32]. As the efficacy of probiotics is also disease-specific, we will examine the strain-specificity within several types of diseases or conditions. When the 22 different types of probiotics with ≥2 RCTs per disease were reviewed (14 single strain probiotics and 8 multi-strain mixtures), both mode and disease specificity were found for the specific probiotic formulations. Our practical guidelines of grade recommendations are presented in Table 2, listing which probiotic types have strong or moderate evidence for specific diseases (either prevention or treatment indications) and which types were not as well supported (weak to no efficacy). For those readers wishing a more detailed presentation of data for each RCT, these data are presented in Supporting Information (S1 Dataset), along with the citations of included trials (S1 References).

**Table 2:** Graded recommendations for probiotic formulations for the prevention or treatment of 19 different types of diseases.

Type of disease	No. of study arms	Strong evidence* (no. + RCTs/no. negative RCTs)	Moderate evidence* (no. + RCTs/no. negative RCTs)	Weak to not effective* (no. + RCTs/no. negative RCTs)
**Prevention**				
Allergy	3	None	None	*L*. *rhamnosus* GG (1+/2-)
Antibiotic-Associated Diarrhea (AAD)	61	*S boulardii* I-745 (18+/9-) LaLcLr mix (3+/1-) *L casei* DN114001 (2+/0-)	*E*. *faecalis* SF38 (2+/1-)	LhLr mix (3+/3-) *L*. *rhamnosus* GG (4+/6-) *C*. *butyricum* 588 (1+/2-) *L*. *acidophilus* La5 + *B*. *lactis* Bb12 (1+/5-)
Prevention *C*. *difficile* infections (CDI)	23	None	None	*S*. *boulardii* (1+/11-) LaLcLr mix (2+/2-) *L*. *rhamnosus* GG (1+/4-) *L casei* 114001 (1+/1-)
*H*. *pylori* (side effects of therapy)	16	*S*. *boulardii* I-745 (7+/2-) LhLr mix (2+/0-)	*L*. *rhamnosus* GG (3+/2-)	None
Enteral feed associated diarrhea	3	*S*. *boulardii* I-745 (3+/0-)	None	None
Necrotizing Enterocolitis (NEC)	17	*L*. *rhamnosus* GG + lactoferrin (2+/0-) *B*. *infantis*+ *B*. *lactis*+ *Strept*. *thermophilus* (2+/0-)	None	*L*. *rhamnosus* GG (0+/2-) *S*. *boulardii* (0+/3-) *L*. *reuteri* 17938 (1+/3-) *B*. *lactis* Bb12 (0+/2-) *L*. *acidophilus*+ *B*. *bifidum* (1+/1-)
Nosocomial infections	2	None	None	*L*. *rhamnosus* GG (1+/1-)
Respiratory tract infections	10	None	None	*L*. *rhamnosus* GG (3+/3-) *L*. *casei* 114001 (2+/2-)
Surgical infections	8	Synbiotic PpLmLpLp (4+/1-)	None	*L*. *plantarum* 299v (1+/2-)
Traveler’s diarrhea	7	*S*. *boulardii* I-745 (4+/1-)	None	*L rhamnosus* GG (1+/1-)
Urinary Tract Infections	3	None	None	*L rhamnosus* GG (0+/3-)
**Treatment**				
Adult acute diarrhea	9	*S*. *boulardii* I-745 (4+/2-)	*E*. *faecalis* SF68 (2+/1-)	None
*C*. *difficile* recurrence	4	*S*. *boulardii* I-745 (2+/0-)	None	*L*. *rhamnosus* GG (0+/2-)
Colic	4	*L*. *reuteri* 17938 (4+/0-)	None	None
Constipation	3	None	*B*. *lactis* 173010 (2+/1-)	None
*H*. *pylori* eradication	35	LhLr mix (4+/1-)	*L*. *acidophilus* La5 + *B*. *lactis* Bb12 mix (3+/2-)	*S*. *boulardii* I-745 (4+/11-) *L*. *rhamnosus* GG (0+/4-) *L*. *acidophilus* LB (1+/2-) *C*. *butyricum* 588 (0+/3-)
Inflammatory Bowel Disease (IBD)	25	8-strain mix (8+/2-)	*S*. *boulardii* I-745 (2+/1-)	*L*. *rhamnosus* GG (1+/6-) *E*. *coli* Nissle (0+/5-)
Irritable Bowel Syndrome (IBS)	23	*L*. *plantarum* 299v (4+/1-) *B*. *infantis* 35624 (3+/1-)	None	*L*. *rhamnosus* GG (2+/2-) *S*. *boulardii* I-745 (2+/2-) 8-strain mix (2+/2-) *B*. *lactis* 173010 (1+/1-)
Pediatric acute diarrhea	61	*S*. *boulardii* I-745 (25+/4-) *L*. *rhamnosus* GG (10+/3-) *L*. *reuteri* 17938 (3+/0-) *L*. *acidophilus* LB (3+/1-) *L*. *casei* DN114001 (3+/0-) *Bac*. *clausii* mix (O/C, N/R84, T84, Sin8 (3+/1-) 8-strain mix (2+/0-)	LhLr (2+/1-)	None

*Strong evidence: net of ≥2 more RCT with significant findings; *moderate* evidence net of 1 more RCT with significant findings; *weak*, same number of significant and non-significant trials and *not effective*, net of >1 RCT with non-significant findings compared to studies with significant findings.

**Abbreviations**: B., *Bifidobacterium*; Bac., *Bacillus*; C., *Clostridium*; E., *Enterococcus*; H., *Helicobacter*; LaLcLr mix, *L*. *acidophilus* CL1285 *+L*. *casei* Lbc80r + *L*. *rhamnosus* CLR2 (Bio-K+); LhLr mix, *L*. *helveticus* R52 (CNCM I-1722) + *L*. *rhamnosus* R11 (CNCM I-1720), Lacidofil; S., *Saccharomyces*; Synbiotic PpLmLpLp, *Pediococcus pentosaceus* 5–33:3, *Leuconostoc mesenteroides* 77:1, *L*. *paracasei* ssp. *paracasei* F19, *L*. *plantarum* 2362 and four fibers (inulin, oat bran, pectin, starch); 8-strain mix (*Bifido*.*longum* BL03, *Bifido*. *infantis* subsp. *lactis* BI04, *Bifido*. *breve* BB02, *L*. *acidophilus* BA05, *L*. *plantarum* BP06, *L*. *paracasei* BP07, *L*. *helveticus* BD08, *Strept thermophiles* BT01, VSL#3)

#### Prevention of antibiotic-associated diarrhea (AAD)

As an example, we first examine the evidence for different probiotics for the prevention of antibiotic-associated diarrhea. AAD is defined as diarrhea (typically ≥3 loose/watery stools/day for at least two days) that occurs during the admission of oral or intravenous antibiotics (immediate onset AAD) or ≤4–8 weeks post-antibiotic discontinuation (delayed onset AAD). AAD may occur commonly in 5–50% of patients after use of almost any type of antibiotic, which disrupts the protective normal microbiome of the host. The consequences of AAD may include prolonged hospitalization stays for inpatients, increased healthcare costs, higher risk of acquiring other nosocomial infections and poor compliance (especially for outpatients), leading to inadequate cure rates due to shortened antibiotic treatment [33]. Of the 59 trials (61 study arms) testing probiotics for the prevention of AAD, there were eight different types of probiotics tested with ≥ 2 RCTs. Determining which probiotic is best for AAD is challenging due to differences in probiotic strains, doses and types of study populations. However, as shown in Table 2, of the eight different types of probiotics, three probiotic types [*S*. *boulardii* CNCM I-745, a mixture of three Lactobacilli strains (*L*. *acidophilus* CL1285 *+L*. *casei* Lbc80r + *L*. *rhamnosus* CLR2) and another single strain probiotic (*L*. *casei* DN114001) had strong evidence for the prevention of AAD, while one showed only moderate evidence (*E*. *faecalis* SF68). Effective probiotics were typically started 1–2 days of antibiotic admission with daily doses ranging from 10^7^−10^10^ per day.and continued for 1–4 weeks after antibiotics were discontinued. Four other probiotics had more studies with non-significant findings compared to significant studies and thus should not be considered as effective therapies for the prevention of AAD until further confirmatory trials show significant efficacy. These trials are described in detail in supporting information (S1 Dataset).

#### Prevention of *Clostridium difficile* infections (CDI)

*Clostridium difficile* accounts for approximately one-third of the cases of AAD, but may result in more severe disease (colitis, pseudomembranous colitis toxic megacolon), higher mortality rates and is the leading cause of nosocomial infections [34]. The incidence of CDI continues to increase in hospitals and long-term care facilities, causing over 14,000 deaths in the United States each year and adding $1 billion in healthcare costs [35]. CDI is diagnosed based on both the symptomatic status of the patient and the presence of at least one of the toxins of *C*. *difficile* in the stool. Probiotic strains or mixtures found effective for preventing AAD have been assessed in RCTs for their ability to prevent CDI, but only a few have shown promise [36].

There were only two studies testing probiotics as primary prevention therapies in randomized controlled trials. Most of the data on CDI was extracted from randomized trials for AAD that reported CDI as a secondary outcome. CDI occurs less frequently than AAD and thus these trials were not powered to assess CDI and often suffered from a limited number of CDI cases and an inability to determine if the probiotic was effective for CDI [37]. As shown in Table 2, several of the probiotics had more or equal numbers of non-significant outcomes (*S*. *boulardii* CNCM I-745, *L*. *rhamnosus* GG, *L*. *casei* DN114001 and the mixture of three Lactobacilli strains (*L*. *acidophilus* CL1285, *L*. *casei* Lbc80r, *L*. *rhamnosus* CLR2) by the number of individual studies. As the finding of non-significance may be due to the insufficient power in these studies, two meta-analyses of the pooled data provided more power and determined significant efficacy was present for three of the probiotics (*S*. *boulardii* CNCM I-745 and *L*. *casei* DN114001 and the mix of *Lactobacillus acidophilus* CL1285, *L*. *casei* LBC80R and *L*. *rhamnosus* CLR2) [38,39].

Another strategy we found in the literature was the use of quasi-experimental interventions, but we did not include them in evaluating the strength of the evidence. Primary prevention of CDI has typically rested upon enhanced infection control bundles and antibiotic stewardship programs [37]. Recently, facilities have added a specific probiotic to their infection control bundles to test if this would reduce the incidence of CDI. Only one type of probiotic had multiple studies to confirm their findings. From a RCT testing two different doses of a mixture of *Lactobacillus acidophilus* CL1285, *L*. *casei* LBC80R and *L*. *rhamnosus* CLR2, a significant reduction of CDI for both the low-dose (9.4%, p = 0.03) and the high dose (1.2%, p = 0.002) compared with the control group (23.8%) was found [40]. As a consequence, one hospital in Canada which was experiencing a persistent large CDI outbreak gave this three-strain Lactobacilli mixture to all inpatients receiving antibiotics and found a significant reduction of CDI rates, which was sustained over 10 years of the program [41]. The reduction of CDI rates was also observed at other Canadian hospitals who added the three-strain Lactobacilli formulation to their infection control programs [42, 43].

#### Prevention of adverse reactions to *H*. *pylori* therapy

*H*. *pylori* infections may lead to serious consequences, but the onset of symptoms (dyspepsia, gastric cancer, etc.) has a long incubation period (10–20 years), so efforts to eradicate carriage of the pathogen has been the standard focus of treatment. Unfortunately, the treatment (2-3-types of antibiotics, along with a proton-pump inhibitor) has poor compliance due to the common occurrence of adverse reactions to the antibiotic treatment. In contrast to the ability to eradicate *H*. *pylori*, probiotics may have a more useful role in reducing the side-effects of *H*. *pylori* eradication therapy [18,19]. We included 14 RCTs (and two additional study arms) that reported adverse reaction data in their trials of the treatment of *H*. *pylori*. Two probiotics have strong strength of efficacy evidence for preventing adverse events associated with treatments for *H*. *pylori* (*S*. *boulardii* I-745 and the mixture of *L*. *helveticus* R52 + *L*. *rhamnosus* R11), while *L*. *rhamnosus* GG only showed moderate strength. Most of the adverse reactions were associated with the antibiotic treatment (AAD, nausea, vomiting or abdominal pain), which may result in poor compliance to the eradication therapy. In this case, the use of these three probiotics may be valuable in preventing side effects of therapy, but not be directly effective in eradicating the pathogen (*H*. *pylori*) itself.

#### Prevention of necrotizing enterocolitis (NEC)

NEC remains one of the most common cause of death and morbidity in preterm infants, especially for very low birth weight neonates (<1500 g) and is characterized by bowel wall necrosis and >27% require surgery to treat NEC [44]. Although recent meta-analyses have found significant reductions in NEC in trials with probiotics, recommendations for specific probiotics are lacking as the analysis often pooled together different types of probiotic strains [45, 46]. Of the 17 RCTs, six different probiotic types were tested for NEC prevention. Most did not show strong or moderate evidence of efficacy (Table 2) for reducing NEC. One mixture (*B*. *infantis*, *B*. *lactis*, *Strept*. *thermophilus*) showed strong evidence (two trials with significant efficacy) in preventing NEC (Table 2). However, when lactoferrin was added to *L*. *rhamnosus* GG, both trials showed significant reductions in NEC in contrast to two trials when *L*. *rhamnosus* GG was given alone (no efficacy found). Additional confirmatory trials with the other types of probiotics are needed before strong recommendations for use should be made.

#### Prevention of post-surgical infections

Post-surgerical infections may be amendable to probiotic therapy as the microflora is often disrupted due to common exposures associated with surgery (pre-operative antibiotic exposure, other medications, etc). We found eight RCTs that had sufficient numbers of trials to assess efficacy. One synbiotic (*Pediococcus pentosaceus* 5–33:3, *Leuconostoc mesenteroides* 77:1, *L*. *paracasei* ssp. *paracasei* F19, *L*. *plantarum* 2362 and four fibers (inulin, oat bran, pectin, starch) had strong evidence for the prevention of post-surgical infections, but the types of surgeries varied (S1 Dataset), while another type (*L*. *plantarum* 299v) had only weak evidence. The synbiotic started on the day of surgery and continued 1–2 weeks afterwards and was given at a higher dose (2–4 x 10^10^/day) compared to *L*. *plantarum* 299v (2 x 10^9^/d).

#### Prevention of traveler’s diarrhea

Traveler’s diarrhea affects as many as 24–40 million travelers worldwide and the mean duration ranges from 12 hours and 3.5 days. One incapacitation day results in a loss of $290–490 million dollars of lost revenue. Prevention options are limited and behavioral practices are often overlooked by travelers. Probiotics may offer a method of preventing this disease [47]. We found only five RCTs (two had additional treatment arms) and found strong evidence for *S*. *boulardii* CNCM I-745, while *L*. *rhamnosus* GG had weak evidence (Table 2). Typically, probiotics (2–5 x 10^9^ per day) were begun a few days before travel, continued during travel and then for 2–5 days afterwards to allow time for intestinal microflora restoration.

#### Prevention of other diseases

Five other disease indications had fewer RCTs or did not find any probiotics with strong or moderate evidence. One probiotic (*S*. *boulardii* I-745) had strong evidence from three RCTs for the prevention of diarrhea associated with nasogastric tube feedings (Table 2). The evidence for the prevention of four other diseases (allergy, nosocomial infections, respiratory tract infections and urinary tract infections is less robust and no probiotics were found to be strongly or moderately effective.

#### Treatment of pediatric acute diarrhea

Pediatric acute diarrhea is a leading cause of morbidity and mortality in developing countries and can be due to viral or bacterial etiologies. Latin-American guidelines now recommend some probiotics should be given along with oral rehydration therapy for the treatment of acute pediatric diarrhea [13]. Probiotics are often used to treat children with acute diarrhea, but the wide variety of probiotics tested has also led to confusion about the best choice of probiotic to use. Grandy *et al*. compared giving just the single strain of *S*. *boulardii* against a mixture containing *S*. *boulardii* and three other bacterial probiotic strains: *L*. *acidophilus* and *L*. *rhamnosus* and a Bifidobacterial species) and compared the duration of diarrhea against a placebo control group [48]. While the single strain *S*. *boulardii* group demonstrated a significant reduction in the mean duration of diarrhea compared to the placebo group (-1.1 day, p = 0.04), when *S*. *boulardii* was given along with the mixture of three other bacterial strains, it was less effective (-1 day, p = 0.06). Several meta-analyses have been conducted supporting these individual clinical trials and most have focused on just one type of probiotic. Feizizadeh *et al*. pooled 22 RCT that treated children with acute diarrhea, but only included studies that used the *S*. *boulardii* I-745 yeast probiotic [49]. From the pooled data of 17 trials that reported mean duration of diarrhea, *S*. *boulardii* significantly reduced the duration by 19.7 hours. Szajewska *et al*. included trials using *L*. *acidophilus* LB and found a significant mean reduction in diarrhea pooled from 4 trials was 21.6 hours [50]. Urbanska *et al*. limited their meta-analysis to three trials using *L*. *reuteri* DSM17938 and also found a significant mean reduction of diarrhea by 24.8 hours [51].

We found the most evidence in this indication, as 59 RCTs assessed eight different probiotics types (one trial tested three different probiotic types) and seven of the eight probiotic types had strong evidence for this disease indication. Two types of probiotics with over 10 RCTs showed strong evidence of efficacy (*S*. *boulardii* CNCM I-745 with 25 trials showing efficacy and four with non-significant findings and *L*. *rhamnosus* GG with 10 trials showing efficacy and three with non-significant findings). Five other probiotics have fewer total number of trials but also had at least two more RCTs with significant efficacy compared to the number of RCTs with non-significant outcomes: *L*. *reuteri* 17938, *L*. *acidophilus* LB, *L*. *casei* DN114001, and two mixtures (mix of 4 strains of *Bacillus clausii* (O/C, N/R84, T84, Sin8) and another mix of 8 strains, as shown in Table 2. Another mixture (*L*. *helveticus* R52 and *L*. *rhamnosus* R11) showed moderate evidence (two found significant efficacy while one did not).

#### Treatment of inflammatory bowel disease (IBD)

We found 25 RCTs treating chronic IBD, but only one had strong evidence of efficacy (the 8-strain mixture, VSL#3). This multi-strain mixture found significant improvement in IBD symptom scores in eight of ten trials. *S*. *boulardii* CNCM I-745 had moderate evidence in that two of three trials found significant improvement in IBD symptoms, but two other probiotics had more trials with no significant improvement (*L*. *rhamnosus* GG and *E*. *coli* Nissle) and should not be recommended for patients with IBD at this time.

#### Treatment of irritable bowel syndrome (IBS)

We found 21 RCTs for the control of irritable bowel syndrome (IBS) symptoms (one trial had three dose arms), Table 2. Two probiotics had strong evidence for the improvement of IBS symptoms (*L*. *plantarum* 299v and *B*. *infantis* 35624). Four other probiotics (*L*. *rhamnosus* GG, *S*. *boulardii* CNCM I-745, *B*. *lactis* 173010 and the 8-strain mix) had an equal number of trials with significant improvement in IBS and trials with no significant improvement and cannot be recommended for IBS (Table 2).

#### Eradication of *H*. *pylori*

*Helicobacter pylori* infections are a global concern, with a prevalence ranging from 70–90% in developing countries and 25–50% in developed countries [18,19]. Prolonged *H*. *pylori* carriage may result in an onset of symptoms in adults including gastric ulcers, gastritis and gastric cancer. The standard treatment combines two to three antibiotics with a proton-pump inhibitor, but treatment often fails due to poor compliance relating to a high incidence of side-effects of the antibiotic therapy. In addition to the finding that some probiotics did significantly reduce the incidence of side-effects, much to researchers’ surprise, some probiotics were also effective in increasing the eradication rate of *H*. *pylori*. McFarland *et al*. included 25 RCTs in their meta-analysis using six different types of single strain probiotics and found only one sub-group (*S*. *boulardii* CNCM I-745) significantly improved *H*. *pylori* eradication rates (RR = 1.11, 95% C.I. 1.07–1.16, p<0.05), while five other sub-groups of the same strain had no significant effect (*Clostridum butyricum* 588, *L*. *rhamnosus* GG, *L*. *acidophilus* nr, *L*. *reuteri* 17938 or *L*. *casei* 114001) [18]. Another meta-analysis of 19 RCTs separated probiotic mixtures into six sub-groups of the same multi-strain mixes and found four mixtures significantly increased *H*. *pylori* eradication rates (the mix of *L*. *acidophilus* La5 and *B*. *animalis lactis* Bb12; a mix of *L*. *helveticus* R52 and *L*. *rhamnosus* R11; a mix of *L*. *acidophilus* nr and *B*. *longum* nr and *E*. *faecalis* nr; and two RCTs of an eight-strain mixture), while two mixtures (a mix of *L*. *acidophilus* La5 and *B*. *bifidum* Bb12 and a mix of *L*. *acidophilus* 2177 and *L*. *casei* 27443 and *B*. *longum* 8001) had no significant effect on *H*. *pylori* eradication rates [19].

In our review, we found of 33 RCTs (two had an additional treatment arm) for the eradication of *H*. *pylori* (details found S1 Dataset). Two probiotic mixtures had either strong evidence (*L*. *helveticus* R52 and *L*. *rhamnosus* R11) or moderate evidence (*L*. *acidophilus* La5 and *Bifido*. *lactis* Bb12) for the eradication of *H*. *pylori*, while four other types of probiotics *(S*. *boulardii* CNCM I-745, *L*. *rhamnosus* GG, *L*. *acidophilus* LB and *C*. *butyricum* 588 had weak to no evidence of efficacy to eradicate *H*. *pylori*.

#### Treatment of *Clostridium difficile* infections

Probiotics have also been tested for as an adjunct treatment for CDI, given in addition to standard antibiotic treatments (vancomycin or metronidazole) and using the recurrence of CDI as an outcome. As many as 20–30% of patients with CDI may experience a recurrence of CDI and some may continue to have CDI episodes over a period of years [35]. Only two types of probiotics had at least two RCTs for CDI. Two trials found *S*. *boulardii* I-745 significantly reduced CDI recurrence rates [52,53], but the effect was strongest in those with recurrent CDI [52]. Two other RCTs with *L*. *rhamnosus* GG did not find any significant reductions in CDI recurrences [54, 55].

#### Treatment of other diseases

Three other diseases had evidence from ≥2 RCTs (adult acute diarrhea, pediatric colic and constipation), that show promising results for four types of probiotics (Table 2), but the number of studies is sparse. One probiotic (*L*. *reuteri* 17938) had strong evidence from four RCTs for the treatment of pediatric colic.

### Choosing a quality probiotic product

Once the specific probiotic strain or mixture of strains is selected for the disease being prevented or treated, the next challenge is finding a product containing those specific strains which is of reliable quality and stable. Factors associated with that choice include: formulation used, daily dose needed, quality control and manufacturing source.

#### Formulation

Probiotics are also available in a wide range of formulations (from yogurts to fermented beverages, to powders in sachets or lyophilized capsules or tablets), as shown in Table 1. There is scant evidence comparing which type of formulation may be more effective. The choice of formulation may be based on shelf-life, in that lyophilized capsules maintain high concentrations longer than probiotics in dairy products and enteric-coated capsules show higher survival rates compared to non-enteric coated capsules [56]. Probiotic capsules requiring refrigeration are heat-dried (not lyophilized) and thus not stable at room temperature, limiting their portability. In addition, if the patient is lactose-intolerant, yogurts or other types of fermented dairy products may be inadvisable.

#### Daily dose

Probiotics have been defined by having a health benefit, but also that it be given as an ‘adequate amount’, but the specific dose was not specified in guidelines [23]. The question of whether probiotics have a dose-response, similar to other medications, has been explored. Several reviews and meta-analyses have examined if there is a threshold for an effective oral dose of probiotics. One meta-analysis of 20 RCTs for the prevention of pediatric antibiotic-associated diarrhea (AAD) gave probiotics in doses ranging from 6 x 10^6^ to 3 x 10^10^ colony-forming units (cfu) per day and found a lower rate of AAD in those children given probiotics (~8%) compared to controls (17%), but the effect did not significant improve with increasing daily doses [17]. A meta-analysis by Goldenberg *et al*. analyzed 22 randomized controlled trials testing various probiotics for the prevention of pediatric AAD found an overall dose effect of increased efficacy for probiotic doses ≥ 5 x 10^9^ cfu/day, but pooled data below that dose were ineffective [57]. Ouwehand reviewed the literature and confirmed this dose-response (breakpoint of 10^10^ cfu/day) from seven meta-analyses of probiotics for AAD [58]. However, this review found a dose-response only for AAD and the reduction of blood pressure (breakpoint of >10^11^cfu/day), but not for any other disease indication, including *C*. *difficile* infections, necrotizing enterocolitis, prevention of atopic dermatitis, prevention of colorectal cancer, or the treatment of irritable bowel syndrome. Unfortunately, all these reviews combined different probiotic strains into dose groups, thus the findings may have been confounded by the type of probiotic product. Clear dose-response behavior is observed in pharmacokinetic studies of specific strains in dose-ranging studies, typically finding more fecal recovery of the probiotic as the oral dose is increased, but these studies did not document the effect of dose on clinical efficacy [58]. As most daily doses used in probiotic clinical trials are based on kinetic dose recovery studies, a lower limit where a loss of efficacy may be observed (<10^6^ cfu/day) is often not used in RCTs, which might explain why a demonstrable dose-response has not been documented.

#### Quality control

Lack of consistent regulations governing the quality of probiotic products has resulted in varying degrees of product quality. Several studies have found discrepancies from what was stated on the product label versus independent testing of the ingredients including lower levels of microbes, not finding the labeled strain or finding contamination of other strains in the product. In one study, of five “*Bacillus clausii*” products available in India and Pakistan, 80% had lower Bacilli concentrations when assayed than the numbers listed on the label and were also contaminated by non-*B*. *clausii* bacteria [59]. Chen *et al*. assayed 28 commercial probiotic products available in China and could only confirm 53–83% of the species listed on the labels and, of 16 products labeled with a Bifidobacterial species, none were found in any of the products [60]. Toscano *et al*. tested 24 probiotic products available in Europe and found 42% did not contain the species listed on the label and four products had no living microbes at all in the product [61]. In contrast, Goldstein *et al*. investigated five over-the-counter probiotic products and found the concentrations of all the microbes listed on the label were accurate [62]. Currently, an European advisory group (ESPGHAN) of experts has recognized the issue of varying quality and has called for improved quality control on all commercial probiotic products [11].

#### Manufacturer

Manufacturing processes, stability over time (shelf-life) and the type of formulation has been found to be significant factors in the effectiveness of the final probiotic product [7, 63]. Grzeskowiak *et al*. tested 13 different “LGG” products available in different formulations (4 capsulated, 2 infant foods, 3 lyophilized, 4 other types) and, while all had detectable *L*. *rhamnosus* GG strains identified, 38% of the products appeared to have lost the ability to inhibit pathogens *L*. *rhamnosus* GG normally inhibits and differences in the ability to adhere to the colonic mucosa were also observed for the different *L*. *rhamnosus* GG products [64]. Perhaps differences were due to manufacturing processes involved in the different formulations, but this was not investigated further. Quality control studies of probiotic products manufactured by established pharmaceutical-grade companies (such as Bio-K+, Florastor, Culturelle, etc.) have confirmed both accurate species/strain identification in the products, and also confirmed the concentration stated on labels and a lack of contamination by other microbial strains not listed on the labels [62, 65,66]. Probiotics sold on-line or over-the-counter that do not provide a manufacturing source or having a less established fermentation quality control history should be viewed with caution.

#### Safety

In the majority of the RCTs, patients are followed for any adverse events associated with the probiotic intervention. Although no serious adverse events were associated with most (80%) trials of probiotics, mild-moderate gastrointestinal symptoms (gas, abdominal bloating, etc.) were reported by some trials, while other trials did not report any adverse events [49,51,63]. Many RCTs simply reported ‘no adverse events were noted’, but did not report specific data. This was also noted in a Cochrane review of 384 studies of probiotics, prebiotics and synbiotics that found while 28% of studies reported no adverse events were noted, 37% failed to report any adverse event data in the results sections [67]. In another review investigating 24 trials for the safety of probiotics in variety of different 'at risk' populations (children, pregnant women, elderly, IBD patients, immunocompromised), only two cases of adverse events were associated with probiotics [68]. However infrequent cases of bacteremia or fungemia have been reported in seriously ill patients or neonates who were taking probiotics and thus, the use of probiotics in hospitalized patients with central catheters or who are immunocomprised may be contra-indicated [15, 69].

#### Practical guidance when choosing a probiotic product

The choice of the best probiotic for your patient will continue to be a shifting target, as more and more research and clinical trials are done. Data from this literature review indicates the first consideration is the mode of therapy (prevention or treatment), as different probiotics may be more appropriate for each goal (Fig 2). Different choices may also be more appropriate for pediatric versus adult populations. The most suitable single-strain or probiotic mixture may then be selected from the current knowledge and summary of clinical trials (Table 2). Depending upon availability and regulatory oversight in your country, other considerations may also assist the selection. For example, if two probiotic products are available with similar strains, reviewing how well the product complies with required label information might be useful. As shown in Fig 2, probiotics available as dietary supplements should list the FDA disclaimer, manufacturer, probiotic strains in the product, daily dose and comply with FDA regulated health/function claims. Products not listing these components should be viewed with caution. If the probiotic is regulated as a prescription medicine, seek the advice of a pharmacist or physician with knowledge in this field.

## Discussion

We screened over 2545 articles and abstracts on probiotics and found 22 different probiotic formulations with an evidence base of at least two randomized controlled trials within a specific disease indication (94 trials for the prevention of disease and 155 trials for the treatment of disease). Two disease indications had a robust number of RCTs for specific probiotic types (prevention of AAD with 61 study arms and treatment of pediatric acute diarrhea with 61 study arms. Strong evidence for efficacy was found for three probiotics to prevent AAD: *S*. *boulardii* I-745 or *L*. *casei* DN114001 or the multi-strain formulation of *Lactobacillus acidophilus* CL1285, *L*. *casei* LBC80R and *L*. *rhamnosus* CLR2). These probiotics should be used at a dose of 1–2 x 10^10^ cfu/day, starting within 48 hours of the antibiotic and continued for 5–7 days after the antibiotic has been discontinued. Efficacy for the treatment of pediatric acute diarrhea was strongly supported for *S*. *boulardii* I-745, *L*. *rhamnosus* GG, *L*. *reuteri* 17938, *L*. *acidophilus* LB, *L*. *casei* DN114001, *Bacillus clausii* and an eight-strain mixture (VSL#3) and moderately supported for a two-strain mixture (*L*. *helveticus* R52 and *L*. *rhamnosus* R11). Strong efficacy evidence was also found for an eight-strain mixture (VSL#3) for the treatment of IBD, while *L*. *plantarum* 299v or *B*. *infantis* 35624 was better for IBS. The probiotics for treatment indications should be given at a dose of 1–2 x 10^10^ cfu/day until symptoms resolve or go into remission.

The number of practical guidelines for probiotic use that provide strain-specific and disease-specific recommendations are sparse. Most reviews have focused on one specific disease indication, such as probiotics for the prevention of *C*. *difficile* disease [36] or prevention of antibiotic-associated diarrhea [70]. A guide for probiotic use in lower gastrointestinal disease by Hungin *et al*. recommended some specific probiotic strains for irritable bowel syndrome and antibiotic-associated diarrhea, but no other diseases [71].

International guidelines reinforce the importance of determining probiotic efficacy based on specific strain or strains in a probiotic formulation [72–75]. However, when seeking advice from published reviews on which strain is most effective for a specific indication, many reviews and meta-analyses have inappropriately pooled together strains into broad categories, such as ‘probiotics containing a Lactobacilli strain’ [76]. Another meta-analysis of 30 RCTs using probiotics in preterm infants concluded that ‘severe necrotizing enterocolitis rates (stage II or more) and all-cause mortality were reduced among probiotics groups’, but they pooled the results for 20 different types of probiotics and did not account for the strain-specificity of probiotics, so it is unclear which of the 20 probiotics might be effective and which ones are not effective [46].

The strengths of our review included that we conducted a comprehensive search (including clinical trial databases and meeting websites for abstracts) of the literature, but only included probiotic formulations with at least two randomized, controlled efficacy trials and we did not exclude foreign language articles. Previous guidelines either have not included the number of trials their recommendations were based on or based their recommendations on finding at least two randomized controlled trials, regardless of the outcome. Ours is the first to look at the net balance of significant versus non-significant trials and to account for three important factors to consider when choosing an appropriate probiotic for a patient: disease mode (prevention or treatment), strain-specificity and disease-specificity.

Limitations of our review included: (1) some probiotic products did not have sufficient numbers of randomized controlled trials for each disease indication to be included and (2) due to our inclusion requirement of at least two RCTs/probiotic type per disease, there were also some diseases or conditions (acne, asthma, arthritis, cholera, cystic fibrosis, dental infections, diabetes, general gastrointestinal health, hypertension, pancreatitis and weight control, etc.) that had only one RCT, but could not be included in our review due to the sparcity of RCTs for specific probiotic types within each disease type, (3) we also summarized results over different study populations (inpatient versus outpatients, adults versus children) and with a variety of of doses and durations of probiotic interventions and (4) we did not evaluate risk of bias for each study nor present detailed analysis of each outcome in this practical guide. However, we have presented detailed data on each study in the Supporting Information (S1 Dataset). Another challenge in evaluating the efficacy for some diseases was the variety of different types of outcomes used to assess efficacy. For example, trials with IBS used different outcomes (number of ‘responders’ or change in IBS-symptom scores, or changes in specific IBS symptoms). Trials documenting outcomes for the treatment of pediatric acute diarrhea may have included: mean duration of diarrhea or reported ‘cured’ based on symptoms. The outcomes for other diseases were more uniformed over the trials (for example, *H*. *pylori* eradication trials, recurrences of CDI, crying times for pediatric colic). The diversity of outcomes within one type of disease presents a challenge for meta-analyses or systematic reviews. Another limitation is that these recommendations for specific probiotics are not static, as the results may change as more trials are published. Probiotics that currently have weak or no efficacy results may change to moderate or strong evidence as the results of more RCTs are detremined.

## Conclusions

Determining the appropriate probiotic for your patient can be a daunting and confusing decision. Our review found the efficacy of probiotics is largely dependent upon three factors: mode-specificity, strain-specificity and disease-specificity. Randomized trials are the preferred avenue to achieving reliable recommendations for probiotic applications across the spectrum of diseases for which they are currently being utilized and more trials are needed. Future studies should recognize the need to carefully define the probiotic strain or strains in their intervention, as efficacy is clearly tied to the probiotic strain.

## Supporting information

S1 PRISMA Checklist PRISMA checklist. (DOCX)

S1 Text An example of a search strategy. (DOCX)

S1 References Citations of included trials by disease indication. (DOCX)

S1 Dataset McFarland dataset for probiotic trials. (DOCX)
