# Supplementary material for: Choosing an appropriate probiotic product for your patient: An evidence-based practical guide
Source: PLoS One. 2018 Dec 26;13(12):e0209205. doi: 10.1371/journal.pone.0209205 (PMC6306248; doi:10.1371/journal.pone.0209205)
Supplement: S1 Text — (DOCX) [file pone.0209205.s002.docx]

**S2. Text. An example of a search strategy.**

(("**Probiotics**"[Mesh] OR ("**probiotics**"[MeSH Terms] OR "**probiotics**"[All Fields])) OR ("**probiotics**"[MeSH Terms OR "**probiotics**"[All Fields] OR "**probiotic**"[All Fields])) OR **bifidobacteria**[All Fields]) OR ("**bifidobacterium**"[MeSH Terms] OR "**bifidobacterium**"[All Fields])) OR ("**lactobacillaceae**"[MeSH Terms] OR "**lactobacillaceae**"[All Fields])) OR **lactobacilli**[All Fields]) OR ("**lactobacillus**"[MeSH Terms] OR "**lactobacillus**"[All Fields])) OR ("**saccharomyces**"[MeSH Terms] OR "**saccharomyces**"[All Fields]) OR ("**AAD**"[MeSH Terms] OR "**AAD**"[All Fields]) OR ("**CDI**"[MeSH Terms] OR "**CDI**"[All Fields]) OR ("**Clostridium difficile**"[MeSH Terms] OR "**Clostridium difficile**"[All Fields]) OR ("**irritable bowel syndrome**"[MeSH Terms] OR "**irritable bowel syndrome** "[All Fields]) OR ("**H. pylori infection**"[MeSH Terms] OR "**H. pylori infection** "[All Fields]) AND (((((("**prevention and control**"[Subheading] OR ("**prevention and control**"[Subheading] OR ("**prevention**"[All Fields] AND "**control**"[All Fields]) OR "**prevention and control**"[All Fields] OR "**prevention**"[All Fields])) OR **preventing**[All Fields]) OR **preventive**[All Fields]) OR ("**prevention and control**"[Subheading] OR ("**prevention**"[All Fields] AND "**control**"[All Fields]) OR "**prevention and control**"[All Fields] OR "**prophylaxis**"[All Fields])) OR ("**prevention and control**"[Subheading] OR ("**prevention**"[All Fields] AND "**control**"[All Fields]) OR "**prevention and control**"[All Fields] OR "**control**"[All Fields] OR "**control groups**"[MeSH Terms] OR ("**control**"[All Fields] AND "**groups**"[All Fields]) OR "**control groups**"[All Fields]))
