# Supplementary material for: Choosing an appropriate probiotic product for your patient: An evidence-based practical guide
Source: PLoS One. 2018 Dec 26;13(12):e0209205. doi: 10.1371/journal.pone.0209205 (PMC6306248; doi:10.1371/journal.pone.0209205)
Supplement: S1 References — (DOCX) [file pone.0209205.s003.docx]

**S3 References. Citations of included trials by disease indication**

**Prevention of Allergies (3 RCT)**

Helin T, Haahtela S, Haahtela T. No effect of oral treatment with an intestinal bacterial strain, *Lactobacillus rhamnosus* (ATCC 53103), on birch-pollen allergy: a placebo-controlled double-blind study. Allergy. 2002;57(3):243-6. doi: 10.1034/j.1398-9995.2002.1s3299.x

Kalliomaki M, Salminen S, Arvilommi H, Kero P, Koskinen P, Isolauri E. Probiotics in primary prevention of atopic disease: a randomized placebo-controlled trial. Lancet. 2001; 357: 1076-9. doi: 10.1016/S0140-6736(00)04259-8

[Kopp MV, Hennemuth I, Heinzmann A, Urbanek R.](http://www.ncbi.nlm.nih.gov/pubmed/18332075?ordinalpos=1&itool=EntrezSystem2.PEntrez.Pubmed.Pubmed_ResultsPanel.Pubmed_RVDocSum) Randomized, double-blind, placebo-controlled trial of probiotics for primary prevention: no clinical effects of *Lactobacillus* GG supplementation. Pediatrics. 2008;121(4):e850-6. 10.1542/peds.2007-1492

**Prevention of Antibiotic associated diarrhea (59 RCT, 61 treatment arms)**

*red indicates AAD was secondary outcome in the trial

Adam J, Barret C, Barret-Bellet A, Benedetti E, Calendini A, Darchen P, et al. [Controlled double-blind clinical trials of Ultra-Levure: multicentre study by 25 physicians in 388 cases.] Gazette Medicale de France. 1977; 84: 2072-8.

Armuzzi A, Cremonini F, Ojetti V, Bartolozzi F, Canducci F, Candelli M, et al. Effect of Lactobacillus GG supplementation on antibiotic-associated gastrointestinal side effects during *Helicobacter pylori* eradication therapy: a pilot study. Digestion. 2001A;63(1):1-7. doi: [10.1159/000051865](https://doi.org/10.1159/000051865)

Armuzzi A, Cremonini F, Bartolozzi F, Canducci F, Candelli M, Ojetti V, et al. The effect of oral administration of *Lactobacillus* GG on antibiotic-associated gastrointestinal side-effects during *Helicobacter pylori* eradication therapy. Aliment Pharmacol Ther. 2001B; 15(2): 163-169. doi: 10. [.1046/j.1365-2036.2001.00923.x](https://doi.org/10.1046/j.1365-2036.2001.00923.x)

Arvola T, Laiho K, Torkkeli S, Mykkanen H, Salminen S, Maunula L, et al. Prophylactic *Lactobacillus* GG reduces antibiotic-associated diarrhea in children with respiratory infections: a randomized study. Pediatrics. 1999; 104 (5):e64.

Aryayev M, Kononenko N. Prevention of antibiotic-associated diarrhoea in patients with cystic fibrosis. Odessa Medical Journal. 2009; 4(114): 78. [Lacidofil]

Beausoleil M, Fortier N, Guénette S, L'ecuyer A, Savoie M, Franco M, et al. Effect of a fermented milk combining *Lactobacillus acidophilus* Cl1285 and *Lactobacillus casei* in the prevention of antibiotic-associated diarrhea: a randomized, double-blind, placebo-controlled trial. Can J Gastroenterol. 2007;21(11):732-6.

Benhamou PH, Berlier P, Danjou G, Plique O, Jessueld D, Dupont C. Antibiotic-associated diarrhea in children: a computer monitored double-blind trial comparing a protective agent and a probiotic. Med Chir Dig. 1999; 28:163-168.

[Bin Z](http://www.ncbi.nlm.nih.gov/pubmed/?term=Bin%20Z%5BAuthor%5D&cauthor=true&cauthor_uid=25866729), [Ya-Zheng X](http://www.ncbi.nlm.nih.gov/pubmed/?term=Ya-Zheng%20X%5BAuthor%5D&cauthor=true&cauthor_uid=25866729), [Zhao-Hui D](http://www.ncbi.nlm.nih.gov/pubmed/?term=Zhao-Hui%20D%5BAuthor%5D&cauthor=true&cauthor_uid=25866729), [Bo C](http://www.ncbi.nlm.nih.gov/pubmed/?term=Bo%20C%5BAuthor%5D&cauthor=true&cauthor_uid=25866729), [Li-Rong J](http://www.ncbi.nlm.nih.gov/pubmed/?term=Li-Rong%20J%5BAuthor%5D&cauthor=true&cauthor_uid=25866729), [Vandenplas Y](http://www.ncbi.nlm.nih.gov/pubmed/?term=Vandenplas%20Y%5BAuthor%5D&cauthor=true&cauthor_uid=25866729).The Efficacy of *Saccharomyces boulardii* CNCM I-745 in addition to standard *Helicobacter pylori* eradication treatment in children. Pediatr Gastroenterol Hepatol Nutr. 2015;18(1):17-22. doi: 10.5223/pghn.2015.18.1.17

Borgia M, Sepe N, Brancato V, Costa G, Simone P, Borgia R, et al. A controlled clinical study on *Streptococcus faecium* preparation for the prevention of side reactions during long term antibiotic treatments. Curr Therap Res. 1982; 31: 265-271.

Bravo MV, Bunout D, Leiva L, de la Maza MP, Barrera G, de la Maza J, et al. Effect of probiotic *Saccharomyces boulardii* on prevention of AAD in adult outpatients with amoxicillin treatment. Rev Med Chile. 2008:136:981-988.

Can M, Beşirbellioglu BA, Avci IY, Beker CM, Pahsa A. Prophylactic *Saccharomyces boulardii* in the prevention of antibiotic-associated diarrhea: a prospective study. Med Sci Monit. 2006;12(4):19-22.

Casem RA. *S. boulardii* in the prevention of antibiotic-associated diarrhea in children: a randomized controlled trial. Phili Infect Dis Soc Proceed J. 2013;13(2):70.

Chatterjee S, Kar P, Das T, Ray S, Gangulyt S, Rajendiran C, et al. Randomised placebo-controlled double blind multicentric trial on efficacy and safety of *Lactobacillus acidophilus* LA-5 and *Bifidobacterium* BB-12 for prevention of antibiotic-associated diarrhoea. J Assoc Physicians India. 2013;61(10):708-12.

Chu Y, Zhu H, Zhou Y, Lv L, Huo J. Intervention study on *Saccharomyces boulardii* with proton pump inhibitor (PPI)-based triple therapy for *Helicobacter pylori* related peptic ulcer. African J Pharmacy Pharmacol. 2012; 6 (41):2900-2904. doi: 10.5897/AJIPP12.400

[Cindoruk M, Erkan G, Karakan T, Dursun A, Unal S.](http://www.ncbi.nlm.nih.gov/pubmed/17669103?ordinalpos=6&itool=EntrezSystem2.PEntrez.Pubmed.Pubmed_ResultsPanel.Pubmed_RVDocSum) Efficacy and safety of *Saccharomyces boulardii* in the 14-day triple anti-*Helicobacter pylori* therapy: a prospective randomized placebo-controlled double-blind study. Helicobacter. 2007;12(4):309-16. doi: 10.1111/j.1523-5378.2007.00516.x

Cremonini F, Di Caro S, Covino M, Armuzzi A, Gabrielli M, Santarelli L, et al. Effect of different probiotic preparations on anti-*Helicobacter pylori* therapy-related side effects: a parallel group, triple blind, placebo-controlled study. Am J Gastroenterol. 2002;97(11):2744-9. doi: 10.1097/MCG.0b013e3182166a42 [2 treatment arms]

De Vrese M, Kristen H, Rautenberg P, Laue C, Schrezenmei J. Probiotic lactobacilli and bifidobacteria in a fermented milk product with added fruit preparation reduce antibiotic associated diarrhea and *Helicobacter pylori* activity. J Dairy Res. 2011; 78(4):396-403. doi: 10.1017/S002202991100063X

Dietrich CG, Kottmann T, Alavi M. Commercially available probiotic drinks containing *Lactobacillus casei* DN-114001 reduce antibiotic-associated diarrhea. World J Gastrol. 2014:20(42):15837-44. doi: 10.3748/wjg.v20.i42.15837

[Duman DG](http://www.ncbi.nlm.nih.gov/sites/entrez?Db=pubmed&Cmd=Search&Term=%22Duman%20DG%22%5BAuthor%5D&itool=EntrezSystem2.PEntrez.Pubmed.Pubmed_ResultsPanel.Pubmed_DiscoveryPanel.Pubmed_RVAbstractPlus), [Bor S](http://www.ncbi.nlm.nih.gov/sites/entrez?Db=pubmed&Cmd=Search&Term=%22Bor%20S%22%5BAuthor%5D&itool=EntrezSystem2.PEntrez.Pubmed.Pubmed_ResultsPanel.Pubmed_DiscoveryPanel.Pubmed_RVAbstractPlus), [Ozütemiz O](http://www.ncbi.nlm.nih.gov/sites/entrez?Db=pubmed&Cmd=Search&Term=%22Oz%C3%BCtemiz%20O%22%5BAuthor%5D&itool=EntrezSystem2.PEntrez.Pubmed.Pubmed_ResultsPanel.Pubmed_DiscoveryPanel.Pubmed_RVAbstractPlus), [Sahin T](http://www.ncbi.nlm.nih.gov/sites/entrez?Db=pubmed&Cmd=Search&Term=%22Sahin%20T%22%5BAuthor%5D&itool=EntrezSystem2.PEntrez.Pubmed.Pubmed_ResultsPanel.Pubmed_DiscoveryPanel.Pubmed_RVAbstractPlus), [Oğuz D](http://www.ncbi.nlm.nih.gov/sites/entrez?Db=pubmed&Cmd=Search&Term=%22O%C4%9Fuz%20D%22%5BAuthor%5D&itool=EntrezSystem2.PEntrez.Pubmed.Pubmed_ResultsPanel.Pubmed_DiscoveryPanel.Pubmed_RVAbstractPlus), [Iştan F](http://www.ncbi.nlm.nih.gov/sites/entrez?Db=pubmed&Cmd=Search&Term=%22I%C5%9Ftan%20F%22%5BAuthor%5D&itool=EntrezSystem2.PEntrez.Pubmed.Pubmed_ResultsPanel.Pubmed_DiscoveryPanel.Pubmed_RVAbstractPlus), et al. Efficacy and safety of *Saccharomyces boulardii* in prevention of antibiotic-associated diarrhoea due to *Helicobacter pylori* eradication. [Eur J Gastroenterol Hepatol*.*](javascript:AL_get(this,%20'jour',%20'Eur%20J%20Gastroenterol%20Hepatol.');) 2005;17(12):1357-1361.

Ehrhardt S, Guo N, Hinz R, Schoppen S, May J, Reiser M, et al. *Saccharomyces boulardii* to prevent antibiotic-associated diarrhea: A randomized, double-masked, placebo-controlled trial. Open Forum Infect Dis. 2016;29;3(1):1-7. doi: 10.1093/ofid/ofw011

Erdeve O, Tiras U, Dallar Y, Savas S. *Saccaromyces boulardii* and antibiotic-associated diarrhoea in children. Aliment Pharmacol Therap. 2005;21(12):1508-9.

Evans M, Salewski RP, Christman MC, Girard SA, Tompkins TA. [Effectiveness of *Lactobacillus helveticus* and *Lactobacillus rhamnosus* for the management of antibiotic-associated diarrhoea in healthy adults](https://www.optibacprobiotics.co.uk/uploads/evans-2016-l-helveticus-l-rhamnosus-for-management-of-antibiotic-associated-diarrhoea.pdf): a randomised, double-blind, placebo-controlled trial. British Journal of Nutrition. 2016;116(1):94-103. doi: 10.1017/S0007114516001665

Frigerio G. A lactic acid producing *Enterococcus* in the prevention of antibiotic-associated diarrhea and in the treatment of acute diarrheal disorders. A double blind multicenter placebo-controlled clinical study. Dig Dis Sci. 1986;31(10 Suppl): 496S. Meeting abstract.

Gao XW, Mubasher M, Fang CY, Reifer C, Miller LE. [Dose-response efficacy of a proprietary probiotic Formula of *Lactobacillus acidophilus* CL1285 and *Lactobacillus casei* LBC80R for antibiotic-associated diarrhea and *Clostridium* *difficile*-associated diarrhea prophylaxis in adult patients.](http://www.ncbi.nlm.nih.gov/pubmed/20145608?itool=EntrezSystem2.PEntrez.Pubmed.Pubmed_ResultsPanel.Pubmed_RVDocSum&ordinalpos=6) Am J Gastroenterol. 2010;105(7):1636-41. doi: 10.1038/ajg.2010.11

Gnaytenko O, Llychkovska O, Kulachkovska Y, Semen V. [Antibiotic-associated diarrhea as a complication of antihelicobacter therapy in children.] Practical Medicine. 2009; 5:76-83. [in Ukranian]

[Hickson M, D'Souza AL, Muthu N, Rogers TR, Want S, Rajkumar C, et al](http://www.ncbi.nlm.nih.gov/pubmed/17604300?ordinalpos=3&itool=EntrezSystem2.PEntrez.Pubmed.Pubmed_ResultsPanel.Pubmed_RVDocSum). Use of probiotic *Lactobacillus* preparation to prevent diarrhoea associated with antibiotics: randomised double blind placebo controlled trial. Br Med J 2007;335(7610):80-3. doi: 10.1016/j.jclinepi.2010.09.011

[Imase K, Takahashi M, Tanaka A, Tokunaga K, Sugano H, Tanaka M, et al.](http://www.ncbi.nlm.nih.gov/pubmed/18402597?ordinalpos=1&itool=EntrezSystem2.PEntrez.Pubmed.Pubmed_ResultsPanel.Pubmed_RVDocSum) Efficacy of *Clostridium butyricum* preparation concomitantly with *Helicobacter pylori* eradication therapy in relation to changes in the intestinal microbiota. Microbiol Immunol. 2008;52(3):156-61. doi:10.1111/j.1348-0421.2008.00026.x

[Kotowska M, Albrecht P, Szajewska H.](http://www.ncbi.nlm.nih.gov/pubmed/15740542?ordinalpos=3&itool=EntrezSystem2.PEntrez.Pubmed.Pubmed_ResultsPanel.Pubmed_RVDocSum) *Saccharomyces boulardii* in the prevention of antibiotic-associated diarrhoea in children: a randomized double-blind placebo-controlled trial. Aliment Pharmacol Ther. 2005;21(5):583-590. doi: 10.1111/j.1365-2036.2005.02356.x

Kyriakos N, Papmichael K, Roussos A, Therodoropoulos I, Karakoidas C, Smyrnidis A, et al. Lyophilized form of *Saccharomyces boulardii* enhances the *Helicobacter pylori* eradication rates of omeprazole-triple therapy in patients with peptic ulcer disease or functional dyspepsia. Hosp Chronicles. 2013; 8(3):127-133.

Lee JY, Shoi SP, Park JH, Shin JW, Joo YH. The effect of *Saccharomyces boulardii* as an adjuvant to the 14-day triple therapy for eradication of *Helicobacter pylori*. Abstract #44. Presented at Asian Pacific Digestive Week, 1-4 October 2011, SUNTEC Singapore. *J Gastro & Hepatol*. 2011;26(S5):257.

Lewis SJ, Potts LF, Barry RE. The lack of therapeutic effect of *Saccharomyces boulardii* in the prevention of antibiotic-related diarrhoea in elderly patients. J Infect. 1998; 36: 171-4.

Liskovich V, Naumov I, Ganchar E, Dembrovskaya S. Efficiency of Lacidofil-WM for prevention of vaginal dysbiosis and antibiotics-associated diarrhoea in puerperas after caesarean operation. Health. 2010; 1: 63-66.

Marushko Y, Shef G. Current status of antibiotics-associated bowel disorders issue in children. Perinatology and Pediatrics. 2007; 4: 65-68.

McFarland LV, Surawicz CM, Greenberg RN, Elmer GW, Moyer KA, Melcher SA, Bowen KE, Cox JL. Prevention of β-lactam-associated diarrhea by *Saccharomyces boulardii* compared to placebo. Am J Gastroenterol. 1995; 90: 439-48.

Miller M, Florencio S, Eastmond J, Reynolds S. Results of 2 prospective randomized studies of Lactobacillus GG to prevent *C. difficile* infection in hospitalized adults receiving antibiotics. Abstract of the 48th Interscience Conference on Antimicrobial Agents and Chemotherapy, Washington DC 2008B;48:578-579.

Mirzaee V, Rezahosseini O. Randomized control trial: Comparison of triple therapy plus probiotic yogurt vs. standard triple therapy on *Helicobacter pylori* eradication. Iran Red Crescent Med J. 2012;14(10):657-66.

Monteiro E, Fernandes JP, Vieira MR, Correia JP, Caetano JM, Ribeiro T, et al. [Double blind clinical trial on the use of ultra-levure in the prophylaxis of antibiotic induced gastro-intestinal and mucocutaneous disorders] Acta Med Port. 1981;3(2):143-5.

Padilla-Ruiz M, Fernández Aguiar ME, Arce Nuñez M, Polo Amorín R. [[*Lactobacillus rhamnosus* GG supplementation to reduce side-effects of anti-*Helicobacter pylori* treatment].](http://www.ncbi.nlm.nih.gov/pubmed/23838939) Rev Gastroenterol Peru. 2013;33(2):121-30. In Spanish.

Pozzoni P, Riva A, Bellatorre AG, Amigoni M, Redaelli E, Ronchetti A, et al. *Saccharomyces boulardii* for the prevention of antibiotic-associated diarrhea in adult hospitalized patients: a single-center, randomized, double-blind, placebo-controlled trial. Am J Gasteroenterol. 2012; 107: 922-931. doi: 10.1038/ajg.2012.56

Sampalis J, Psaradellis E, Rampakakis E. Efficacy of BIO K+ CL1285 in the reduction of antibiotic-associated diarrhea – a placebo controlled double-blind randomized, multi-center study. Arch Med Sci. 2010; 6 (1): 56–64. doi: 10.5114/aoms.2010.13508

Seki H, Shiohara M, Matsumura T, Miyagawa N, Tanaka M, Komiyama A, et al. Prevention of antibiotic-associated diarrhea in children by *Clostridium butyricum* MIYAIRI. Pediatrics International. 2003; 45: 86-90.

Shan L, Hou P, Wang Z, Chen N, Shu L, Zhang H, et al. Prevention and treatment of diarrhea with *Saccharomyces boulardii* in children with acute lower respiratory tract infections. Beneficial Microbes. 2013;4(4):329-334. doi: 10.3920/BM2013.0008

Sheu BS, Wu JJ, Lo CY, Wu HW, Chen JH, Lin YS, et al. Impact of supplement with *Lactobacillus*- and *Bifidobacterium* containing yogurt on triple therapy for *Helicobacter pylori* eradication. Aliment Pharmacol Ther. 2002;16:1669-1675.

Sheu BS, Cheng HC, Kao AW, Wang ST, Yang YJ, Yang HB, et al. Pretreatment with *Lactobacillus*- and *Bifidobacterium*-containing yogurt can improve the efficacy of quadruple therapy in eradicating residual *Helicobacter pylori* infection after failed triple therapy. Am J Clin Nutr*.* 2006; 83:864-869. doi: 10.1093/ajcn/83.4.864

[Shimbo I](http://www.ncbi.nlm.nih.gov/pubmed?term=Shimbo%20I%5BAuthor%5D&cauthor=true&cauthor_uid=16437727), [Yamaguchi T](http://www.ncbi.nlm.nih.gov/pubmed?term=Yamaguchi%20T%5BAuthor%5D&cauthor=true&cauthor_uid=16437727), [Odaka T](http://www.ncbi.nlm.nih.gov/pubmed?term=Odaka%20T%5BAuthor%5D&cauthor=true&cauthor_uid=16437727), [Nakajima K](http://www.ncbi.nlm.nih.gov/pubmed?term=Nakajima%20K%5BAuthor%5D&cauthor=true&cauthor_uid=16437727), [Koide A](http://www.ncbi.nlm.nih.gov/pubmed?term=Koide%20A%5BAuthor%5D&cauthor=true&cauthor_uid=16437727), [Koyama H](http://www.ncbi.nlm.nih.gov/pubmed?term=Koyama%20H%5BAuthor%5D&cauthor=true&cauthor_uid=16437727), et al. Effect of *Clostridium butyricum* on fecal flora in *Helicobacter pylori* eradication therapy. [World J Gastroenterol](http://www.ncbi.nlm.nih.gov/pubmed/?term=H+pylori+and+Shimbo+I). 2005;11(47):7520-4.

Song HJ, Kim JY, Jung SA, Kim S, Park H, Jeong Y, et al. Effect of probiotic Lactobacillus (Lacidofil^®^ cap) for the prevention of antibiotic-associated diarrhea: a prospective, randomized, double-blind, multicenter study. J Korean Med Sci. 2010; 25: 1784–91.

Song MJ, Park DI, Park JH, Kim HJ, Cho YK, Sohn CI, et al. The effect of probiotics and mucoprotective agents on PPI-based triple therapy for eradication of *Helicobacter pylori*. Helicobacter. 2010; 15: 206–13. doi: 10.1111/j.1523-5378.2010.00751.x (*S boulardii*)

Surawicz CM, Elmer GW, Speelman P, McFarland LV, Chinn J, van Belle G. Prevention of antibiotic-associated diarrhea by *Saccharomyces boulardii*: a prospective study. Gastroenterol. 1989; 96: 981-8.

Szajewska H, Albrecht P, Topczewska-Cabanek A. Randomized, double-blind, placebo-controlled trial: effect of lactobacillus GG supplementation on *Helicobacter pylori* eradication rates and side effects during treatment in children. J Pediatr Gastroenter Nutr. 2009;48(4):431-6.

Thomas MR, Litin SC, Osmon DR, Corr AP, Weaver AL, Lohse CM. Lack of effect of *Lactobacillus* GG on antibiotic-associated diarrhea: a randomized, placebo-controlled trial. Mayo Clin Proc. 2001; 76(9): 883-889.

Vaisanen ML, Leskinen M, Siitonen A, Kanervo A, Harila-Saari A, Mottonen M, et al. Occurrence of diarrhea in children receiving oral antibiotics with or without probiotic supplementation with *Lactobacillus* GG. Microbiol Ecol Health Dis. 1998; 10: 199-200.

Vanderhoof JA, Whitney DB, Antonson DL, Hanner TL, Lupo JV, Young RJ. Lactobacillus GG in the prevention of antibiotic-associated diarrhea in children. J Pediatrics. 1999;135(5):564-568.

Wang YH, Huang Y. [Effect of *Lactobacillus acidophilus* and *Bifidobacterium bifidum* supplementation to standard triple therapy on *Helicobacter pylori* eradication and dynamic changes in intestinal flora.](http://www.ncbi.nlm.nih.gov/pubmed/24233772) World J Microbiol Biotechnol. 2014;30(3):847-53. doi: 10.1007/s11274-013-1490-2

Wunderlich PF, Braun L, Fumagalli I, D'Apuzzo V, Heim F, Karly M, Lodi R, Politta G, Vonbank F, Zeltner L. Double-blind report on the efficacy of lactic acid-producing *Enterococcus* SF68 in the prevention of antibiotic-associated diarrhoea and in the treatment of acute diarrhoea. J Int Med Res. 1989; 17: 333-8. doi: 10.1177/030006058901700405

Zhang DM, Xu BB, Yu L, Zheng LF, Chen LP, Wang W. [A prospective control study of *Saccharomyces boulardii* in prevention of antibiotic-associated diarrhea in the older inpatients]. Zhonghua Nei Ke Za Zhi. 2017;56(6):398-401. doi: 10.3760/cma.j

Zhang H. [Clinical effect about Boulardii powder combined triple therapy treating *Helicobacter-pylori* infection in children]. Med J Chinese People Health. 2013;25:38-40. In Chinese. [*S. boulardii*]

Zhang Y, Li J. [Effect of *Saccharomyces boulardii* on treatment of *Helicobacter pylori* infection in children of triple therapy]*.* J Clin Pediatr. 2012;30:928-31. *[In Chinese]*

Zhao HM, Ou-Yang HJ, Duan BP, Xu B, Chen ZY, Tang J, et al. [Clinical effect of triple therapy combined with *Saccharomyces boulardii* in the treatment of *Helicobacter pylori* infection in children]. Zhongguo Dang Dai Er Ke Za Zhi*.* 2014; 16(3):230-3. [in Chinese]

Zojaji H, Ghobakhlou M, Rajabalinia H, Aataei E, Sherafat SJ, Moghimi-Dehkordi B, Bahreiny R. The efficacy and safety of adding the probiotic *Saccharomyces boulardii* to standard triple therapy for eradication of *H. pylori*: a randomized controlled trial. Gastroenterol Hepatol From Bed to Bench. 2013;6(Suppl 1): S99-S104.

**Prevention of *C. difficile* Infections (22 RCT, 23 treatment arms)***red indicates CDI was secondary outcome in the trial

Arvola T, Laiho K, Torkkeli S, Mykkanen H, Salminen S, Maunula L, et al. Prophylactic *Lactobacillus* GG reduces antibiotic-associated diarrhea in children with respiratory infections: a randomized study. Pediatrics. 1999; 104 (5):e64.

Beausoleil M, Fortier N, Guénette S, L'ecuyer A, Savoie M, Franco M, et al. Effect of a fermented milk combining *Lactobacillus acidophilus* Cl1285 and *Lactobacillus casei* in the prevention of antibiotic-associated diarrhea: a randomized, double-blind, placebo-controlled trial. Can J Gastroenterol. 2007;21(11):732-6.

Bravo MV, Bunout D, Leiva L, de la Maza MP, Barrera G, de la Maza J, et al. Effect of probiotic *Saccharomyces boulardii* on prevention of AAD in adult outpatients with amoxicillin treatment. Rev Med Chile. 2008:136:981-988.

Can M, Beşirbellioglu BA, Avci IY, Beker CM, Pahsa A. Prophylactic *Saccharomyces boulardii* in the prevention of antibiotic-associated diarrhea: a prospective study. Med Sci Monit. 2006;12(4):19-22.

[Cindoruk M, Erkan G, Karakan T, Dursun A, Unal S.](http://www.ncbi.nlm.nih.gov/pubmed/17669103?ordinalpos=6&itool=EntrezSystem2.PEntrez.Pubmed.Pubmed_ResultsPanel.Pubmed_RVDocSum) Efficacy and safety of *Saccharomyces boulardii* in the 14-day triple anti-*Helicobacter pylori* therapy: a prospective randomized placebo-controlled double-blind study. Helicobacter. 2007;12(4):309-16. doi: 10.1111/j.1523-5378.2007.00516.x

Dietrich CG, Kottmann T, Alavi M. Commercially available probiotic drinks containing *Lactobacillus casei* DN-114001 reduce antibiotic-associated diarrhea. World J Gastrol. 2014:20(42):15837-44. doi: 10.3748/wjg.v20.i42.15837

[Duman DG](http://www.ncbi.nlm.nih.gov/sites/entrez?Db=pubmed&Cmd=Search&Term=%22Duman%20DG%22%5BAuthor%5D&itool=EntrezSystem2.PEntrez.Pubmed.Pubmed_ResultsPanel.Pubmed_DiscoveryPanel.Pubmed_RVAbstractPlus), [Bor S](http://www.ncbi.nlm.nih.gov/sites/entrez?Db=pubmed&Cmd=Search&Term=%22Bor%20S%22%5BAuthor%5D&itool=EntrezSystem2.PEntrez.Pubmed.Pubmed_ResultsPanel.Pubmed_DiscoveryPanel.Pubmed_RVAbstractPlus), [Ozütemiz O](http://www.ncbi.nlm.nih.gov/sites/entrez?Db=pubmed&Cmd=Search&Term=%22Oz%C3%BCtemiz%20O%22%5BAuthor%5D&itool=EntrezSystem2.PEntrez.Pubmed.Pubmed_ResultsPanel.Pubmed_DiscoveryPanel.Pubmed_RVAbstractPlus), [Sahin T](http://www.ncbi.nlm.nih.gov/sites/entrez?Db=pubmed&Cmd=Search&Term=%22Sahin%20T%22%5BAuthor%5D&itool=EntrezSystem2.PEntrez.Pubmed.Pubmed_ResultsPanel.Pubmed_DiscoveryPanel.Pubmed_RVAbstractPlus), [Oğuz D](http://www.ncbi.nlm.nih.gov/sites/entrez?Db=pubmed&Cmd=Search&Term=%22O%C4%9Fuz%20D%22%5BAuthor%5D&itool=EntrezSystem2.PEntrez.Pubmed.Pubmed_ResultsPanel.Pubmed_DiscoveryPanel.Pubmed_RVAbstractPlus), [Iştan F](http://www.ncbi.nlm.nih.gov/sites/entrez?Db=pubmed&Cmd=Search&Term=%22I%C5%9Ftan%20F%22%5BAuthor%5D&itool=EntrezSystem2.PEntrez.Pubmed.Pubmed_ResultsPanel.Pubmed_DiscoveryPanel.Pubmed_RVAbstractPlus), et al. Efficacy and safety of *Saccharomyces boulardii* in prevention of antibiotic-associated diarrhoea due to *Helicobacter pylori* eradication. [Eur J Gastroenterol Hepatol*.*](javascript:AL_get(this,%20'jour',%20'Eur%20J%20Gastroenterol%20Hepatol.');) 2005;17(12):1357-1361.

Ehrhardt S, Guo N, Hinz R, Schoppen S, May J, Reiser M, et al. *Saccharomyces boulardii* to prevent antibiotic-associated diarrhea: A randomized, double-masked, placebo-controlled trial. Open Forum Infect Dis. 2016;29;3(1):1-7. doi: 10.1093/ofid/ofw011

Gao XW, Mubasher M, Fang CY, Reifer C, Miller LE. [Dose-response efficacy of a proprietary probiotic Formula of *Lactobacillus acidophilus* CL1285 and *Lactobacillus casei* LBC80R for antibiotic-associated diarrhea and *Clostridium* *difficile*-associated diarrhea prophylaxis in adult patients.](http://www.ncbi.nlm.nih.gov/pubmed/20145608?itool=EntrezSystem2.PEntrez.Pubmed.Pubmed_ResultsPanel.Pubmed_RVDocSum&ordinalpos=6) Am J Gastroenterol. 2010;105(7):1636-41. doi: 10.1038/ajg.2010.11

[Hickson M, D'Souza AL, Muthu N, Rogers TR, Want S, Rajkumar C, et al](http://www.ncbi.nlm.nih.gov/pubmed/17604300?ordinalpos=3&itool=EntrezSystem2.PEntrez.Pubmed.Pubmed_ResultsPanel.Pubmed_RVDocSum). Use of probiotic *Lactobacillus* preparation to prevent diarrhoea associated with antibiotics: randomised double blind placebo controlled trial. Br Med J 2007;335(7610):80-3. doi: 10.1016/j.jclinepi.2010.09.011

[Kotowska M, Albrecht P, Szajewska H.](http://www.ncbi.nlm.nih.gov/pubmed/15740542?ordinalpos=3&itool=EntrezSystem2.PEntrez.Pubmed.Pubmed_ResultsPanel.Pubmed_RVDocSum) *Saccharomyces boulardii* in the prevention of antibiotic-associated diarrhoea in children: a randomized double-blind placebo-controlled trial. Aliment Pharmacol Ther. 2005;21(5):583-590. doi: 10.1111/j.1365-2036.2005.02356.x

Lewis SJ, Potts LF, Barry RE. The lack of therapeutic effect of *Saccharomyces boulardii* in the prevention of antibiotic-related diarrhoea in elderly patients. J Infect. 1998; 36: 171-4.

McFarland LV, Surawicz CM, Greenberg RN, Elmer GW, Moyer KA, Melcher SA, Bowen KE, Cox JL. Prevention of β-lactam-associated diarrhea by *Saccharomyces boulardii* compared to placebo. Am J Gastroenterol. 1995; 90: 439-48.

Miller M, Florencio S, Eastmond J, Reynolds S. Results of 2 prospective randomized studies of Lactobacillus GG to prevent *C. difficile* infection in hospitalized adults receiving antibiotics. Abstract of the 48th Interscience Conference on Antimicrobial Agents and Chemotherapy, Washington DC 2008A;48:578-579.

Miller M, Florencio S, Eastmond J, Reynolds S. Results of 2 prospective randomized studies of Lactobacillus GG to prevent *C. difficile* infection in hospitalized adults receiving antibiotics. Abstract of the 48th Interscience Conference on Antimicrobial Agents and Chemotherapy, Washington DC 2008B;48:578-579.

Morrow LE, Kollef MH, Casale TB. [Probiotic prophylaxis of ventilator-associated pneumonia: a blinded, randomized, controlled trial.](http://www.ncbi.nlm.nih.gov/pubmed/20522788) Am J Respir Crit Care Med. 2010;182(8):1058-1064. doi: 10.1164/rccm.200912-1853OC

Pozzoni P, Riva A, Bellatorre AG, Amigoni M, Redaelli E, Ronchetti A, et al. *Saccharomyces boulardii* for the prevention of antibiotic-associated diarrhea in adult hospitalized patients: a single-center, randomized, double-blind, placebo-controlled trial. Am J Gasteroenterol. 2012; 107: 922-931. doi: 10.1038/ajg.2012.56

Sampalis J, Psaradellis E, Rampakakis E. Efficacy of BIO K+ CL1285 in the reduction of antibiotic-associated diarrhea – a placebo controlled double-blind randomized, multi-center study. Arch Med Sci. 2010; 6 (1): 56–64. doi: 10.5114/aoms.2010.13508

Shan L, Hou P, Wang Z, Chen N, Shu L, Zhang H, et al. Prevention and treatment of diarrhea with *Saccharomyces boulardii* in children with acute lower respiratory tract infections. Beneficial Microbes. 2013;4(4):329-334. doi: 10.3920/BM2013.0008]

Surawicz CM, Elmer GW, Speelman P, McFarland LV, Chinn J, van Belle G. Prevention of antibiotic-associated diarrhea by *Saccharomyces boulardii*: a prospective study. Gastroenterol. 1989; 96: 981-8.

Thomas MR, Litin SC, Osmon DR, Corr AP, Weaver AL, Lohse CM. Lack of effect of *Lactobacillus* GG on antibiotic-associated diarrhea: a randomized, placebo-controlled trial. Mayo Clin Proc. 2001; 76(9): 883-889.

Zhang DM, Xu BB, Yu L, Zheng LF, Chen LP, Wang W. [A prospective control study of *Saccharomyces boulardii* in prevention of antibiotic-associated diarrhea in the older inpatients]. Zhonghua Nei Ke Za Zhi. 2017;56(6):398-401. doi: 10.3760/cmaj

**Prevention of *H. pylori* eradiation Adverse Reactions (14 RCT, 16 treatment arms)**

*red indicates adverse reactions were secondary outcome in the trial

Armuzzi A, Cremonini F, Ojetti V, Bartolozzi F, Canducci F, Candelli M, et al. Effect of Lactobacillus GG supplementation on antibiotic-associated gastrointestinal side effects during *Helicobacter pylori* eradication therapy: a pilot study. Digestion. 2001**A**;63(1):1-7. doi: [10.1159/000051865](https://doi.org/10.1159/000051865)

Armuzzi A, Cremonini F, Bartolozzi F, Canducci F, Candelli M, Ojetti V, et al. The effect of oral administration of *Lactobacillus* GG on antibiotic-associated gastrointestinal side-effects during *Helicobacter pylori* eradication therapy. Aliment Pharmacol Ther. 2001**B**; 15(2): 163-169. doi: 10. [.1046/j.1365-2036.2001.00923.x](https://doi.org/10.1046/j.1365-2036.2001.00923.x)

Chu Y, Zhu H, Zhou Y, Lv L, Huo J. Intervention study on *Saccharomyces boulardii* with proton pump inhibitor (PPI)-based triple therapy for *Helicobacter pylori* related peptic ulcer. African J Pharmacy Pharmacol. 2012; 6 (41):2900-2904. doi: 10.5897/AJIPP12.400

[Cindoruk M, Erkan G, Karakan T, Dursun A, Unal S.](http://www.ncbi.nlm.nih.gov/pubmed/17669103?ordinalpos=6&itool=EntrezSystem2.PEntrez.Pubmed.Pubmed_ResultsPanel.Pubmed_RVDocSum) Efficacy and safety of *Saccharomyces boulardii* in the 14-day triple anti-*Helicobacter pylori* therapy: a prospective randomized placebo-controlled double-blind study. Helicobacter. 2007;12(4):309-16. doi: 10.1111/j.1523-5378.2007.00516.x

Cremonini F, Di Caro S, Covino M, Armuzzi A, Gabrielli M, Santarelli L, et al. Effect of different probiotic preparations on anti-*Helicobacter pylori* therapy-related side effects: a parallel group, triple blind, placebo-controlled study. Am J Gastroenterol. 2002;97(11):2744-9. doi: 10.1097/MCG.0b013e3182166a42 [2 treatment arms]

[Duman DG](http://www.ncbi.nlm.nih.gov/sites/entrez?Db=pubmed&Cmd=Search&Term=%22Duman%20DG%22%5BAuthor%5D&itool=EntrezSystem2.PEntrez.Pubmed.Pubmed_ResultsPanel.Pubmed_DiscoveryPanel.Pubmed_RVAbstractPlus), [Bor S](http://www.ncbi.nlm.nih.gov/sites/entrez?Db=pubmed&Cmd=Search&Term=%22Bor%20S%22%5BAuthor%5D&itool=EntrezSystem2.PEntrez.Pubmed.Pubmed_ResultsPanel.Pubmed_DiscoveryPanel.Pubmed_RVAbstractPlus), [Ozütemiz O](http://www.ncbi.nlm.nih.gov/sites/entrez?Db=pubmed&Cmd=Search&Term=%22Oz%C3%BCtemiz%20O%22%5BAuthor%5D&itool=EntrezSystem2.PEntrez.Pubmed.Pubmed_ResultsPanel.Pubmed_DiscoveryPanel.Pubmed_RVAbstractPlus), [Sahin T](http://www.ncbi.nlm.nih.gov/sites/entrez?Db=pubmed&Cmd=Search&Term=%22Sahin%20T%22%5BAuthor%5D&itool=EntrezSystem2.PEntrez.Pubmed.Pubmed_ResultsPanel.Pubmed_DiscoveryPanel.Pubmed_RVAbstractPlus), [Oğuz D](http://www.ncbi.nlm.nih.gov/sites/entrez?Db=pubmed&Cmd=Search&Term=%22O%C4%9Fuz%20D%22%5BAuthor%5D&itool=EntrezSystem2.PEntrez.Pubmed.Pubmed_ResultsPanel.Pubmed_DiscoveryPanel.Pubmed_RVAbstractPlus), [Iştan F](http://www.ncbi.nlm.nih.gov/sites/entrez?Db=pubmed&Cmd=Search&Term=%22I%C5%9Ftan%20F%22%5BAuthor%5D&itool=EntrezSystem2.PEntrez.Pubmed.Pubmed_ResultsPanel.Pubmed_DiscoveryPanel.Pubmed_RVAbstractPlus), et al. Efficacy and safety of *Saccharomyces boulardii* in prevention of antibiotic-associated diarrhoea due to *Helicobacter pylori* eradication. [Eur J Gastroenterol Hepatol*.*](javascript:AL_get(this,%20'jour',%20'Eur%20J%20Gastroenterol%20Hepatol.');) 2005;17(12):1357-1361.

Gao C, Xie R, Tianheng MA, Shangnong WU. Therapeutic effect of *Saccharomyces boulardii* combined with standard triple therapy for *Helicobacter pylori* eradication. Chinese J Gastroenterol. 2012;9:555-557.

[Hurduc V, Plesca D, Dragomir D, Sajin M, Vandenplas Y.](http://www.ncbi.nlm.nih.gov/pubmed/18681892?ordinalpos=1&itool=EntrezSystem2.PEntrez.Pubmed.Pubmed_ResultsPanel.Pubmed_RVDocSum) A randomized, open trial evaluating the effect of *Saccharomyces boulardii* on the eradication rate of *Helicobacter* *pylori* infection in children. Acta Paediatr. 2009; 98(1):127-31. doi: 10.1111/j.1651-2227.2008.00977.x

Padilla-Ruiz M, Fernández Aguiar ME, Arce Nuñez M, Polo Amorín R. [[*Lactobacillus rhamnosus* GG supplementation to reduce side-effects of anti-*Helicobacter pylori* treatment].](http://www.ncbi.nlm.nih.gov/pubmed/23838939) Rev Gastroenterol Peru. 2013;33(2):121-30. In Spanish.

Plewinska E, Planeta-Malecka I, Bak-Romaniszyn L, Czkwianianc E, Malecka-Panas E. [Probiotics in the treatment of *Helicobacter pylori* infection in children.] Polish. Gastroenterol Polska 2006;13(4):315-319. [“Lacidophil”= L. helveticus R052 and L. rhamnosus R11]

Song MJ, Park DI, Park JH, Kim HJ, Cho YK, Sohn CI, et al. The effect of probiotics and mucoprotective agents on PPI-based triple therapy for eradication of *Helicobacter pylori*. Helicobacter. 2010; 15: 206–13. doi: 10.1111/j.1523-5378.2010.00751.x (*S boulardii*)

Szajewska H, Albrecht P, Topczewska-Cabanek A. Randomized, double-blind, placebo-controlled trial: effect of lactobacillus GG supplementation on *Helicobacter pylori* eradication rates and side effects during treatment in children. J Pediatr Gastroenterol Nutr**.** 2009;48(4):431-6.

Vdovychenko VL, Demidova AL, Bidyuk OA. [Efficiency of quadrotherapy with probiotics in patients with duodenal peptic ulcer.] Modern Gastroenterol. 2008;5(43);90-92. [Ukranian]

Zhang Y, Li J. Effect of *Saccharomyces boulardii* on treatment of *Helicobacter pylori* infection in children of triple therapy*.* J Clin Pediatr*.* 2012;30:928-31. *[In Chinese]*

**Prevention Nasogastric Tube-Associated Diarrhea (3 RCT)**

Bleichner G, Blehaut H, Mentec H, Moyse D. *Saccharomyces boulardii* prevents diarrhea in critically ill tube-fed patients: A multicenter, randomized, double-blind placebo-controlled trial. Intensive Care Med. 1997; 23: 517-523.

Schlotterer M, Bernasconi P, Lebreton F, Wassermann D. Value of *Saccharomyces boulardii* in the digestive acceptability of continuous-flow enteral nutrition in burnt patients. Nutr Clin Matalbol. 1987; 1: 31-34.

Tempe JD, Steidel AL, Blehaut H, Hasselmann M, Lutun PH, Maurier F. Use of *Saccharomyces boulardii* for the prevention of diarrhea during continuous enteral feeding. Sem Hop Paris. 1983; 59(18): 1409-1412.

**Prevention Necrotizing Enterocolitis (17 RCT)**

[Bin-Nun A, Bromiker R, Wilschanski M, Kaplan M, Rudensky B, Caplan M, Hammerman C.](http://www.ncbi.nlm.nih.gov/pubmed/16126048?ordinalpos=2&itool=EntrezSystem2.PEntrez.Pubmed.Pubmed_ResultsPanel.Pubmed_RVDocSum) Oral probiotics prevent necrotizing enterocolitis in very low birth weight neonates. J Pediatr. 2005 Aug;147(2):192-6.

[Costalos C, Skouteri V, Gounaris A, Sevastiadou S, Triandafilidou A, Ekonomidou C, et al.](http://www.ncbi.nlm.nih.gov/pubmed/14580749?ordinalpos=9&itool=EntrezSystem2.PEntrez.Pubmed.Pubmed_ResultsPanel.Pubmed_RVDocSum)  Enteral feeding of premature infants with *Saccharomyces boulardii*. Early Hum Dev. 2003;74(2):89-96.

[Dani C, Biadaioli R, Bertini G, Martelli E, Rubaltelli FF.](http://www.ncbi.nlm.nih.gov/pubmed/12169832?ordinalpos=87&itool=EntrezSystem2.PEntrez.Pubmed.Pubmed_ResultsPanel.Pubmed_RVDocSum) Probiotics feeding in prevention of urinary tract infection, bacterial sepsis and necrotizing enterocolitis in preterm infants. A prospective double-blind study. Biol Neonate. 2002;82(2):103-8. doi: 10.1159/000063096

Demirel G, Erdeve O, Celik IH, Dilmen U. [*Saccharomyces boulardii* for prevention of necrotizing enterocolitis in preterm infants: a randomized, controlled study.](http://www.ncbi.nlm.nih.gov/pubmed/24028629) Acta Paediatr. 2013;102(12):e560-5. doi: 10.1111/apa.12416

Jacobs SE, Tobin JM, Opie GF, Donath S, Tabrizi SN, Pirotta M, et al: Probiotic effects on late-onset sepsis in very preterm infants: a randomized controlled trial. Pediatrics 2013;132: 1055–1062.

Lin HC, Hsu CH, Chen HL, Chung MY, Hsu JF, Lien RI, Tsao LY, Chen CH, Su BH. Oral probiotics prevent necrotizing enterocolitis in very low birth weight preterm infants: a multicenter, randomized, controlled trial. Pediatrics. 2008 Oct;122(4):693-700. [doi: 10.1542/peds.2007-3007]

[Manzoni P, Mostert M, Leonessa ML, Priolo C, Farina D, Monetti C, et al.](http://www.ncbi.nlm.nih.gov/pubmed/16705580?ordinalpos=18&itool=EntrezSystem2.PEntrez.Pubmed.Pubmed_ResultsPanel.Pubmed_RVDocSum)  Oral supplementation with *Lactobacillus casei* subspecies *rhamnosus* prevents enteric colonization by Candida species in preterm neonates: a randomized study. Clin Infect Dis. 2006;42(12):1735-42. doi: 10.1086/504324

Manzoni P, Rinaldi M, Cattani S, Pugni L, Romeo MG, Messner H, Stolfi I, Decembrino L, Laforgia N, Vagnarelli F, Memo L, Bordignon L, Saia OS, Maule M, Gallo E, Mostert M, Magnani C, Quercia M, Bollani L, Pedicino R, Renzullo L, Betta P, Mosca F, Ferrari F, Magaldi R, Stronati M, Farina D; Italian Task Force for the Study and Prevention of Neonatal Fungal Infections, Italian Society of Neonatology. Bovine lactoferrin supplementation for prevention of late-onset sepsis in very low-birth-weight neonates: a randomized trial. JAMA. 2009 Oct 7;302(13):1421-8. [doi: 10.1001/jama.2009.1403. PMID: 19809023]

Manzoni P, Meyer M, Stolfi I, Rinaldi M, Cattani S, Pugni L, Romeo MG, Messner H, Decembrino L, Laforgia N, Vagnarelli F, Memo L, Bordignon L, Maule M, Gallo E, Mostert M, Quercia M, Bollani L, Pedicino R, Renzullo L, Betta P, Ferrari F, Alexander T, Magaldi R, Farina D, Mosca F, Stronati M. Bovine lactoferrin supplementation for prevention of necrotizing enterocolitis in very-low-birth-weight neonates: a randomized clinical trial. Early Hum Dev. 2014 Mar;90 Suppl 1:S60-5. [doi: 10.1016/S0378-3782(14)70020-9. PMID: 24709463]

[Mihatsch WA](https://www.ncbi.nlm.nih.gov/pubmed/?term=Mihatsch%20WA%5BAuthor%5D&cauthor=true&cauthor_uid=20234140), [Vossbeck S](https://www.ncbi.nlm.nih.gov/pubmed/?term=Vossbeck%20S%5BAuthor%5D&cauthor=true&cauthor_uid=20234140), [Eikmanns B](https://www.ncbi.nlm.nih.gov/pubmed/?term=Eikmanns%20B%5BAuthor%5D&cauthor=true&cauthor_uid=20234140), [Hoegel J](https://www.ncbi.nlm.nih.gov/pubmed/?term=Hoegel%20J%5BAuthor%5D&cauthor=true&cauthor_uid=20234140), [Pohlandt F](https://www.ncbi.nlm.nih.gov/pubmed/?term=Pohlandt%20F%5BAuthor%5D&cauthor=true&cauthor_uid=20234140). Effect of *Bifidobacterium lactis* on the incidence of nosocomial infections in very-low-birth-weight infants: a randomized controlled trial. [Neonatology.](https://www.ncbi.nlm.nih.gov/pubmed/?term=Mihatsch+WA+and+Bifidobacterium) 2010;98(2):156-63. doi: 10.1159/000280291

[Mohan R, Koebnick C, Schildt J, Schmidt S, Mueller M, Possner M, et al.](http://www.ncbi.nlm.nih.gov/pubmed/16971641?ordinalpos=2&itool=EntrezSystem2.PEntrez.Pubmed.Pubmed_ResultsPanel.Pubmed_RVDocSum)  Effects of *Bifidobacterium lactis* Bb12 supplementation on intestinal microbiota of preterm infants: a double-blind, placebo-controlled, randomized study. J Clin Microbiol. 2006;44(11):4025-31. doi:10.1128/JCM.00767-06

Nouri -Shadkam M, Jalalizadeh F, Nasiriani K. Effects of probiotic lactobacillus reuteri (DSM 17938) on the incidence of necrotizing enterocolitis in very low birth weight premature infants. Iranian Journal of Neonatology IJN. 2015;6(4):15-20.

Oncel MY, Sari FN, Arayici S, Guzoglu N, Erdeve O, Uras N, et al. *Lactobacillus reuteri* for the prevention of necrotising enterocolitis in very low birthweight infants: a randomised controlled trial. Arch Dis Child Fetal Neonatal Ed. 2014;99(2): F110-5. doi: 10.1136/archdischild-2013-304745

Oncel MY, Arayici S, Sari FN, Simsek GK, Yurttutan S, Erdeve O, et al. [Comparison of *Lactobacillus reuteri* and nystatin prophylaxis on Candida colonization and infection in very low birth weight infants.](https://www.ncbi.nlm.nih.gov/pubmed/25245226) J Matern Fetal Neonatal Med. 2015;28(15):1790-4. doi: 10.3109/14767058.2014.968842

[Rojas MA](https://www.ncbi.nlm.nih.gov/pubmed/?term=Rojas%20MA%5BAuthor%5D&cauthor=true&cauthor_uid=23071204), [Lozano JM](https://www.ncbi.nlm.nih.gov/pubmed/?term=Lozano%20JM%5BAuthor%5D&cauthor=true&cauthor_uid=23071204), [Rojas MX](https://www.ncbi.nlm.nih.gov/pubmed/?term=Rojas%20MX%5BAuthor%5D&cauthor=true&cauthor_uid=23071204), [Rodriguez VA](https://www.ncbi.nlm.nih.gov/pubmed/?term=Rodriguez%20VA%5BAuthor%5D&cauthor=true&cauthor_uid=23071204), [Rondon MA](https://www.ncbi.nlm.nih.gov/pubmed/?term=Rondon%20MA%5BAuthor%5D&cauthor=true&cauthor_uid=23071204), [Bastidas JA](https://www.ncbi.nlm.nih.gov/pubmed/?term=Bastidas%20JA%5BAuthor%5D&cauthor=true&cauthor_uid=23071204), et al. Prophylactic probiotics to prevent death and nosocomial infection in preterm infants. [Pediatrics.](https://www.ncbi.nlm.nih.gov/pubmed/?term=Rojas+MA+and+L.+reuteri) 2012;130(5):e1113-20. doi: 10.1542/peds.2011-3584. [*L. reuteri* DSM17938]

Saengtawesin V, Tangpolkaiwalsak R, Kanjanapattankul W: Effect of oral probiotics supplementation in the prevention of necrotizing enterocolitis among very low birth weight preterm infants. J Med Assoc Thai 2014; 97:S20–S25.[*L. acidophilus*+ *Bifido. bifidum]*

Serce O, Benzer D, Gursoy T, Karatekin G, Ovali F. [Efficacy of *Saccharomyces boulardii* on necrotizing enterocolitis or sepsis in very low birth weight infants: a randomised controlled trial.](http://www.ncbi.nlm.nih.gov/pubmed/24041815) Early Hum Dev. 2013;89(12):1033-6. doi: 10.1016/j.earlhumdev.2013.08.013

**Prevention Nosocomial Infections (2 RCT)**

Hojsak I, Abdovic S, Szajewska H, Milosevic M, Krznaric Z, Kolacek S. Lactobacillus GG in the prevention of nosocomial gastrointestinal and respiratory tract infections: a randomized, placebo-controlled, double-blind study. Pediatrics. 2010;125(5): e1171-e1176. doi: 10.1542/peds, PMID:20403940

Honeycutt TC, El Khashab M, Wardrop RM 3rd, McNeal-Trice K, Honeycutt AL, Christy CG, et al. Probiotic administration and the incidence of nosocomial infection in pediatric intensive care: a randomized placebo-controlled trial. Pediatr Crit Care Med. 2007;8(5):452-8. doi: [10.1097/01.PCC.0000282176.41134.E6](https://doi.org/10.1097/01.PCC.0000282176.41134.E6)

**Prevention Respiratory Tract Infections (10 RCT)**

*red indicates respiratory tract infections were a secondary outcome

Boge T, Rémigy M, Vaudaine S, Tanguy J, Bourdet-Sicard R, van der Werf S. A probiotic fermented dairy drink improves antibody response to influenza vaccination in the elderly in two randomised controlled trials. Vaccine. 2009;27(41):5677-84. doi: 10.1016/j.vaccine.2009.06.094

Giovannone M, Barberani F, Boschetto S, Gigliozzi A, Tosoni M. *Lactobacillus casei* DG effectiveness on *Helicobacter pylori* eradication treatment-side effectis; a placebo-controlled, double-blind randomized pilot study. Gastroenterol. 2007;132 (Suppl 2):A614. Meeting Abstract. Presented at Amer Gastroenterol Association Meeting, May 19-24, 2007, Washington DC.

Guillemard E, Tondu F, Lacoin F, Schrezenmeir J. Consumption of a fermented dairy product containing the probiotic *Lactobacillus casei* DN-114 001 reduces the duration of respiratory infection in the elderly in a randomised controlled trial. Br J Nutr. 2010; 103:58-68. doi: 10.1017/S0007114509991395

Hatakka K, Savilahti E, Pönkä A, Meurman JH, Poussa T, Näse L, et al. Effect of long term consumption of probiotic milk on infections in children attending day care centres: double blind, randomised trial. BMJ. 2001;322(7298):1327. .

Hojsak I, Abdovic S, Szajewska H, Milosevic M, Krznaric Z, Kolacek S. *Lactobacillus* GG in the prevention of nosocomial gastrointestinal and respiratory tract infections: a randomized, placebo-controlled, double-blind study. Pediatrics**.** 2010B;125(5):e1171-e1176. doi: 10.1542/peds

Honeycutt TC, El Khashab M, Wardrop RM 3rd, McNeal-Trice K, Honeycutt AL, Christy CG, et al. Probiotic administration and the incidence of nosocomial infection in pediatric intensive care: a randomized placebo-controlled trial. Pediatr Crit Care Med. 2007;8(5):452-8. doi: [10.1097/01.PCC.0000282176.41134.E6](https://doi.org/10.1097/01.PCC.0000282176.41134.E6)

[Kumpu M](http://www.ncbi.nlm.nih.gov/pubmed/?term=Kumpu%20M%5BAuthor%5D&cauthor=true&cauthor_uid=26322544), Kekkonen RA, Korpela R, Tynkkynen S, Järvenpää S, Kautiainen H, et al. Effect of live and inactivated *Lactobacillus rhamnosus* GG on experimentally induced rhinovirus colds: randomised, double blind, placebo-controlled pilot trial. Beneficial Microbes. 2015;6(5):631-9. doi: 10.3920/BM2014.0164

Luoto R, Ruuskanen O, Waris M, Kalliomäki M, Salminen S, Isolauri E. Prebiotic and probiotic supplementation prevents rhinovirus infections in preterm infants: a randomized, placebo-controlled trial. Allergy Clin Immunol. 2014;133(2):405-13. doi: 10.1016/j.jaci.2013.08.020

Morrow LE, Kollef MH, Casale TB. [Probiotic prophylaxis of ventilator-associated pneumonia: a blinded, randomized, controlled trial.](http://www.ncbi.nlm.nih.gov/pubmed/20522788) Am J Respir Crit Care Med. 2010;182(8):1058-1064. doi: 10.1164/rccm.200912-1853OC

Turchet P, [Laurenzano M](https://www.ncbi.nlm.nih.gov/pubmed/?term=Laurenzano%20M%5BAuthor%5D&cauthor=true&cauthor_uid=12679825), [Auboiron S](https://www.ncbi.nlm.nih.gov/pubmed/?term=Auboiron%20S%5BAuthor%5D&cauthor=true&cauthor_uid=12679825), [Antoine JM](https://www.ncbi.nlm.nih.gov/pubmed/?term=Antoine%20JM%5BAuthor%5D&cauthor=true&cauthor_uid=12679825). Effect of fermented milk containing the probiotic *Lactobacillus casei* DN-114001 on winter infections in free-living elderly subjects: a randomised, controlled pilot study. [J Nutr Health Aging.](https://www.ncbi.nlm.nih.gov/pubmed/?term=Turchet+and+Lactobacillus) 2003;7(2):75-7.

**Prevention Post-Surgical Infections (8 RCT)**

Kotzampassi K, Giamarellos-Bourboulis EJ, Voudouris A, Kazamias P, Eleftheriadis E. Benefits of a Synbiotic Formula (Synbiotic 2000Forte^®^) in Critically Ill Trauma Patients: Early Results of a Randomized Controlled Trial. [World Journal of Surgery](https://link.springer.com/journal/268) 2006; 30(10):1848-1855.

McNaught CE, Woodcock NP, MacFie J, Mitchell CJ. A prospective randomised study of the probiotic *Lactobacillus plantarum* 299V on indices of gut barrier function in elective surgical patients. Gut. 2002;51(6):827-31.

Rayes N, Hansen S, Seehofer D, Müller AR, Serke S, Bengmark S, et al. Early enteral supply of fiber and Lactobacilli versus conventional nutrition: A controlled trial in patients with major abdominal surgery. Nutrition. 2002A; 18:609–615.

Rayes N, Seehofer D, Hansen S, Boucsein K, Müller AR, Serke S, et al. Early enteral supply of Lactobacillus and fiber versus selective bowel decontamination: a controlled trial in liver transplant patients. Transplantation 2002B;74(1): 123-128.

Rayes N, Seehofer D, Theruvath T, Schiller RA, Langrehr JM, Jonas S, Bengmark S, Neuhaus P. [Supply of pre- and probiotics reduces bacterial infection rates after liver transplantation--a randomized, double-blind trial.](https://www.ncbi.nlm.nih.gov/pubmed/15636620) Am J Transplant. 2005 Jan;5(1):125-.

Rayes N, Seehofer D, Theruvath T, Mogl M, Langrehr JM, Nüssler NC, Bengmark S, Neuhaus P. E[ffect of enteral nutrition and synbiotics on bacterial infection rates after pylorus-preserving pancreatoduodenectomy: a randomized, double-blind trial.](https://www.ncbi.nlm.nih.gov/pubmed/17592288) Ann Surg. 2007 Jul;246(1):36-41.

Rayes N, Pilarski T, Stockmann M, Bengmark S, Neuhaus P, Seehofer D. E[ffect of pre- and probiotics on liver regeneration after resection: a randomised, double-blind pilot study.](https://www.ncbi.nlm.nih.gov/pubmed/22968413) Benef Microbes. 2012 Sep;3(3):237-44. [doi: 10.3920/BM2012.0006]

[Spindler-Vesel A](http://www.ncbi.nlm.nih.gov/sites/entrez?Db=pubmed&Cmd=Search&Term=%22Spindler-Vesel%20A%22%5BAuthor%5D&itool=EntrezSystem2.PEntrez.Pubmed.Pubmed_ResultsPanel.Pubmed_DiscoveryPanel.Pubmed_RVAbstractPlus), [Bengmark S](http://www.ncbi.nlm.nih.gov/sites/entrez?Db=pubmed&Cmd=Search&Term=%22Bengmark%20S%22%5BAuthor%5D&itool=EntrezSystem2.PEntrez.Pubmed.Pubmed_ResultsPanel.Pubmed_DiscoveryPanel.Pubmed_RVAbstractPlus), [Vovk I](http://www.ncbi.nlm.nih.gov/sites/entrez?Db=pubmed&Cmd=Search&Term=%22Vovk%20I%22%5BAuthor%5D&itool=EntrezSystem2.PEntrez.Pubmed.Pubmed_ResultsPanel.Pubmed_DiscoveryPanel.Pubmed_RVAbstractPlus), [Cerovic O](http://www.ncbi.nlm.nih.gov/sites/entrez?Db=pubmed&Cmd=Search&Term=%22Cerovic%20O%22%5BAuthor%5D&itool=EntrezSystem2.PEntrez.Pubmed.Pubmed_ResultsPanel.Pubmed_DiscoveryPanel.Pubmed_RVAbstractPlus), [Kompan L](http://www.ncbi.nlm.nih.gov/sites/entrez?Db=pubmed&Cmd=Search&Term=%22Kompan%20L%22%5BAuthor%5D&itool=EntrezSystem2.PEntrez.Pubmed.Pubmed_ResultsPanel.Pubmed_DiscoveryPanel.Pubmed_RVAbstractPlus). Synbiotics, prebiotics, glutamine, or peptide in early enteral nutrition: a randomized study in trauma patients.  [J Parenter Enteral Nutr.](javascript:AL_get(this,%20'jour',%20'JPEN%20J%20Parenter%20Enteral%20Nutr.');) 2007 Mar-Apr;31(2):119-26.

**Prevention of Traveler’s diarrhea (5 RCT, 7 treatment arms)**

Bruns R and Raedsch R. Therapy of traveller’s diarrhea. Medizinische Welt. 1995;46:591-596.

Hilton E, Kolakowski P, Singer C, Smith M. Efficacy of *Lactobacillus GG* as a diarrheal preventive in travelers. J Travel Med. 1997; 4(1):41-43.

Kollaritsch H, Kremsner P, Wiedermann, G, Scheiner O. Prevention of traveller’s diarrhea: comparison of different non-antibiotic preparations. Trav Med Internatl 1989;7:9-18. (2 txt arms)

Kollaritsch H, Holst H, Grobara P, Wiedermann G. Prophylaxe der reisediarrhoe mit *Saccharomyces boulardii*. [Prevention of traveler's diarrhea with *Saccharomyces boulardii*. Results of a placebo controlled double-blind study]. Fortschr Med. 1993; 111(9):152-156. (2 txt arms)

Oksanen PJ, Salminen S, Saxelin M, Hamalainen P, Ihantola-Vormisto A, Muurasniemi-Isoviita L et al. Prevention of travellers' diarrhoea by *Lactobacillus GG*. Ann Med. 1990; 22(1):53-56.

**Prevention Urinary Tract Infections** **(3 RCT)**

*red indicates UTI was a secondary outcome

[Dani C, Biadaioli R, Bertini G, Martelli E, Rubaltelli FF.](http://www.ncbi.nlm.nih.gov/pubmed/12169832?ordinalpos=87&itool=EntrezSystem2.PEntrez.Pubmed.Pubmed_ResultsPanel.Pubmed_RVDocSum) Probiotics feeding in prevention of urinary tract infection, bacterial sepsis and necrotizing enterocolitis in preterm infants. A prospective double-blind study. Biol Neonate. 2002;82(2):103-8. doi: 10.1159/000063096

Honeycutt TC, El Khashab M, Wardrop RM 3rd, McNeal-Trice K, Honeycutt AL, Christy CG, et al. Probiotic administration and the incidence of nosocomial infection in pediatric intensive care: a randomized placebo-controlled trial. Pediatr Crit Care Med. 2007;8(5):452-8. doi: [10.1097/01.PCC.0000282176.41134.E6](https://doi.org/10.1097/01.PCC.0000282176.41134.E6)

Kontiokari T, Sundqvist K, Nuutinen M, Pokka T, Koskela M, Uhari M. Randomised trial of cranberry-lingonberry juice and Lactobacillus GG drink for the prevention of urinary tract infections in women. BMJ. 2001;322(7302):1571.

**Treatment of Adult Acute Diarrhea (9 RCT)**

*red indicates adult diarrhea was a secondary outcome

Attar A, Flourié B, Rambaud JC, Franchisseur C, Ruszniewski P, Bouhnik Y. Antibiotic efficacy in small intestinal bacterial overgrowth-related chronic diarrhea: a crossover, randomized trial. Gastroenterology. 1999;117(4):794-7.

[Besirbellioglu BA, Ulcay A, Can M, Erdem H, Tanyuksel M, Avci IY, et al.](http://www.ncbi.nlm.nih.gov/pubmed/16798698?ordinalpos=25&itool=EntrezSystem2.PEntrez.Pubmed.Pubmed_ResultsPanel.Pubmed_RVDocSum)  *Saccharomyces boulardii* and infection due to *Giardia lamblia*. Scand J Infect Dis. 2006;38(6-7):479-81.

Buydens P, Debeuckelaere S. Efficacy of SF68 in the treatment of acute diarrhea. A placebo-controlled trial. Scand J Gastroenterol. 1996; 31: 887-91.

Heo J, Kim SK, Park KS, Jung HK, Kwon JG, Jang BI. A double-blind, randomized, active drug comparative, parallel-group, multi-center clinical study to evaluate the safety and efficacy of probiotics (*Bacillus licheniformis*, Zhengchangsheng ® capsule) in patients with diarrhea. Intestinal Research. 2014;12(3):236-244. doi: 10.5217/ir.2014.12.3.236 [*S boulardii* was the comparison group]

Hochter W, Chase D, Hagenhoff G. *Saccharomyces boulardii* in acute adult diarrhea: efficacy and tolerability of treatment. Munch Med Wschr. 1990; 132: 188-192.

Mansour-Ghanael F, Dehbashi N, Yazdanparast K, Shafaghi A. Efficacy of *Saccharomyces boulardii* with antibiotics in acute amoebiasis. World J Gastroenterol. 2003;9:1832-1833.

Mitra AK, Rabbani GH. A double-blind, controlled trial of Bioflorin (*Streptococcus faecium* SF68) in adults with acute diarrhea due to *Vibrio cholerae* and enterotoxigenic *Escherichia coli*. Gastroenterol**.** 1990; 99: 1149-52.

Saint-Marc Th, Blehaut H, Musial Ch, Touraine JL. AIDS-related diarrhea: a double-blind trial of *Saccharomyces boulardii*. Sem Hôp Paris. 1995; 71: 735-741.

Wunderlich PF, Braun L, Fumagalli I, D'Apuzzo V, Heim F, Karly M, et al. Double-blind report on the efficacy of lactic acid-producing *Enterococcus* SF68 in the prevention of antibiotic-associated diarrhoea and in the treatment of acute diarrhoea. J Int Med Res. 1989; 17: 333-8. doi: 10.1177/030006058901700405

**Treatment *C. difficile* infections (4 RCT)**

Lawrence SJ, Korzenik JR, Mundy LM. Probiotics for recurrent *Clostridium difficile* disease. J Med Microbiol. 2005;54(Pt 9):905-6. doi: 10.1099/jmm.0.46096-0

McFarland LV, Surawicz CM, Greenberg RN, Fekety R, Elmer GW, Moyer KA, et al. A randomized placebo-controlled trial of *Saccharomyces boulardii* in combination with standard antibiotics for *Clostridium difficile* disease. JAMA. 1994; 271: 1913-8.

Pochapin M. The effect of probiotics on *Clostridium difficile* diarrhea. Am J Gastroenterol. 2000; 95: S11-3.

Surawicz CM, McFarland LV, Greenberg RN, Rubin M, Fekety R, Mulligan ME, et al. The search for a better treatment for recurrent *Clostridium difficile* disease: use of high-dose vancomycin combined with *Saccharomyces boulardii*. Clin Infect Dis. 2000**;** 31(4):1012-1017. doi: 10.1086/318130

**Treatment of Infantile Colic (4 RCT)**

Indrio F, Di Mauro A, Riezzo G, Civardi E, Intini C, Corvaglia L, et al. Prophylactic use of a probiotic in the prevention of colic, regurgitation, and functional constipation: A randomized clinical trial. JAMA Pediatr. 2014;168(3):228-33. doi: 10.1001/jamapediatrics.2013.4367

Savino F, Pelle E, Palumeri E, Oggero R, Miniero R. *Lactobacillus reuteri* (American Type Culture Collection Strain 55730) versus simethicone in the treatment of infantile colic: a prospective randomized study. Pediatrics. 2007;119(1):e124-30. doi: 10.1542/peds.2006-1222

Savino F, Cordisco L, Tarasco V, Palumeri E, Calabrese R, Oggero R, et al. *Lactobacillus reuteri* DSM 17938 in infantile colic: a randomized, double-blind, placebo-controlled trial. Pediatrics. 2010;126(3):e526-33. doi: 10.1542/peds.2010-0433

Szajewska H, Gyrczuk E, Horvath A. *Lactobacillus reuteri* DSM 17938 for the management of infantile colic in breastfed infants: a randomized, double-blind, placebo-controlled trial. J Pediatr. 2013;162(2):257-62. doi: 10.1016/j.jpeds.2012.08.004

**Treatment of Constipation (3 RCT)**

[De Paula JA](http://www.ncbi.nlm.nih.gov/sites/entrez?Db=pubmed&Cmd=Search&Term=%22De%20Paula%20JA%22%5BAuthor%5D&itool=EntrezSystem2.PEntrez.Pubmed.Pubmed_ResultsPanel.Pubmed_DiscoveryPanel.Pubmed_RVAbstractPlus), [Carmuega E](http://www.ncbi.nlm.nih.gov/sites/entrez?Db=pubmed&Cmd=Search&Term=%22Carmuega%20E%22%5BAuthor%5D&itool=EntrezSystem2.PEntrez.Pubmed.Pubmed_ResultsPanel.Pubmed_DiscoveryPanel.Pubmed_RVAbstractPlus), [Weill R](http://www.ncbi.nlm.nih.gov/sites/entrez?Db=pubmed&Cmd=Search&Term=%22Weill%20R%22%5BAuthor%5D&itool=EntrezSystem2.PEntrez.Pubmed.Pubmed_ResultsPanel.Pubmed_DiscoveryPanel.Pubmed_RVAbstractPlus). Effect of the ingestion of a symbiotic yogurt on the bowel habits of women with functional constipation. [Acta Gastroenterol Latinoam](javascript:AL_get(this,%20'jour',%20'Acta%20Gastroenterol%20Latinoam.');). 2008;38(1):16-25.

Tabbers MM, Chmielewska A, Roseboom MG, Crastes N, Perrin C, Reitsma JB, et al. Fermented milk containing *Bifidobacterium [animalis] lactis* DN-173 010 in childhood constipation: a randomized, double-blind, controlled trial. Pediatrics. 2011; 127(6):e1392-9. doi: 10.1542/peds.2010-2590

Yang YX, He M, Hu G, Wei J, Pages P, Yang XH, et al. Effect of a fermented milk containing *Bifidobacterium lactis (animalis*) DN-173 010 on Chinese constipated women. World J Gastroenterol. 2008; 14(40):6237-43.

**Treatment *H. pylori* eradication (33 RCT, 35 treatment arms)**

Abaturov OE, Gerasymenko ON. Efficacy of *H. pylori* eradication in children with chronic gastroduodenitis who receive combined antibacterial and probiotic therapy. Contemporary Pediatrics. 2014; 2(58):90-94.

Armuzzi A, Cremonini F, Ojetti V, Bartolozzi F, Canducci F, Candelli M, et al. Effect of Lactobacillus GG supplementation on antibiotic-associated gastrointestinal side effects during *Helicobacter pylori* eradication therapy: a pilot study. Digestion. 2001**A**;63(1):1-7. doi: [10.1159/000051865](https://doi.org/10.1159/000051865)

Armuzzi A, Cremonini F, Bartolozzi F, Canducci F, Candelli M, Ojetti V, et al. The effect of oral administration of *Lactobacillus* GG on antibiotic-associated gastrointestinal side-effects during *Helicobacter pylori* eradication therapy. Aliment Pharmacol Ther. 2001**B**; 15(2): 163-169. doi: 10. [.1046/j.1365-2036.2001.00923.x](https://doi.org/10.1046/j.1365-2036.2001.00923.x)

Babak OY, Malaya LT. The use of Lacidofil in treatment of duodenal peptic ulcers associated with *H. pylori*. [*News of Pharmacy and Medicine*]. 2007;5:24-25. Ukranian.

[Bin Z](http://www.ncbi.nlm.nih.gov/pubmed/?term=Bin%20Z%5BAuthor%5D&cauthor=true&cauthor_uid=25866729), [Ya-Zheng X](http://www.ncbi.nlm.nih.gov/pubmed/?term=Ya-Zheng%20X%5BAuthor%5D&cauthor=true&cauthor_uid=25866729), [Zhao-Hui D](http://www.ncbi.nlm.nih.gov/pubmed/?term=Zhao-Hui%20D%5BAuthor%5D&cauthor=true&cauthor_uid=25866729), [Bo C](http://www.ncbi.nlm.nih.gov/pubmed/?term=Bo%20C%5BAuthor%5D&cauthor=true&cauthor_uid=25866729), [Li-Rong J](http://www.ncbi.nlm.nih.gov/pubmed/?term=Li-Rong%20J%5BAuthor%5D&cauthor=true&cauthor_uid=25866729), [Vandenplas Y](http://www.ncbi.nlm.nih.gov/pubmed/?term=Vandenplas%20Y%5BAuthor%5D&cauthor=true&cauthor_uid=25866729). The Efficacy of *Saccharomyces boulardii* CNCM I-745 in addition to standard *Helicobacter pylori* eradication treatment in children. Pediatr Gastroenterol Hepatol Nutr. 2015;18(1):17-22. doi: 10.5223/pghn.2015.18.1.17

Canducci F, Armuzzi A, Cremonini F, Cammarota G, Bartolozzi F, Pola P, et al. A lyophilized and inactivated culture of *Lactobacillus acidophilus* increases *Helicobacter pylori* eradication rates. Aliment Pharmacol Ther. 2000;14(12):1625-9.

Chu Y, Zhu H, Zhou Y, Lv L, Huo J. Intervention study on *Saccharomyces boulardii* with proton pump inhibitor (PPI)-based triple therapy for *Helicobacter pylori* related peptic ulcer. African J Pharmacy Pharmacol. 2012; 6 (41):2900-2904. doi: 10.5897/AJIPP12.400

[Cindoruk M, Erkan G, Karakan T, Dursun A, Unal S.](http://www.ncbi.nlm.nih.gov/pubmed/17669103?ordinalpos=6&itool=EntrezSystem2.PEntrez.Pubmed.Pubmed_ResultsPanel.Pubmed_RVDocSum) Efficacy and safety of *Saccharomyces boulardii* in the 14-day triple anti-*Helicobacter pylori* therapy: a prospective randomized placebo-controlled double-blind study. Helicobacter. 2007;12(4):309-16. doi: 10.1111/j.1523-5378.2007.00516.x

Cremonini F, Di Caro S, Covino M, Armuzzi A, Gabrielli M, Santarelli L, et al. Effect of different probiotic preparations on anti-*Helicobacter pylori* therapy-related side effects: a parallel group, triple blind, placebo-controlled study. Am J Gastroenterol. 2002;97(11):2744-9. doi: 10.1097/MCG.0b013e3182166a42 [2 treatment arms Sb and Lr GG]

De Francesco V, Stoppino V, Sgarro C, Faleo D. [*Lactobacillus acidophilus* administration added to omeprazole/amoxycillin-based double therapy in *Helicobacter pylori* eradication.](http://www.ncbi.nlm.nih.gov/pubmed/11142590) Dig Liver Dis. 2000;32(8):746-7.

De Vrese M, Kristen H, Rautenberg P, Laue C, Schrezenmei J. Probiotic lactobacilli and bifidobacteria in a fermented milk product with added fruit preparation reduce antibiotic associated diarrhea and *Helicobacter pylori* activity. J Dairy Res. 2011; 78(4):396-403. doi: 10.1017/S002202991100063X

Gao C, Xie R, Tianheng MA, Shangnong WU. Therapeutic effect of *Saccharomyces boulardii* combined with standard triple therapy for *Helicobacter pylori* eradication. Chinese J Gastroenterol. 2012;9:555-557.

[Gotteland M, Poliak L, Cruchet S, Brunser O.](http://www.ncbi.nlm.nih.gov/pubmed/16421034?ordinalpos=10&itool=EntrezSystem2.PEntrez.Pubmed.Pubmed_ResultsPanel.Pubmed_RVDocSum) Effect of regular ingestion of *Saccharomyces boulardii* plus inulin or *Lactobacillus acidophilus* LB in children colonized by *Helicobacter pylori.* Acta Paediatr. 2005;94(12):1747-51. doi: 10.1080/08035250500252120 (2 treatment arms Sb and La LB]

Guo JB, Yang PF, Wang MT, Lu GY, et al. [The application of *Clostridium* to the eradication of *Helicobacter pylori]*. Chin J Celiopathy. 2004;3(4):163-5. Chinese. (*Clostridium butyricum 588*)

[Hurduc V, Plesca D, Dragomir D, Sajin M, Vandenplas Y.](http://www.ncbi.nlm.nih.gov/pubmed/18681892?ordinalpos=1&itool=EntrezSystem2.PEntrez.Pubmed.Pubmed_ResultsPanel.Pubmed_RVDocSum) A randomized, open trial evaluating the effect of *Saccharomyces boulardii* on the eradication rate of *Helicobacter* *pylori* infection in children. Acta Paediatr. 2009; 98(1):127-31. doi: 10.1111/j.1651-2227.2008.00977.x

[Imase K, Takahashi M, Tanaka A, Tokunaga K, Sugano H, Tanaka M, et al.](http://www.ncbi.nlm.nih.gov/pubmed/18402597?ordinalpos=1&itool=EntrezSystem2.PEntrez.Pubmed.Pubmed_ResultsPanel.Pubmed_RVDocSum) Efficacy of *Clostridium butyricum* preparation concomitantly with *Helicobacter pylori* eradication therapy in relation to changes in the intestinal microbiota. Microbiol Immunol. 2008;52(3):156-61. doi:10.1111/j.1348-0421.2008.00026.x

Kyriakos N, Papmichael K, Roussos A, Therodoropoulos I, Karakoidas C, Smyrnidis A, et al. Lyophilized form of *Saccharomyces boulardii* enhances the *Helicobacter pylori* eradication rates of omeprazole-triple therapy in patients with peptic ulcer disease or functional dyspepsia. Hosp Chronicles. 2013; 8(3):127-133.

Lee JY, Shoi SP, Park JH, Shin JW, Joo YH. The effect of *Saccharomyces boulardii* as an adjuvant to the 14-day triple therapy for eradication of *Helicobacter pylori*. Abstract #44. Presented at Asian Pacific Digestive Week, 1-4 October 2011, SUNTEC Singapore. *J Gastro & Hepatol*. 2011;26(S5):257.

Mirzaee V, Rezahosseini O. Randomized control trial: Comparison of triple therapy plus probiotic yogurt vs. standard triple therapy on *Helicobacter pylori* eradication. Iran Red Crescent Med J. 2012;14(10):657-66.

Ozdil K, Calhan T, Sahin A, Senates E, Kahraman R, Yüzbasioglu B, et al. Levofloxacin based sequential and triple therapy compared with standard plus probiotic combination for *Helicobacter pylori* eradication. Hepatogastroenterol. 2011;58(109):1148-52. doi: 10.5754/hge11075

Plewinska E, Planeta-Malecka I, Bak-Romaniszyn L, Czkwianianc E, Malecka-Panas E. [Probiotics in the treatment of *Helicobacter pylori* infection in children.] Gastroenterol Polska. 2006;13(4):315-319. Polish. Abstract in English. [“Lacidophil”= *L. helveticus* R052 and *L. rhamnosus* R11)

Sheu BS, Wu JJ, Lo CY, Wu HW, Chen JH, Lin YS, et al. Impact of supplement with *Lactobacillus*- and *Bifidobacterium* containing yogurt on triple therapy for *Helicobacter pylori* eradication. Aliment Pharmacol Ther. 2002;16:1669-1675.

Sheu BS, Cheng HC, Kao AW, Wang ST, Yang YJ, Yang HB, et al. Pretreatment with *Lactobacillus*- and *Bifidobacterium*-containing yogurt can improve the efficacy of quadruple therapy in eradicating residual *Helicobacter pylori* infection after failed triple therapy. Am J Clin Nutr*.* 2006; 83:864-869. doi: 10.1093/ajcn/83.4.864

[Shimbo I](http://www.ncbi.nlm.nih.gov/pubmed?term=Shimbo%20I%5BAuthor%5D&cauthor=true&cauthor_uid=16437727), [Yamaguchi T](http://www.ncbi.nlm.nih.gov/pubmed?term=Yamaguchi%20T%5BAuthor%5D&cauthor=true&cauthor_uid=16437727), [Odaka T](http://www.ncbi.nlm.nih.gov/pubmed?term=Odaka%20T%5BAuthor%5D&cauthor=true&cauthor_uid=16437727), [Nakajima K](http://www.ncbi.nlm.nih.gov/pubmed?term=Nakajima%20K%5BAuthor%5D&cauthor=true&cauthor_uid=16437727), [Koide A](http://www.ncbi.nlm.nih.gov/pubmed?term=Koide%20A%5BAuthor%5D&cauthor=true&cauthor_uid=16437727), [Koyama H](http://www.ncbi.nlm.nih.gov/pubmed?term=Koyama%20H%5BAuthor%5D&cauthor=true&cauthor_uid=16437727), et al. Effect of *Clostridium butyricum* on fecal flora in *Helicobacter pylori* eradication therapy. [World J Gastroenterol](http://www.ncbi.nlm.nih.gov/pubmed/?term=H+pylori+and+Shimbo+I). 2005;11(47):7520-4.

Song MJ, Park DI, Park JH, Kim HJ, Cho YK, Sohn CI, et al. The effect of probiotics and mucoprotective agents on PPI-based triple therapy for eradication of *Helicobacter pylori*. Helicobacter. 2010; 15: 206–13. doi: 10.1111/j.1523-5378.2010.00751.x (*S boulardii*)

Szajewska H, Albrecht P, Topczewska-Cabanek A. Randomized, double-blind, placebo-controlled trial: effect of *Lactobacillus* GG supplementation on *Helicobacter pylori* eradication rates and side effects during treatment in children. J Pediatr Gastroenterol Nutr 2009;48(4):431-6.

Vdovychenko VL, Demidova AL, Bidyuk OA. [Efficiency of quadrotherapy with probiotics in patients with duodenal peptic ulcer.] Modern Gastroentero.] Ukranrian 2008;5(43);90-92.

Wang YH, Huang Y. [Effect of *Lactobacillus acidophilus* and *Bifidobacterium bifidum* supplementation to standard triple therapy on *Helicobacter pylori* eradication and dynamic changes in intestinal flora.](http://www.ncbi.nlm.nih.gov/pubmed/24233772) World J Microbiol Biotechnol. 2014;30(3):847-53. doi: 10.1007/s11274-013-1490-2

Zhang H. [Clinical effect of Boulardii powder combined with triple therapy treatment of *Helicobacter-pylori* infection in children. Med J Chinese People Health. In Chinese. 2013;25:38-40. [*S. boulardii*]

Zhang Y, Li J. [Effect of *Saccharomyces boulardii* on treatment of *Helicobacter pylori* infection in children of triple therapy]*.* J Clin Pediatr. 2012;30:928-31. [In Chinese]

Zhao HM, Ou-Yang HJ, Duan BP, Xu B, Chen ZY, Tang J, et al. [Clinical effect of triple therapy combined with *Saccharomyces boulardii* in the treatment of *Helicobacter pylori* infection in children]. Zhongguo Dang Dai Er Ke Za Zhi*.* 2014; 16(3):230-3. [In Chinese]

Ziemniak W. Efficacy of *Helicobacter pylori* eradication taking into account its resistance to antibiotics. J Physiol Pharmacol. 2006;57:123-141.

Zojaji H, Ghobakhlou M, Rajabalinia H, Aataei E, Sherafat SJ, Moghimi-Dehkordi B, Bahreiny R. The efficacy and safety of adding the probiotic *Saccharomyces boulardii* to standard triple therapy for eradication of *H. pylori*: a randomized controlled trial. Gastroenterol Hepatol From Bed to Bench. 2013;6(Suppl 1): S99-S104.

**Treament of Inflammatory Bowel Disease (25 RCT)**

[Bourreille A](http://www.ncbi.nlm.nih.gov/pubmed/?term=Bourreille%20A%5BAuthor%5D&cauthor=true&cauthor_uid=23466709), [Cadiot G](http://www.ncbi.nlm.nih.gov/pubmed/?term=Cadiot%20G%5BAuthor%5D&cauthor=true&cauthor_uid=23466709), [Le Dreau G](http://www.ncbi.nlm.nih.gov/pubmed/?term=Le%20Dreau%20G%5BAuthor%5D&cauthor=true&cauthor_uid=23466709), [Laharie D](http://www.ncbi.nlm.nih.gov/pubmed/?term=Laharie%20D%5BAuthor%5D&cauthor=true&cauthor_uid=23466709), [Beaugerie L](http://www.ncbi.nlm.nih.gov/pubmed/?term=Beaugerie%20L%5BAuthor%5D&cauthor=true&cauthor_uid=23466709), [Dupas JL](http://www.ncbi.nlm.nih.gov/pubmed/?term=Dupas%20JL%5BAuthor%5D&cauthor=true&cauthor_uid=23466709), et al. *Saccharomyces boulardii* does not prevent relapse of Crohn's disease. [Clin Gastroenterol Hepatol.](http://www.ncbi.nlm.nih.gov/pubmed/?term=Bourreille+and+S.+boulardii) 2013;11(8):982-7. doi: 10.1016/j.cgh.2013.02.021

Bousvaros A, Guandalini S, Baldassano RN, Botelho C, Evans J, Ferry GD, et al. A randomized, double-blind trial of *Lactobacillus* GG versus placebo in addition to standard maintenance therapy for children with Crohn's disease. Inflamm Bowel Dis. 2005;11(9): 833-839.

Campieri M, Rizzello F, Venture A, Poggioli G, Ugolini F, Helwig U, et al. Combination of antibiotic and probiotic treatment is efficacious in prophylaxis of post-operative recurrence of Crohn's disease: a randomized controlled study vs mesalamine. Gastroenterol. 2000;118:A4179.

Gionchetti P, Rizzello F, Venturi A, Brigidi P, Matteuzzi D, Bazzocchi G, et al. Oral bacteriotherapy as maintenance treatment in patients with chronic pouchitis: a double-blind, placebo-controlled trial. Gastroenterol. 2000;119(2):305-9.

Gionchetti P, Rizzello F, Helwig U, Venturi A, Lammers KM, Brigidi P, et al. Prophylaxis of pouchitis onset with probiotic therapy: A double-blind, placebo-controlled trial. Gastroenterol. 2003; 124: 1202-9.

[Gosselink MP, Schouten WR, van Lieshout LM, Hop WC, Laman JD, Ruseler-van Embden JG.](http://www.ncbi.nlm.nih.gov/pubmed/15108026?ordinalpos=22&itool=EntrezSystem2.PEntrez.Pubmed.Pubmed_ResultsPanel.Pubmed_RVDocSum) Delay of the first onset of pouchitis by oral intake of the probiotic strain *Lactobacillus rhamnosus* GG. Dis Colon Rectum. 2004;47(6):876-84. doi: 10.1007/s10350-004-0525-z

Guslandi M, Mezzi G, Sorghi M, Testoni PA. *Saccharomyces boulardii* in maintenance treatment of Crohn's disease. Dig Dis Sci. 2000;45, 1462-1464.

[Kuisma J](https://www.ncbi.nlm.nih.gov/pubmed/?term=Kuisma%20J%5BAuthor%5D&cauthor=true&cauthor_uid=12622759), [Mentula S](https://www.ncbi.nlm.nih.gov/pubmed/?term=Mentula%20S%5BAuthor%5D&cauthor=true&cauthor_uid=12622759), [Jarvinen H](https://www.ncbi.nlm.nih.gov/pubmed/?term=Jarvinen%20H%5BAuthor%5D&cauthor=true&cauthor_uid=12622759), [Kahri A](https://www.ncbi.nlm.nih.gov/pubmed/?term=Kahri%20A%5BAuthor%5D&cauthor=true&cauthor_uid=12622759), [Saxelin M](https://www.ncbi.nlm.nih.gov/pubmed/?term=Saxelin%20M%5BAuthor%5D&cauthor=true&cauthor_uid=12622759), [Farkkila M](https://www.ncbi.nlm.nih.gov/pubmed/?term=Farkkila%20M%5BAuthor%5D&cauthor=true&cauthor_uid=12622759). Effect of *Lactobacillus rhamnosus* GG on ileal pouch inflammation and microbial flora. Aliment Pharmacol Ther. 2003;17(4):509-15.

Kruis W. Schutz E, Fric P, Fixa B, Judmaier G, Stolte M. Double blind comparison of an oral *Escherichia coli* preparation and mesalazine in maintaining remission of ulcerative colitis. Aliment Pharmacol Ther. 1997;11, 853-858.

Kruis W, Fric P, Pokrotnieks J, Lukas M, Fixa B, Kascak M, et al. Maintaining remission of ulcerative colitis with the probiotic *Escherichia coli* Nissle 1917 is as effective as with standard mesalazine. Gut. 2004;53, 1617-1623. doi: 10.1136/gut.2003.037747

Kuhbacher T, Ott SJ, Helwig U, Mimura T, Rizzello F, Kleessen B, et al. Bacterial and fungal microbiota in relation to probiotic therapy (VSL#3) in pouchitis. Gut. 2006;55:833-41. doi: 10.1136/gut.2005.078303

Malchow HA. Crohn's disease and *Escherichia coli*. A new approach in therapy to maintain remission of colonic Crohn's disease? J Clin Gastroenterol. 1997;25:653-658.

Matthes H, [Krummenerl T](https://www.ncbi.nlm.nih.gov/pubmed/?term=Krummenerl%20T%5BAuthor%5D&cauthor=true&cauthor_uid=20398311), [Giensch M](https://www.ncbi.nlm.nih.gov/pubmed/?term=Giensch%20M%5BAuthor%5D&cauthor=true&cauthor_uid=20398311), [Wolff C](https://www.ncbi.nlm.nih.gov/pubmed/?term=Wolff%20C%5BAuthor%5D&cauthor=true&cauthor_uid=20398311), [Schulze J](https://www.ncbi.nlm.nih.gov/pubmed/?term=Schulze%20J%5BAuthor%5D&cauthor=true&cauthor_uid=20398311). Clinical trial: probiotic treatment of acute distal ulcerative colitis with rectally administered *Escherichia coli* Nissle 1917 (EcN). BMC Complement Altern Med. 2010;10:13. doi: 10.1186/1472-6882-10-13

Miele E, [Pascarella F](https://www.ncbi.nlm.nih.gov/pubmed/?term=Pascarella%20F%5BAuthor%5D&cauthor=true&cauthor_uid=19174792), [Giannetti E](https://www.ncbi.nlm.nih.gov/pubmed/?term=Giannetti%20E%5BAuthor%5D&cauthor=true&cauthor_uid=19174792), [Quaglietta L](https://www.ncbi.nlm.nih.gov/pubmed/?term=Quaglietta%20L%5BAuthor%5D&cauthor=true&cauthor_uid=19174792), [Baldassano RN](https://www.ncbi.nlm.nih.gov/pubmed/?term=Baldassano%20RN%5BAuthor%5D&cauthor=true&cauthor_uid=19174792), [Staiano A](https://www.ncbi.nlm.nih.gov/pubmed/?term=Staiano%20A%5BAuthor%5D&cauthor=true&cauthor_uid=19174792). Effect of a probiotic preparation (VSL#3) on induction and maintenance of remission in children with ulcerative colitis. Am J Gastroenterol. 2009;104(2):437-43. doi: 10.1038/ajg.2008.118

Mimura T, Rizzello F, Helwig U, Poggioli G, Schreiber S, Talbot IC, et al. Once daily high dose probiotic therapy (VSL#3) for maintaining remission in recurrent or refractory pouchitis. Gut. 2004;53:108-114.

Plein K, Hotz J. Therapeutic effects of *Saccharomyces boulardii* on mild residual symptoms in a stable phase of Crohn's disease with special respect to chronic diarrhea--a pilot study. Z Gastroenterol. 1993;31, 129-134.

Prantera C, Scribano ML, Falasco G, Andreoli A, Luzi C. Ineffectiveness of probiotics in preventing recurrence after curative resection for Crohn's disease: a randomised controlled trial with *Lactobacillus* GG. Gut. 2002;51, 405-409.

Rembacken, B.J., Snelling, A.M., Hawkey, P.M., Chalmers, D.M. and Axon, A.T. Non-pathogenic Escherichia coli versus mesalazine for the treatment of ulcerative colitis: a randomised trial. *Lancet* 1999;354, 635-639.

Rizzello F, Gionchetti P, Venturi A, Amadini C, Morselli C, Ugolini F, et al. Prophylaxis of postoperative recurrence of Crohn's disease: a combination of antibiotic and probiotic versus mesalazine. Dig Liv Dis. 2000;32, A37.

Schultz M, Timmer A, Herfarth HH, Sartor RB, Vanderhoof JA, Rath HC. *Lactobacillus* GG in inducing and maintaining remission of Crohn's disease. BMC Gastroenterol. 2004; 4: 5. doi: [10.1186/1471-230X-4-5](https://doi.org/10.1186/1471-230X-4-5)

Sood A, [Midha V](https://www.ncbi.nlm.nih.gov/pubmed/?term=Midha%20V%5BAuthor%5D&cauthor=true&cauthor_uid=19631292), [Makharia GK](https://www.ncbi.nlm.nih.gov/pubmed/?term=Makharia%20GK%5BAuthor%5D&cauthor=true&cauthor_uid=19631292), [Ahuja V](https://www.ncbi.nlm.nih.gov/pubmed/?term=Ahuja%20V%5BAuthor%5D&cauthor=true&cauthor_uid=19631292), [Singal D](https://www.ncbi.nlm.nih.gov/pubmed/?term=Singal%20D%5BAuthor%5D&cauthor=true&cauthor_uid=19631292), [Goswami P](https://www.ncbi.nlm.nih.gov/pubmed/?term=Goswami%20P%5BAuthor%5D&cauthor=true&cauthor_uid=19631292), et al. The probiotic preparation, VSL#3 induces remission in patients with mild-to-moderately active ulcerative colitis. Clin Gastroenterol Hepatol. 2009;7(11):1202-9, 1209.e1. doi: 10.1016/j.cgh.2009.07.016.

Tursi A, Brandimarte G, Giorgetti GM, Forti G, Modeo ME, Gigliobianco A. Low-dose balsalazide plus a high-potency probiotic preparation is more effective than balsalazide alone or mesalazine in the treatment of acute mild-to moderate ulcerative colitis. Med Sci Monitor. 2004;10:I126-131.

Tursi A, Brandimarte G, Papa A, Giglio A, Elisei W, Giorgetti GM, et al. Treatment of relapsing mild-to-moderate ulcerative colitis with the probiotic VSL#3 as adjunctive to a standard pharmaceutical treatment: a double-blind, randomized, placebo-controlled study. Am J Gastroenterol. 2010;105(10):2218-27. doi: 10.1038/ajg.2010.218

Zocco MA, Zileri Dal Verme L, Armuzzi A, Nista EC, Papa A, et al. Comparison of *Lactobacillus GG* and mesalazine in maintaining remission of ulcerative colitis and Crohn's disease. Abstract from Digestive Disease Week, May 17-27, 2003 Abstract #S1352. Gastroenterol. 2003;124(4), Suppl 1, A201.

Zocco MA, dal Verme LZ, Cremonini F, Piscaglia AC, Nista EC, Candelli M, et al. Efficacy of *Lactobacillus* GG in maintaining remission of ulcerative colitis. Aliment Pharmacol Ther. 2006;23(11):1567-74. doi: 10.1111/j.1365-2036.2006.02927.x

**Treatment of Irritable Bowel Syndrome (21 RCT)**

[Abbas Z](http://www.ncbi.nlm.nih.gov/pubmed/?term=Abbas%20Z%5BAuthor%5D&cauthor=true&cauthor_uid=24722560), [Yakoob J](http://www.ncbi.nlm.nih.gov/pubmed/?term=Yakoob%20J%5BAuthor%5D&cauthor=true&cauthor_uid=24722560), [Jafri W](http://www.ncbi.nlm.nih.gov/pubmed/?term=Jafri%20W%5BAuthor%5D&cauthor=true&cauthor_uid=24722560), [Ahmad Z](http://www.ncbi.nlm.nih.gov/pubmed/?term=Ahmad%20Z%5BAuthor%5D&cauthor=true&cauthor_uid=24722560), [Azam Z](http://www.ncbi.nlm.nih.gov/pubmed/?term=Azam%20Z%5BAuthor%5D&cauthor=true&cauthor_uid=24722560), [Usman MW](http://www.ncbi.nlm.nih.gov/pubmed/?term=Usman%20MW%5BAuthor%5D&cauthor=true&cauthor_uid=24722560), et al. Cytokine and clinical response to *Saccharomyces boulardii* therapy in diarrhea-dominant irritable bowel syndrome: a randomized trial. Eur J Gastroenterol Hepatol. 2014;26(6):630-9. doi: 10.1097/MEG.0000000000000094

Agrawal A, Houghton LA, Morris J, Reilly B, Guyonnet D, Feuillerat NG, et al. Clinical trial: the effects of a fermented milk product containing *Bifidobacterium lactis* DN-173010 on abdominal distension and gastrointestinal transit in irritable bowel syndrome with constipation. Alim Pharmacol Therap. 2009; 29:104-114. doi: 10.1111/j.1365-2036.2008.03853.x

Bausserman M, Michail S. The use of *Lactobacillus* GG in irritable bowel syndrome in children: a double-blind randomized control trial. J Pediatr. 2005;147(2):197-201. doi: 10.1016/j.jpeds.2005.05.015

[Choi CH](http://www.ncbi.nlm.nih.gov/pubmed/?term=Choi%20CH%5BAuthor%5D&cauthor=true&cauthor_uid=21301358), [Jo SY](http://www.ncbi.nlm.nih.gov/pubmed/?term=Jo%20SY%5BAuthor%5D&cauthor=true&cauthor_uid=21301358), [Park HJ](http://www.ncbi.nlm.nih.gov/pubmed/?term=Park%20HJ%5BAuthor%5D&cauthor=true&cauthor_uid=21301358), [Chang SK](http://www.ncbi.nlm.nih.gov/pubmed/?term=Chang%20SK%5BAuthor%5D&cauthor=true&cauthor_uid=21301358), [Byeon JS](http://www.ncbi.nlm.nih.gov/pubmed/?term=Byeon%20JS%5BAuthor%5D&cauthor=true&cauthor_uid=21301358), [Myung SJ](http://www.ncbi.nlm.nih.gov/pubmed/?term=Myung%20SJ%5BAuthor%5D&cauthor=true&cauthor_uid=21301358). A randomized, double-blind, placebo-controlled multicenter trial *of Saccharomyces boulardii* in irritable bowel syndrome: effect on quality of life. [J Clin Gastroenterol.](http://www.ncbi.nlm.nih.gov/pubmed/?term=Choi+CH+and+S.+boulardii) 2011;45(8):679-83. doi: 10.1097/MCG.0b013e318204593e

Ducrotté P, Sawant P, Jayanthi V. Clinical trial: *Lactobacillus plantarum* 299v (DSM 9843) improves symptoms of irritable bowel syndrome. World J Gastroenterol. 2012;18(30):4012-8. doi: 10.3748/wjg.v18.i30.4012

Francavilla R, Miniello V, Magistà AM, De Canio A, Bucci N, Gagliardi F, et al. [A randomized controlled trial of *Lactobacillus* GG in children with functional abdominal pain.](https://www.ncbi.nlm.nih.gov/pubmed/21078735) Pediatrics. 2010;126(6):e1445-52. doi: 10.1542/peds.2010-0467

Gawrońska A, Dziechciarz P, Horvath A, Szajewska H. [A randomized double-blind placebo-controlled trial of *Lactobacillus* GG for abdominal pain disorders in children.](http://www.ncbi.nlm.nih.gov/pubmed/17229242) Aliment Pharmacol Ther. 2007;25(2):177-84. doi: 10.1111/j.1365-2036.2006.03175.x

Guandalini S, Magazzù G, Chiaro A, La Balestra V, Di Nardo G, Gopalan S, et al. VSL#3 improves symptoms in children with irritable bowel syndrome: a multicenter, randomized, placebo-controlled, double-blind, crossover study. J Pediatr Gastroenterol Nutr. 2010;51(1):24-30. doi: 10.1097/MPG.0b013e3181ca4d95

[Guyonnet D, Chassany O, Ducrotte P, Picard C, Mouret M, Mercier CH, et al.](http://www.ncbi.nlm.nih.gov/pubmed/17635382?ordinalpos=5&itool=EntrezSystem2.PEntrez.Pubmed.Pubmed_ResultsPanel.Pubmed_RVDocSum)  Effect of a fermented milk containing *Bifidobacterium animalis* DN-173 010 on the health-related quality of life and symptoms in irritable bowel syndrome in adults in primary care: a multicentre, randomized, double-blind, controlled trial. Aliment Pharmacol Ther. 2007;26(3):475-86. doi: 10.1111/j.1365-2036.2007.03362.x

[Kabir MA](http://www.ncbi.nlm.nih.gov/pubmed/?term=Kabir%20MA%5BAuthor%5D&cauthor=true&cauthor_uid=21804501), [Ishaque SM](http://www.ncbi.nlm.nih.gov/pubmed/?term=Ishaque%20SM%5BAuthor%5D&cauthor=true&cauthor_uid=21804501), [Ali MS](http://www.ncbi.nlm.nih.gov/pubmed/?term=Ali%20MS%5BAuthor%5D&cauthor=true&cauthor_uid=21804501), [Mahmuduzzaman M](http://www.ncbi.nlm.nih.gov/pubmed/?term=Mahmuduzzaman%20M%5BAuthor%5D&cauthor=true&cauthor_uid=21804501), [Hasan M](http://www.ncbi.nlm.nih.gov/pubmed/?term=Hasan%20M%5BAuthor%5D&cauthor=true&cauthor_uid=21804501). Role of *Saccharomyces boulardii* in diarrhea predominant irritable bowel syndrome. [Mymensingh Med J*.*](http://www.ncbi.nlm.nih.gov/pubmed/?term=Kabir+MA+and+S.+boulardii) 2011;20(3):397-401.

Kim HJ, Camilleri M, McKinzie S, Lempke MB, Burton DD, Thomforde GH, et al. A randomized controlled trial of a probiotic, VSL#3, on gut transit and symptoms in diarrhoea-predominant irritable bowel syndrome. Aliment Pharmacol Ther. 2003;17:895-904.

Kim HJ, Vazquez Roque MI, Camilleri M, Stephens D, Burton DD, Baxter K, et al. A randomized controlled trial of a probiotic combination VSL# 3 and placebo in irritable bowel syndrome with bloating. Neurogastroenterol Motil*.* 2005;17(5):687-696. doi: 10.1111/j.1365-2982.2005.00695.x

Maupas JL, Champemont P, Delforge M. Treatment of irritable bowel syndrome. Double blind trial of *Saccharomyces boulardii*. Medecine Chirurgie Digestives. 1983;12(1):77-79.

[Michail S](https://www.ncbi.nlm.nih.gov/pubmed/?term=Michail%20S%5BAuthor%5D&cauthor=true&cauthor_uid=22247743), [Kenche H](https://www.ncbi.nlm.nih.gov/pubmed/?term=Kenche%20H%5BAuthor%5D&cauthor=true&cauthor_uid=22247743). Gut microbiota is not modified by randomized, double-blind, placebo-controlled trial of VSL#3 in diarrhea-predominant irritable bowel dyndrome. Probiotics Antimicrob Proteins. 2011;3(1):1-7. doi: 10.1007/s12602-010-9059-y

Niedzielin K, Kordecki H, Birkenfeld B. A controlled, double-blind, randomized study on the efficacy of *Lactobacillus plantarum* 299V in patients with irritable bowel syndrome. Eur J Gastroenterol Hepatol. 2001;13(10):1143-1147.

Nobaek S, Johansson ML, Molin G, Ahrne S, Jeppsson B. Alteration of intestinal microflora is associated with reduction in abdominal bloating and pain in patients with irritable bowel syndrome. *Am J Gastroenterol* May 2000;95(5):1231-1238. doi: 10.1111/j.1572-0241.2000.02015.x

O'Mahony L, McCarthy J, Kelly P, Hurley G, Luo F, Chen K, et al. Lactobacillus and Bifidobacterium in irritable bowel syndrome: symptom responses and relationship to cytokine profiles. Gastroenterology*.* 2005;128(3):541-551.

O’Sullivan MA, O’Morain CA. Bacterial supplementation in the irritable bowel syndrome. A randomized double-blind placebo-controlled crossover study. Digest Liver Dis. 2000;32(4):294-301.

Sawant PD, Venkatraman J, Ducrotte PR. Evaluation of *L. plantarum* 299v efficacy in IBS: results of a randomized placebo-controlled trial in 200 patients. Presented at Dig Dis Weekly Meeting, May 7-10, 2010 Chicago, Ill.

Simren M, Syrous A, Lindh A, Abrahamsson H. Effects of *Lactobacillus plantarum* 299v on symptoms and rectal sensitivity in patients with irritable bowel syndrome (IBS) - a randomized, double-blind controlled trial. Gastroenterol. 2006; 130(4): A600.

Whorwell PJ, Altringer L, Morel J, Bond Y, Charbonneau D, O'Mahony L, et al. Efficacy of an encapsulated probiotic *Bifidobacterium infantis* 35624 in women with irritable bowel syndrome. Am J Gastroenterol. 2006;101(7):1581-1590. doi: 10.1111/j.1572-0241.2006.00734.x

**Treatment Pediatric Acute Diarrhea (59 RCT, 61 treatment arms)**

Agrawal KN, Bhasin SK, Faridi MA, Mathur M, Gupta S. *Lactobacillus casei* in the control of acute diarrhea-a pilot study. Indian Pediatrics. 2001; 38:905-910.

Agarwal KN, Bhasin SK. Feasibility studies to control acute diarrhea in children by feeding fermented milk preparations Actimel and Indian Dahi. Euro J Clin Nutr. 2002; 56(S4):S56-S59. doi: 10.1038/sj.ejcn.1601664

Basu S, Chatterjee M, Ganguly S, Chandra PK. Efficacy of *Lactobacillus rhamnosus* GG in acute watery diarrhoea of Indian children: a randomized controlled trial. J Paediatr Child Health. 2007; 43: 837–42. doi: 10.1111/j.1440-1754.2007.01201.x

Billoo AG, Memon MA, Khaskheli SA, Murtaza G, Iqbal K, Saeed Shekhani M, et al. Role of probiotic *Saccharomyces boulardii* in management and prevention of diarrhea. World J Gastroenterol. 2006;12(28):4557-4560.

Boulloche J, Mouterde O, Mallet E. Management of acute diarrhea in infants and toddlers: controlled study of the antidiarrheal efficacy of killed *Lactobacillus acidophilus* (LB strain) versus placebo and a reference agent (loperamide). Ann Pediatr. 1994; 41: 457-63.

Burande MA. Comparison of efficacy of *Saccharomyces boulardii* strain in the treatment of acute diarrhea in children: A prospective, single blind, randomized controlled clinical trial. [J Pharmacol Pharmacother](http://www.ncbi.nlm.nih.gov/pubmed/23960427). 2013; 4(3):205-8. doi: [10.4103/0976-500X.114603](https://doi.org/10.4103/0976-500X.114603)

[Canani RB, Cirillo P, Terrin G, Cesarano L, Spagnuolo MI, De Vincenzo A, et al.](http://www.ncbi.nlm.nih.gov/pubmed/17690340?ordinalpos=3&itool=EntrezSystem2.PEntrez.Pubmed.Pubmed_ResultsPanel.Pubmed_RVDocSum)  Probiotics for treatment of acute diarrhoea in children: randomised clinical trial of five different preparations. BMJ. 2007;335(7615):340. doi: 10.1136/bmj.39272.581736.55 (Four treatment arms: *Bacillus clausii O/C84, N/R84, T84,SIN84*, *L. rhamnosus* GG, *S. boulardii I-745*, *Enterococcus faecium* SF68 and *L. acid* nr+*Bifid. bifidum* nr +2 starters)

Cetina-Sauri G, Basto GS. Evaluacion terapeutica de *Saccharomyces boulardii* en ninos con diarrhea aguda. [Therapeutic evaluation of *Saccharomyces boulardii* in children with acute diarrhea] Tribuna Medica. 1989;56:111-115.

Chapoy P. Treatment of acute diarrhea in infants: a controlled trial of *Saccharomyces boulardii.*  Ann Pediatr (Paris). 1985; 32: 561-563.

Chouraqui JP, Dietsch C, Musial H, Blehaut H. *Saccharomyces boulardii* in the management of toddler diarrhea: a double blind-placebo controlled study. J. Pediatr. Gastroenterol. Nutr. 1995; 20: 463. Meeting Abstract #71.

[Corrêa NB](http://www.ncbi.nlm.nih.gov/pubmed/?term=Corr%C3%AAa%20NB%5BAuthor%5D&cauthor=true&cauthor_uid=21734600), [Penna FJ](http://www.ncbi.nlm.nih.gov/pubmed/?term=Penna%20FJ%5BAuthor%5D&cauthor=true&cauthor_uid=21734600), [Lima FM](http://www.ncbi.nlm.nih.gov/pubmed/?term=Lima%20FM%5BAuthor%5D&cauthor=true&cauthor_uid=21734600), [Nicoli JR](http://www.ncbi.nlm.nih.gov/pubmed/?term=Nicoli%20JR%5BAuthor%5D&cauthor=true&cauthor_uid=21734600), [Filho LA](http://www.ncbi.nlm.nih.gov/pubmed/?term=Filho%20LA%5BAuthor%5D&cauthor=true&cauthor_uid=21734600). Treatment of acute diarrhea with *Saccharomyces boulardii* in infants. [J Pediatr Gastroenterol Nutr.](http://www.ncbi.nlm.nih.gov/pubmed/?term=correa+NB+and+saccharomyces) 2011;53(5):497-501. doi: 10.1097/MPG.0b013e31822b7ab0

Costa-Ribeiro H, Ribeiro TCM, Mattos AP, Valois SS, Neri DA, Almeida P, et al. Limitations of probiotic therapy in acute, severe dehydrating diarrhea. J Pediatric Gastroenterol Nutr. 2003; 36: 112-115.

Dalgic N, Sancar M, Bayraktar B, Pullu M, Hasim O. [Probiotic, zinc and lactose-free formula in children with rotavirus diarrhea: are they effective?](http://www.ncbi.nlm.nih.gov/pubmed/21261786) Pediatr Int. 2011;53(5):677-82. doi: 10.1111/j.1442-200X.2011.03325.x

Dinleyici EC, Eren M, Yargic ZA, Dogan N, Vandenplas Y. Clinical efficacy of *Saccharomyces boulardii* and metronidazole compared to metronidazole alone in children with acute bloody diarrhea caused by amebiasis: a prospective, randomized, open label study. Am J Trop Med Hyg**.** 2009;80(6):953-5.

Dinleyici EC, Eren M, Dogan N, Reyhanioglu S, Yargic ZA, Vandenplas Y. [Clinical efficacy of *Saccharomyces boulardii* or metronidazole in symptomatic children with *Blastocystis hominis* infection.](http://www.ncbi.nlm.nih.gov/pubmed/20922415) Parasitol Res**.** 2011;108(3):541-5. doi: 10.1007/s00436-010-2095-4

Dinleyici EC, Vandenplas Y, PROBAGE Study Group. [*Lactobacillus reuteri* DSM 17938 effectively reduces the duration of acute diarrhoea in hospitalised children.](https://www.ncbi.nlm.nih.gov/pubmed/24579935) Acta Paediatr. 2014;103(7):e300-5. doi: 10.1111/apa.12617

Dinleyici EC, Kara A, Dalgic N, Kurugol Z, Arica V, Metin O, et al. *Saccharomyces boulardii* CNCM I-745 reduces the duration of diarrhoea, length of emergency care and hospital stay in children with acute diarrhoea. Benef Microbes. 2015A**;** 6(4):415-21. doi: 10.3920/BM2014.0086

[Dinleyici EC](https://www.ncbi.nlm.nih.gov/pubmed/?term=Dinleyici%20EC%5BAuthor%5D&cauthor=true&cauthor_uid=25986615), [Dalgic N](https://www.ncbi.nlm.nih.gov/pubmed/?term=Dalgic%20N%5BAuthor%5D&cauthor=true&cauthor_uid=25986615), [Guven S](https://www.ncbi.nlm.nih.gov/pubmed/?term=Guven%20S%5BAuthor%5D&cauthor=true&cauthor_uid=25986615), [Metin O](https://www.ncbi.nlm.nih.gov/pubmed/?term=Metin%20O%5BAuthor%5D&cauthor=true&cauthor_uid=25986615), [Yasa O](https://www.ncbi.nlm.nih.gov/pubmed/?term=Yasa%20O%5BAuthor%5D&cauthor=true&cauthor_uid=25986615), [Kurugol Z](https://www.ncbi.nlm.nih.gov/pubmed/?term=Kurugol%20Z%5BAuthor%5D&cauthor=true&cauthor_uid=25986615), et al. *Lactobacillus reuteri* DSM 17938 shortens acute infectious diarrhea in a pediatric outpatient setting. [J Pediatr (Rio J)](https://www.ncbi.nlm.nih.gov/pubmed/25986615). 2015B;91(4):392-6. doi: 10.1016/j.jped.2014.10.009

[Erdoğan O](http://www.ncbi.nlm.nih.gov/pubmed/?term=Erdo%C4%9Fan%20O%5BAuthor%5D&cauthor=true&cauthor_uid=22778754), [Tanyeri B](http://www.ncbi.nlm.nih.gov/pubmed/?term=Tanyeri%20B%5BAuthor%5D&cauthor=true&cauthor_uid=22778754), [Torun E](http://www.ncbi.nlm.nih.gov/pubmed/?term=Torun%20E%5BAuthor%5D&cauthor=true&cauthor_uid=22778754), [Gönüllü E](http://www.ncbi.nlm.nih.gov/pubmed/?term=G%C3%B6n%C3%BCll%C3%BC%20E%5BAuthor%5D&cauthor=true&cauthor_uid=22778754), [Arslan H](http://www.ncbi.nlm.nih.gov/pubmed/?term=Arslan%20H%5BAuthor%5D&cauthor=true&cauthor_uid=22778754), [Erenberk U](http://www.ncbi.nlm.nih.gov/pubmed/?term=Erenberk%20U%5BAuthor%5D&cauthor=true&cauthor_uid=22778754), et al. The comparison of the efficacy of two different probiotics in rotavirus gastroenteritis in children. [J Trop Med.](http://www.ncbi.nlm.nih.gov/pubmed/?term=Erdogan+O+and+boulardii) 2012;2012:787240. doi: 10.1155/2012/787240

Eren M, Dinleyici EC, Vandenplas Y. [Clinical efficacy comparison of *Saccharomyces boulardii* and yogurt fluid in acute non-bloody diarrhea in children: a randomized, controlled, open label study.](http://www.ncbi.nlm.nih.gov/pubmed/20207879) Am J Trop Med Hyg. 2010;82(3):488-91. doi: 10.4269/ajtmh.2010.09-0529

Francavilla R, Lionetti E, Castellaneta S, Ciruzzi F, Indrio F, Masciale A, et al. Randomized clinical trial: *Lactobacillus reuteri* DSM 17938 vs placebo in children with acute diarrhea-a double-blind study. Alim Pharm Therapy. 2012;36:363-369. doi: 10.1111/j.1365-2036.2012.05180.x

Freedman SB, Sherman PM, Willan A, Johnson D, Gouin S, Schuh S; Pediatric Emergency Research Canada (PERC). Emergency department treatment of children with diarrhea who attend day care: A randomized multidose trial of a *Lactobacillus helveticus* and *Lactobacillus rhamnosus* combination probiotic. Clin Pediatr (Phila). 2015;54(12):1158-66. doi: 10.1177/0009922815569200

[Gaón D](http://www.ncbi.nlm.nih.gov/sites/entrez?Db=pubmed&Cmd=Search&Term=%22Ga%C3%B3n%20D%22%5BAuthor%5D&itool=EntrezSystem2.PEntrez.Pubmed.Pubmed_ResultsPanel.Pubmed_DiscoveryPanel.Pubmed_RVAbstractPlus), [García H](http://www.ncbi.nlm.nih.gov/sites/entrez?Db=pubmed&Cmd=Search&Term=%22Garc%C3%ADa%20H%22%5BAuthor%5D&itool=EntrezSystem2.PEntrez.Pubmed.Pubmed_ResultsPanel.Pubmed_DiscoveryPanel.Pubmed_RVAbstractPlus), [Winter L](http://www.ncbi.nlm.nih.gov/sites/entrez?Db=pubmed&Cmd=Search&Term=%22Winter%20L%22%5BAuthor%5D&itool=EntrezSystem2.PEntrez.Pubmed.Pubmed_ResultsPanel.Pubmed_DiscoveryPanel.Pubmed_RVAbstractPlus), [Rodríguez N](http://www.ncbi.nlm.nih.gov/sites/entrez?Db=pubmed&Cmd=Search&Term=%22Rodr%C3%ADguez%20N%22%5BAuthor%5D&itool=EntrezSystem2.PEntrez.Pubmed.Pubmed_ResultsPanel.Pubmed_DiscoveryPanel.Pubmed_RVAbstractPlus), [Quintás R](http://www.ncbi.nlm.nih.gov/sites/entrez?Db=pubmed&Cmd=Search&Term=%22Quint%C3%A1s%20R%22%5BAuthor%5D&itool=EntrezSystem2.PEntrez.Pubmed.Pubmed_ResultsPanel.Pubmed_DiscoveryPanel.Pubmed_RVAbstractPlus), [González SN](http://www.ncbi.nlm.nih.gov/sites/entrez?Db=pubmed&Cmd=Search&Term=%22Gonz%C3%A1lez%20SN%22%5BAuthor%5D&itool=EntrezSystem2.PEntrez.Pubmed.Pubmed_ResultsPanel.Pubmed_DiscoveryPanel.Pubmed_RVAbstractPlus), et al. Effect of *Lactobacillus* strains and *Saccharomyces boulardii* on persistent diarrhea in children. [Medicina (B Aires).](javascript:AL_get(this,%20'jour',%20'Medicina%20(B%20Aires).');) 2003;63(4):293-8.

[Grandy G](http://www.ncbi.nlm.nih.gov/pubmed/?term=Grandy%20G%5BAuthor%5D&cauthor=true&cauthor_uid=20735858), [Medina M](http://www.ncbi.nlm.nih.gov/pubmed/?term=Medina%20M%5BAuthor%5D&cauthor=true&cauthor_uid=20735858), [Soria R](http://www.ncbi.nlm.nih.gov/pubmed/?term=Soria%20R%5BAuthor%5D&cauthor=true&cauthor_uid=20735858), [Terán CG](http://www.ncbi.nlm.nih.gov/pubmed/?term=Ter%C3%A1n%20CG%5BAuthor%5D&cauthor=true&cauthor_uid=20735858), [Araya M](http://www.ncbi.nlm.nih.gov/pubmed/?term=Araya%20M%5BAuthor%5D&cauthor=true&cauthor_uid=20735858). Probiotics in the treatment of acute rotavirus diarrhoea. A randomized, double-blind, controlled trial using two different probiotic preparations in Bolivian children. [BMC Infect Dis.](http://www.ncbi.nlm.nih.gov/pubmed/?term=Grandy+G+and+diarrhea) 2010;10:253-260. doi: 10.1186/1471-2334-10-253

Guandalini S, Pensabene L, Zikri MA, Dias JA, Casali LG, Hoekstra H, et al. *Lactobacillus* GG administered in oral rehydration solution to children with acute diarrhea: a multicenter European trial. J Pediatr Gastroenterol Nutr. 2000; 30: 54-60.

Guandalini S, Magazzù G, Chiaro A, La Balestra V, Di Nardo G, Gopalan S, et al. VSL#3 improves symptoms in children with irritable bowel syndrome: a multicenter, randomized, placebo-controlled, double-blind, crossover study. J Pediatr Gastroenterol Nutr. 2010;51(1):24-30. doi: 10.1097/MPG.0b013e3181ca4d95

Guarino A, Canani RB, Spagnuolo MI, Albano F, Di Benedetto L. Oral bacterial therapy reduces the duration of symptoms and of viral excretion in children with mild diarrhea. J Pediatr Gastroenterol Nutri. 1997;25:516-9.

Guillot CC, Bacallao EG, Dominguez MSC, Garcia MF, Gutlerrez PM. Effects of *Saccharomyces boulardii* in children with chronic diarrhea, especially cases due to Giardiasis. Rev Mex de Puericultura Ypediatria. 1995;2:1-11.

Hafeez A, Tariq P, Ali S, Kundi ZU, Khan A, Hassan M. The efficiacy of *Saccharomyces boulardii* in the treatment of acute watery diarrhea in children: a multicentre randomized controlled trial. J Coll Physic Surg Pakistan. 2002;12:432-434.

Hernandez CL, Pineda EE, Jimenez MIR, Lucena MS. Clinical therapeutic effects of *Saccharomyces boulardii* on children with acute diarrhea. [In Spanish] Revista de Enfermedades Infecciosas en Pediatria*.* 1998;**11**(43):87–9.

[Htwe K, Yee KS, Tin M, Vandenplas Y.](http://www.ncbi.nlm.nih.gov/pubmed/18256417?ordinalpos=1&itool=EntrezSystem2.PEntrez.Pubmed.Pubmed_ResultsPanel.Pubmed_RVDocSum) Effect of *Saccharomyces boulardii* in the treatment of acute watery diarrhea in Myanmar children: a randomized controlled study. Am J Trop Med Hyg. 2008;78(2):214-6.

Isolauri E, Juntunen M, Rautanen T, Sillanaukee P, Koivula T. A human *Lactobacillus* strain (*Lactobacillus casei* sp strain GG) promotes recovery from acute diarrhea in children. Pediatrics. 1991; 88: 90-97.

Isolauri E, Kaila M, Mykkanen H, Ling WH, Salminen S. Oral bacteriotherapy for viral gastroenteritis. Dig Dis Sci. 1994; 39: 2595-600.

Kaila M, Isolauri E, Soppi E, Virtanen E, Laine S, Arvilommi H. Enhancement of the circulating antibody secreting cell response in human diarrhea by a human *Lactobacillus* strain. Pediatr Res. 1992; 32: 141-4. doi: 10.1203/00006450-199208000-00002

Kaila M, Isolauri E, Saxelin M, Arvilommi H, Vesikari T. Viable versus inactivated *Lactobacillus strain GG* in acute rotavirus diarrhoea. Arch Dis Child. 1995;72:51-53.

Khan A, Javed T, Chishti AL. Clinical efficacy of use of probiotic “*Saccharomyces boulardii*” in children with acute watery diarrhea. Pak Paed J. 2012:36(3):122-27.

Kurugöl Z, Koturoglu G. Effects of *Saccharomyces boulardii* in children with acute diarrhoea. Acta Paediatr. 2005;94(1):44-7.

Lahiri KR, Tullu MS, Raori R, Kondekar. Beneficial role of *Bacillus clausii* in treatment of acute diarrhea. PEDICON 2011. 48^th^ Annual National Conference of Indian Academy of Pediatrics . Jan 20-23, 2011. Abstract GE/06(0).

Lahiri K, Jadhav K, Gahlowt P, Najmuddin F. *Bacillus clausii* as an adjuvant therapy in acute childhood diarrhea. IOSR J Dental Med Sciences. 2015A;14(5):74-76.

Lahiri K, D’Souza J, Gahlowt P. Beneficial role of probiotic in acute childhood diarrhea. J Harmonized Research. 2015B;2(2):26-30.

[Le Luyer B](http://www.ncbi.nlm.nih.gov/pubmed/?term=Le%20Luyer%20B%5BAuthor%5D&cauthor=true&cauthor_uid=20236813), [Makhoul G](http://www.ncbi.nlm.nih.gov/pubmed/?term=Makhoul%20G%5BAuthor%5D&cauthor=true&cauthor_uid=20236813), [Duhamel JF](http://www.ncbi.nlm.nih.gov/pubmed/?term=Duhamel%20JF%5BAuthor%5D&cauthor=true&cauthor_uid=20236813). [A multicentric study of a lactose free formula supplemented with *Saccharomyces boulardii* in children with acute diarrhea].[Article in French] [Arch Pediatr.](http://www.ncbi.nlm.nih.gov/pubmed/?term=Le+Luyer+B+and+saccharomyces) 2010 May;17(5):459-65. [doi: 10.1016/j.arcped.2010.02.004.]

[Liévin-Le Moal V, Sarrazin-Davila LE, Servin AL.](http://www.ncbi.nlm.nih.gov/pubmed/17768180?ordinalpos=1&itool=EntrezSystem2.PEntrez.Pubmed.Pubmed_ResultsPanel.Pubmed_RVDocSum) An experimental study and a randomized, double-blind, placebo-controlled clinical trial to evaluate the antisecretory activity of *Lactobacillus acidophilus* strain LB against nonrotavirus diarrhea. Pediatrics. 2007;120(4):e795-803. doi: 10.1542/peds.2006-2930

Majamaa H, Isolauri E, Saxelin M, Vesikari T. Lactic acid bacteria in the treatment of acute rotavirus gastroenteritis. J Pediatr Gastroenterol Nutr. 1995; 20: 333-8.

Miele E, [Pascarella F](https://www.ncbi.nlm.nih.gov/pubmed/?term=Pascarella%20F%5BAuthor%5D&cauthor=true&cauthor_uid=19174792), [Giannetti E](https://www.ncbi.nlm.nih.gov/pubmed/?term=Giannetti%20E%5BAuthor%5D&cauthor=true&cauthor_uid=19174792), [Quaglietta L](https://www.ncbi.nlm.nih.gov/pubmed/?term=Quaglietta%20L%5BAuthor%5D&cauthor=true&cauthor_uid=19174792), [Baldassano RN](https://www.ncbi.nlm.nih.gov/pubmed/?term=Baldassano%20RN%5BAuthor%5D&cauthor=true&cauthor_uid=19174792), [Staiano A](https://www.ncbi.nlm.nih.gov/pubmed/?term=Staiano%20A%5BAuthor%5D&cauthor=true&cauthor_uid=19174792). Effect of a probiotic preparation (VSL#3) on induction and maintenance of remission in children with ulcerative colitis. Am J Gastroenterol. 2009;104(2):437-43. doi: 10.1038/ajg.2008.118

[Ozkan TB, Sahin E, Erdemir G, Budak F.](http://www.ncbi.nlm.nih.gov/pubmed/17542407?ordinalpos=2&itool=EntrezSystem2.PEntrez.Pubmed.Pubmed_ResultsPanel.Pubmed_RVDocSum) Effect of *Saccharomyces boulardii* in children with acute gastroenteritis and its relationship to the immune response. J Int Med Res. 2007;35(2):201-12. doi: 10.1177/147323000703500204

Pant AR, Graham SM, Allen SJ, Harikul S, Sabchareon A, Cuevas L, et al. *Lactobacillus* GG and acute diarrhea in young children in the tropics. J Trop Pediatr. 1996; 42: 162-5.

Pedone CA, Bernabeu AO, Postaire ER, Bouley CF, Reinert P. The effect of supplementation with milk fermented by *Lactobacillus casei* (strain DN-114 001) on acute diarrhoea in children attending day care centres. Int J Clin Pract. 1999; 53: 179-84.

Raza S, Graham SM, Allen SJ, Sultana S, Cuevas L, Hart CA. Lactobacillus GG promotes recovery from acute nonbloody diarrhea in Pakistan. Pediatr Infect Dis J. 1995; 14: 107-11.

[Riaz M](http://www.ncbi.nlm.nih.gov/pubmed/?term=Riaz%20M%5BAuthor%5D&cauthor=true&cauthor_uid=21997865), [Alam S](http://www.ncbi.nlm.nih.gov/pubmed/?term=Alam%20S%5BAuthor%5D&cauthor=true&cauthor_uid=21997865), [Malik A](http://www.ncbi.nlm.nih.gov/pubmed/?term=Malik%20A%5BAuthor%5D&cauthor=true&cauthor_uid=21997865), [Ali SM](http://www.ncbi.nlm.nih.gov/pubmed/?term=Ali%20SM%5BAuthor%5D&cauthor=true&cauthor_uid=21997865). Efficacy and safety of *Saccharomyces boulardii* in acute childhood diarrhea: a double blind randomised controlled trial. [Indian J Pediatr.](http://www.ncbi.nlm.nih.gov/pubmed/?term=Riaz+M+and+boulardii) 2012;79(4):478-82. doi: 10.1007/s12098-011-0573-z

Salazar-Lindo E, Figueroa-Quintanilla D, Caciano MI, Reto-Valiente V, Chauviere G, et al. Effectiveness and safety of *Lactobacillus* LB in the treatment of mild acute diarrhea in children. J Pediatr Gastroenterol Nutr. 2007; 44(5):571-6. doi: 10.1097/MPG.0b013e3180375594

Savaş-Erdeve S, Gökay S, Dallar Y. Efficacy and safety of *Saccharomyces boulardii* in amebiasis-associated diarrhea in children. [Turk J Pediatr.](javascript:AL_get(this,%20'jour',%20'Turk%20J%20Pediatr.');) 2009;51(3):220-4.

Shan L, Hou P, Wang Z, Chen N, Shu L, Zhang H, et al. Prevention and treatment of diarrhea with *Saccharomyces boulardii* in children with acute lower respiratory tract infections. Bene Microbes*.* 2013;4(4):329-334. doi: 10.3920/BM2013.0008

Simakachorn N, Pichaipat V, Rithipornpaisarn P, Kongkaew C, Tongpradit P, Varavithya W. Clinical evaluation of the addition of lyophilized, heat-killed *Lactobacillus* *acidophilus* LB to oral rehydration therapy in the treatment of acute diarrhea in children. J Ped Gastroenterol Nutr. 2000; 30: 68-72.

[Sindhu KN](https://www.ncbi.nlm.nih.gov/pubmed/?term=Sindhu%20KN%5BAuthor%5D&cauthor=true&cauthor_uid=24501384), [Sowmyanarayanan TV](https://www.ncbi.nlm.nih.gov/pubmed/?term=Sowmyanarayanan%20TV%5BAuthor%5D&cauthor=true&cauthor_uid=24501384), [Paul A](https://www.ncbi.nlm.nih.gov/pubmed/?term=Paul%20A%5BAuthor%5D&cauthor=true&cauthor_uid=24501384), [Babji S](https://www.ncbi.nlm.nih.gov/pubmed/?term=Babji%20S%5BAuthor%5D&cauthor=true&cauthor_uid=24501384), [Ajjampur SS](https://www.ncbi.nlm.nih.gov/pubmed/?term=Ajjampur%20SS%5BAuthor%5D&cauthor=true&cauthor_uid=24501384), [Priyadarshini S](https://www.ncbi.nlm.nih.gov/pubmed/?term=Priyadarshini%20S%5BAuthor%5D&cauthor=true&cauthor_uid=24501384), et al. Immune response and intestinal permeability in children with acute gastroenteritis treated with *Lactobacillus rhamnosus* GG: a randomized, double-blind, placebo-controlled trial. [Clin Infect Dis.](https://www.ncbi.nlm.nih.gov/pubmed/?term=Sindhu+and+LGG) 2014;58(8):1107-15. doi: 10.1093/cid/ciu065

Tlaskal P, Michkova E, Klayarova H, Jerabkova L, Nevoral J, Balackova J, et al. *Lactobacillus acidophilus* in the treatment of children with gastrointestinal illnesses. Česko-Slovenská Pediatrie. 1995;51: 615-619.

Tlaskal P, Schramlova J, Kokesova A, Adamus J, Bubakova D. Kocnarova N, et al. Probiotics in the treatment of diarrhoeal disease of children. Nutrition Aliments Fonctionnels Aliments Santé. 2005; 3: 25-28.

Urganci N, Polat T, Uysalol M, Cetinkaya F. Evaluation of the efficacy of *Saccharomyces boulardii* in children with acute diarrhea. Arch Gastroenterol. 2001; 20(3-4):1-7.

Vandenplas Y, Badriul H, Thapa B, Elizabeth K, Bhave S. A multicenter DBRC-trial in developing countries with *Saccharomcytes boulardii* (*S. boulardii*) in acute gastroenteritis. Abstract #PG2-21. Presented at the European Society for Paediatric Gastoenterology, Hepatology and Nutrition Meeting. May 9-12, 2007, Barcelona, Spain. J Pediatr Gastoenterol Nutri. 2007;44(6):e86.

[Villarruel G](http://www.ncbi.nlm.nih.gov/sites/entrez?Db=pubmed&Cmd=Search&Term=%22Villarruel%20G%22%5BAuthor%5D&itool=EntrezSystem2.PEntrez.Pubmed.Pubmed_ResultsPanel.Pubmed_DiscoveryPanel.Pubmed_RVAbstractPlus), [Rubio DM](http://www.ncbi.nlm.nih.gov/sites/entrez?Db=pubmed&Cmd=Search&Term=%22Rubio%20DM%22%5BAuthor%5D&itool=EntrezSystem2.PEntrez.Pubmed.Pubmed_ResultsPanel.Pubmed_DiscoveryPanel.Pubmed_RVAbstractPlus), [Lopez F](http://www.ncbi.nlm.nih.gov/sites/entrez?Db=pubmed&Cmd=Search&Term=%22Lopez%20F%22%5BAuthor%5D&itool=EntrezSystem2.PEntrez.Pubmed.Pubmed_ResultsPanel.Pubmed_DiscoveryPanel.Pubmed_RVAbstractPlus), [Cintioni J](http://www.ncbi.nlm.nih.gov/sites/entrez?Db=pubmed&Cmd=Search&Term=%22Cintioni%20J%22%5BAuthor%5D&itool=EntrezSystem2.PEntrez.Pubmed.Pubmed_ResultsPanel.Pubmed_DiscoveryPanel.Pubmed_RVAbstractPlus), [Gurevech R](http://www.ncbi.nlm.nih.gov/sites/entrez?Db=pubmed&Cmd=Search&Term=%22Gurevech%20R%22%5BAuthor%5D&itool=EntrezSystem2.PEntrez.Pubmed.Pubmed_ResultsPanel.Pubmed_DiscoveryPanel.Pubmed_RVAbstractPlus), [Romero G](http://www.ncbi.nlm.nih.gov/sites/entrez?Db=pubmed&Cmd=Search&Term=%22Romero%20G%22%5BAuthor%5D&itool=EntrezSystem2.PEntrez.Pubmed.Pubmed_ResultsPanel.Pubmed_DiscoveryPanel.Pubmed_RVAbstractPlus), et al. *Saccharomyces boulardii* in acute childhood diarrhoea: a randomized, placebo-controlled study. [Acta Paediatr](javascript:AL_get(this,%20'jour',%20'Acta%20Paediatr.');). 2007;96(4):538-41. doi: 10.1111/j.1651-2227.2007.00191.x
