# Supplementary material for: Choosing an appropriate probiotic product for your patient: An evidence-based practical guide
Source: PLoS One. 2018 Dec 26;13(12):e0209205. doi: 10.1371/journal.pone.0209205 (PMC6306248; doi:10.1371/journal.pone.0209205)
Supplement: S1 Dataset — (DOCX) [file pone.0209205.s004.docx]

**Lynne McFarland Probiotic Dataset**

**For randomized controlled trials published 1977-2017
Probiotic interventions for selected disease indications**

**Data extracted from published randomized controlled trials or meeting abstracts. Supplementary data indicated by colored font from author(s) of paper or from manufacturer website. Trials with multiple study arms in one study are indicated by grey rows. Data entered in chronological order and by mode (prevention or treatment) and by disease indication.**

**All errors are the responsibility of Dataset creator/administrator (Lynne McFarland).
Send queries to: Lynne.Mcfarland.v@gmail.com**

**-------------------------------------------------------------------------------------------------------------------**

Table of Contents

[Common abbreviations: 2](#_Toc531247966)

[Prevention 3](#_Toc531247967)

[Allergies: Prevention 3](#_Toc531247968)

[Antibiotic-associated diarrhea (AAD): Prevention 4](#_Toc531247969)

[Clostridium difficile infections (CDI)- Primary prevention 19](#_Toc531247970)

[Enteral feeding: prevention of diarrhea 26](#_Toc531247971)

[Helicobacter pylori trials: prevention of adverse reactions 27](#_Toc531247972)

[Necrotizing enterocolitis (NEC)- prevention 31](#_Toc531247973)

[Nosocomial Infections-prevention 34](#_Toc531247974)

[Respiratory Infections – prevention 35](#_Toc531247975)

[Surgical Infections-preventive 37](#_Toc531247976)

[Travellers’ diarrhea- Prevention 40](#_Toc531247977)

[Urinary Tract Infections- prevention 42](#_Toc531247978)

[Treatment 43](#_Toc531247979)

[Adult Acute Diarrhea-treatment 43](#_Toc531247980)

[Clostridium difficile infections (CDI): Treatment 45](#_Toc531247981)

[Pediatric Colic-Treatment 46](#_Toc531247982)

[Constipation- treatment 47](#_Toc531247983)

[H. pylori treatment 48](#_Toc531247984)

[Inflammatory Bowel Disease (IBD)- treatment 61](#_Toc531247985)

[Irritable Bowel Syndrome (IBS)-treatment 65](#_Toc531247986)

[Pediatric acute diarrhea-treatment 71](#_Toc531247987)

## Common abbreviations:

[other abbreviations may be found in table footnotes]

**AAD**: antibiotic associated diarrhea

**B. or Bif.:** Bifidobacterium

**CDI:** *Clostridium difficile* infections

**cfu:** colony-forming units (number of microbiologic colonies)

**F/up:** follow-up post-probiotic intervention

**IBS:** irritable bowel disease

**IBD:** inflammatory bowel disease

**L.:** Lactobacillus

**mon**: months

**nr:** not reported

**ns:** not significantly different from controls (P>0.05)

**S.:** Saccharomyces

**VSL#3**: 8-strain mixtures of:*3 Bifido (longum and infantis and breve) +4 Lacto. (acidophilus and casei and delbrueckii and plantarum) and Strept thermophilus.*

# Prevention

## Allergies: Prevention

Atopic dermatitis (atopic eczema) defined as a type of inflammation of the skin (itchy, red, swollen) and was diagnosed by physician by standard criteria.

| **+/-** | **Probiotic** | **Daily dose (cfu/day) and to whom** | **Population studied, country and (attrition)** | **Duration (& follow-up)** | **Probiotic outcome: AD** | **Controls AD** | **Reference** |
| --- | --- | --- | --- | --- | --- | --- | --- |
| + | *Lactobacillus rhamnosus* GG (ATCC 53103) | 2 x 10^10^  Capsules to mothers (2-4 wks before delivery), then 6 mon post-natally if breast feeding.  If not-just powder in liquid to babies to 6 months | 159 mother infant pairs enrolled with family history of allergy; 132 done (17% attrition )   FINLAND | 6 months  F/up: 1.5 yr  [until 2 yrs old] | By aged 2 yrs: atopic dermatitis 15/64 (23%)  P<0.05 | placebo 31/68 (46%) | **Kalliomaki** M   2001  Lancet |
| - | *Lactobacillus rhamnosus* GG  (ATCC 53103) | 2 x 10^10^/d | young adults with birch allergy and apple allergy  38 enrolled, 31 done (18% attrition) FINLAND | 5.5 months   during 1999 pollen season  F/up: none | Change in allergic rhinitis symptoms scores:  +7.8 (-0.8, +16.4) NS | Placebo +13.6 (+3.2, +23.9) | **Helin** T   2002   Allergy |
| - | *Lactobacillus rhamnosus* GG  (ATCC 53103) | 1 x 10^10^/d to mothers (4-6 wks pre delivery, then 3 mon) and to babies 3 mon- 6 mon old | 105 pregnant women with family history of AD,   94 done (10% attrition)  GERMANY | 7.5 wks  F/up: until babies were 2 yrs old | At 2 yrs:  14/50 (28%) babies developed Atopic dermatitis, NS | At 2 yrs: placebo 12/44 (27%) | **Kopp** MV   2008  Pediatrics |

+/-, positive efficacy or negative (not significant) efficacy; AD: atopic dermatitis; cfu, colony-forming units; F/up, follow-up; NS, not significantly different; mon, months

## Antibiotic-associated diarrhea (AAD): Prevention

page 1

AAD typically defined as: diarrhea (>3 loose/watery stools/d for >2 days) associated with antibiotic exposure or <8 weeks post-antibiotic discontinuation (delayed onset).

| **+/-** | **Probiotic** | **Population** | **Daily dose** | **Duration** | **AAD in Probiotic** | **AAD**  **in controls** | **Reference** |
| --- | --- | --- | --- | --- | --- | --- | --- |
| *+* | *S. boulardii* vs placebo | 388 hospitalized adults FRANCE | 4 x 10^9^ **capsules** | 7 days F/up: nr | **AAD**: 4.5%*  9/199 | AAD 17.5%  33/189 | **Adam** J  1976 Gaz Med Fra |
| *+* | *S. boulardii* vs placebo | 240 adults on oral antibiotics PORTUGAL | 4 caps/d, dose nr in full paper **capsules** | 6 days,  F/up: 0 | **AAD** (>2 BM/d):  19/121 (15.7%)* | placebo:  33/119 (27.7%) | **Monteiro** E  1981 Acta Med Port |
| *+* | *Enterococcus faecium* SF68 | 200 adults with tuberculosis | nr | 60 days F/up: nr | **AAD**: 5/100 (5%)* | 18/100 (18%) | **Borgia** M  1982 Curr Ther Res |
| *+* | *Entero. faecium* SF68 vs placebo | 1323 adults on antibiotic | 2 caps/d cfu nr **capsules** | 7 days  F/up: nr | **AAD**: 57/661 (9%) P<0.001 | 107/662 (16%) | **Frigerio** G 1986  Dig Dis Sci 31(10 Suppl):496S Mtg Abstract only |
| *+* | *S. boulardii* vs placebo | 180 hospitalized adults | 2 x 10^10^  **capsules** | 28 days  F/up: mean of 17 days | **AAD**: 9.5%* 11/116 | 21.8% 14/64 | **Surawicz** CM 1989  Gastroenterol |
| *-* | *Enterococcus faecium* SF68 | 45 adult patients | 1.5 x 10^7^ | 7 days F/up: nr | **AAD**: 8.7% ns 2/23 | 27.2% 6/22 | **Wunderlich** PF 1989  J Int Med Res |
| *+* | *S. boulardii* vs placebo | 193 hospitalized adults (1 or more beta-lactam antibiotics) | 2 x 10^10^  **capsules** | duration antibiotic plus 3 days,  F/up: 7 wks | **AAD**: 7.2%* 7/97 On abx 5/97(5.1%) ns  post abx 2/92 (2.3%) | AAD: 14.6% 14/96 On abx 8/96 (8.3%) post abx 6/84 (7.1%) p=0.15 | **McFarland** LV 1995 Am J Gastro |

*, P<0.05; AAD, antibiotic-associated diarrhea; abx, antibiotics; AE, adverse events; cfu, colony-forming units; F/up, follow-up; nr, not reported; NS, not significantly different; mon, months; txt, treatment; wks, weeks

**AAD page 2**

| **+/-** | **Probiotic** | **Population** | **Daily**  **dose** | **Duration** | **AAD in Probiotic** | **AAD**  **in controls** | **Reference** |
| --- | --- | --- | --- | --- | --- | --- | --- |
| *-* | *S. boulardii* vs placebo | 72 enrolled, (4% attrition) done: 69 elderly (>65 yrs old) inpatients on abx (70-85 yrs old) U.K. | 4.5 X 10^9^  226 mg/d  **capsules** | duration abx (mean 7 days)  F/up: 0 | **AAD**: 7/33 (21%) ns p=0.53 | AAD: 5/36 (13.9%) | **Lewis** SJ  1998 J Infect |
| **-** | *L. rhamnosus GG “*Gefilus*”* whey drink vs milk control | 59 children (5 mon-11 yrs) outpatients, for otitis media, all on amoxicillin FINLAND | 8 x 10^10^ 200 ml/d **drink** | 7 days  F/up: 0 | **AAD** 26% ns  6/23 | milk control  22%  8/36 | **Vaisanen** ML 1998 Abstract Micro Ecol Health & Dis |
| **+** | *L. rhamnosus* GG vs placebo | 167 children (2 wks-13 yrs old) enrolled, 119 done (29% attrition due to long f/up) 66% ampic. outpatients with URT Infections (26%) or otitis media (74%) FINLAND | 4 x 10^10^ **capsules** | duration abx. (mean=7-10 days)   follow-up: 3 months  APP | **AAD** 3/61 (5%) * | **AAD** 9/58 (16%) | **Arvola** T 1999 Pediatrics |
| **-** | *S boulardii versus control (diosmectitic-anti-spasmodic)* double blinded | 779 enrolled, 616 done, children (1-5 yrs) with resp infection, mix of antibiotics, outpatients **(21% attrition)**  FRANCE | 4.5 x 10^9^  226 mg/d  **capsules** | Abx duration  mean=8 + 2 days  F/up: 0 | **AAD**: 25/327 (7.6%), p=0.29 ns | anti-spasmodic control: 16/289 (5.5%) | **Benhamou** PH 1999  Med Chir Dig |
| **+** | *L. rhamnosus* GG + inulin vs placebo | 202 children enrolled (6-120 mon old) outpatients on mix of abx,  188 done (**7% attrition) USA** | 1-2 x 10^10^ **capsules** | 10 days  F/up: 0  APP | **AAD** 7.5%* (7/93) p=0.001 duration: 4.7 d | inulin & placebo: 26% (25/95) duration: 5.9 days | **Vanderhoof** JA 1999 J Ped |

**AAD- page 3**

| **+/-** | **Probiotic** | **Population** | **Daily**  **dose** | **Duration** | **AAD in Probiotic** | **AAD**  **in controls** | **Reference** |
| --- | --- | --- | --- | --- | --- | --- | --- |
| *-* | *L. rhamnosus* GG vs placebo | 302 enrolled,  267 done (11.6% attrition) adult inpatients | 2 x 10^10^ **capsules** | 14 days F/up: nr  APP | **AAD**  (29.3%) 39/133 ns | AAD (29.9%) 40/134 | **Thomas** MR 2001 Mayo Clin Proceed |
| *+* | *Lactobacillus rhamnosus GG  "*GiFlorex*"* vs nothing.  open study | 120 asymptom. Hp+ carriers Hospital staff, adults. May-July 1999, one site, 117 done (2.5% lost)  All on triple therapy for 7 days: (claritho, pantoprazole, tinidazole)  ITALY | 1.2 x 10^10^ **Sachet** | 14 days  F/up:  6 wks  ITT | **AAD**: 8/60 (13.2%) P<0.001  **Hp-:** 48/60 (80%), p=0.6  **Any AE**: 26 (43%) p=0.04 | control AAD: 29/60 (48.2)  Hp-: 46/60 (76.6%)  Any AE: 37 (62%) | **Armuzzi** A 2001 **A** Digestion |
| *+* | *Lactobacillus rhamnosus GG  "*GiFlorex*"* vs placebo blinded study All on triple therapy for 7 days (claritho, rabeprazole, tinidazole) | 60 asymptom. Hp+ carriers Hospital staff, adults. Sept 1999 -Jan 2000  0% attrition  ITALY | 1.2 x 10^10^  **Sachet** | 14 days  F/up:  6 wks  ITT | **AAD:** 1/30 (3.3%), p=0.01 10%*  **Hp neg**: 25/30 (83.3%) p=1.0 **Any AE**: 12/30 (40%)* p=0.04 | placebo: AAD: 8/30 (26.6%)   Hp-: 24/30 (80%)   Any AE: 20/30 (67%) | **Armuzzi** A 2001 **B** Alim Pharm & Therapeutics |

**AAD page 4**

|  | **Probiotic** | **Population** | **Daily**  **dose** | **Duration** | **AAD in Probiotic** | **AAD**  **in controls** | **Reference** |
| --- | --- | --- | --- | --- | --- | --- | --- |
| *-* | *L. rhamnosus* GG vs placebo | 42 *H. pylori* + on triple therapy, adults | 6 x 10^9^ | 7 days F/up: 4 wks | **AAD**: 5% ns 1/21 p=0.09 | 30% 6/21 | **Cremonini** F  2002B  Amer J Gastroenterol |
| *+* | *S. boulardii* vs placebo | 43 *H. pylori* + on triple therapy, adults | 5 x 10^9^ | 7 days F/up: 4 wks | **AAD**: 5% 1/22* p=0.046 | 30% 6/21 | **Cremonini** F  2002B  Amer J Gastroenterol |
| *+* | *L. acidophilus* La5 + *Bifido animalis* subsp *lactis* Bb12  [*Strept thermo* *L. bulgaricus* yogurt  “AB Yogurt” vs ‘no yogurt’ control- | 160 outpatient adults *H. pylori* + (mean age 48 yrs) all on triple therapy (2 abx + PPI) open design, not blinded, no placebo | 1 x 10^10^ | 4 wks   F/up: 4 wks | **AAD**: 2/80 (2.5%) p=0.03 | no txt control: AAD 10/80 (12.5%) | **Sheu** BS  2002 Ali Pharm Ther |
| *+* | *Clostridium butyricum* MIYAIRI vs no txt controls | 110 children,[72 completed] (1-180 months), on mostly cephalosporins or penicillin for URT or gastro infection (34% attrition) JAPAN | 1-4 x 10^7^  **capsules** | 6 days  F/up: 0 | **AAD**: 6/86 (7%)*  Claim “normalizes GI flora disturbed by Abx” &  more anaerobes | no txt control, not blinded  59%  16/27 | **Seki** H  2003 Pediatr Intl |
| *+* | *S. boulardii* vs nothing for each abx group: No placebo Open trials | 466 children  (1-15 yrs old) on sulbactam-ampicillin (SAM) or on azthromycin (AZT), outpatients (TURKEY | 250 mg/d  5 x 10^9^ **sachets** | during abx  F/up: 2 wks post abx | **AAD**: 7/117 (5.7%) on SAM*  but 7/127 AZT, ns | 30/117 (25.6%) SAM only.  12/105 (11.4%) AZT only | **Erdeve** O  2005 J Trop Ped |

**AAD page 5**

| **+/-** | **Probiotic** | **Population** | **Daily dose** | **Duration** | **AAD in Probiotic** | **AAD in controls** | **Reference** |
| --- | --- | --- | --- | --- | --- | --- | --- |
| + | *S. boulardii* vs placebo.  with otitis media or resp infections only 36% power | 269 children (6 mon-14 yrs) enrolled,  246 children finished (8.6% attrition),  in and outpatient POLAND | 1 x 10^10^ 500 mg/d  **wafers** | Varied during abx.  7-9 d (Sb) & 5-13 d (placebo)  F/up: 2 wks | **AAD**: 9/119 (8%)* onset: 4.8 + 2.5 (2-8 days) excluding rotaviral: 4/119 (3.4%) | **AAD**: 29/127 (23%) onset: 4.9 + 3 (1-11 days) excluding rotaviral: 22/127 (17.3%) | **Kotowska** M 2005 Ali Phar Ther |
| *-* | *C. butyricum* MIYAIRI 588 vs no probiotic control | 35 *H. pylori*+ adults all with 1 week triple therapy JAPAN | 120 mg tid, cfu/d nr | 2 wks  F/up: 6 wk | **AAD** 5/18 (27.8%) p=0.09 Hp neg: 13/17 (94%) ns | AAD 10/17 (58.8) Hp neg: 13/17 (73%) | **Shimbo** I 2005  World J Gastroenterol |
| *+* | *S. boulardii* “Reflor”, Sanofi-Sythelabo) vs ‘no Sb’ control (no placebo) | adults peptic ulcers on abx. 389 enrolled, 376 completed all given Hp+ triple therapy (amox+clarithro+PPI) in 9 hospitals TURKEY | 1 x 10^10^  1 gram/d | 2 wks &  F/up: 4 wks | **AAD**: 14/204 (6.9%*) p=0.007  AAD on Abx (86%) | AAD: 28/185 (15.1%)   AAD on Abx (75%) | **Duman** DG 2005  Eur J Gast Hepta |
| *+* | *S. boulardii* vs placebo | 151 adult inpatients | 1 x 10^10^ | duration abx F/up: ~10 days post-abx | **AAD**: 1.4%* (1/73) 1 case on abx (day 2 of abx) postabx 0% p=0.06 | 9.0% (7/78) On abx: 2/78 (2.6%) & post-abx (5/76, 6.6%) 5-10 days | **Can** M  2006 Med Sci Monit |
| *-* | *"*AB Yogurt" *L acidophilus La5 + Bif. animalis subsp lactis Bb12* [starters: *L. bulgaricus + Strept thermophilus*]. vs no txt control All on **quadruple** therapy (AmoxMetroOmeBs) (7 days) | 138 adults who failed triple Hp therapy with ulcers or gastritis,  129 done (6% attrition)  TAIWAN | 400 ml/d  4 x 10^11^/d   **Yogurt**  single blinded (Hp assessor) | 4 wks  F/up:  6 wks & 3 months if neg at 6 wks.  ITT | **AAD:** 9/69 (8.7%)  p=0.053 Chi^2^  Hp-: 59/69 (85%)* P=0.04 Any AE**:** patient level data nr | **AAD:** 18/69 (26%)  Hp-: 49/69 (71%)  Any AE**:** nr | **Sheu** BS 2006 Am J Clin Nutri |

**AAD page 6**

|  | **Probiotic** | **Population** | **Daily**  **dose** | **Duration** | **AAD in Probiotic** | **AAD in controls** | **Reference** |
| --- | --- | --- | --- | --- | --- | --- | --- |
| *+* | *L. casei DN114001 [L. bulgaricus) S. thermophilus]* Actimel drink vs Yazoo (milkshake) | 135 adult inpatients  (>50 yrs old) on abx, 112 completed (16% attrition) 89/135 (66%) on high risk antibiotics  (Table 1) | 1.9 x 10^10^  per day **drink** | duration abx + 1 week  F/up: 0 APP | **AAD** 7/57 (12.3%)* p=0.006  if on Abx:  4 (6.2%) ns  post-Abx: 3 (5.7%) P=0.003 | placebo AAD 19/56 (33.9%)  if on Abx; 5 (8.1%) ns, p=0.74 post-abx:  14 **(**27.5**%)** p=0.003 peak 8 d | **Hickson** M 2007 Brit Med J |
| *+* | “Lacidofil” *L. [acidophilus] helveticus R52 + L. rhamnosus* R11 vs open controls | n=34 children (10 mon-3 yrs old) with resp tract infections on abx. **UKRAINE** | 2-4 x 10^9^/d [depends on age] **capsules** | 2-4 weeks varied on antibiotic duration  F/up: nr | **AAD:** 2/16 (12.6%) p<0.05  **duration diarrhea**: 2.6 + 1.1 p<0.05 | open controls AAD: 8/18 (44.8%) **duration**: 5.9 + 1.2 d | **Marushko** YV 2007 Perinatol & Paediatrics |
| *-* | *L. rhamnosus GG* vs. placebo  (maltodextrine)  All given 7 d of 2 abx (amox and clarithr) and PPI | 83 kids (5-17 yrs old)., 64 done (23% attrition) inpatients *H. pylori +* eradication,. **POLAND** | 1 x 10^9^ /d **capsules** | 7 days  F/up: 6 wks  APP | **AAD**: 2/34 (6%) NS  *H pylori* neg: 23/34 (69%) NS | AAD: 6/30 (20%) Hp- : 22/32 (68%) | **Szajewska** H  2009  J Ped Gastroenterol & Nutrition |
| *+* | “Lacidofil” *L. helveticus* R0052 + *L. rhamnosis* R0011 vs open controls | n=36 children with cystic fibrosis on abx  (<1 yr old) | 1 capsule bid-tid varied by age range  **capsule** | varied  F/up: nr | **AAD**: 1/18 (5.5%) p<0.05 | open **AAD**: 5/18 (28.9%) | **Aryayev** M 2009 Odessa Med J Mtg abstract |

**AAD page 7**

|  | **Probiotic** | **Population** | **Daily**  **dose** | **Duration** | | **AAD in Probiotic** | **AAD**  **in controls** | **Reference** |
| --- | --- | --- | --- | --- | --- | --- | --- | --- |
| *+* | *“BioK+” (L. acido CL1285 + L. casei LBC80R + L. rhamnosus CLR2)* vs placebo milk | 89 hosp adults (mean >59 yrs old) on a variety of abx 1 hospital in Quebec  **CANADA** | 5 x 10^10^   **milk**  ITT | 8-10 d, duration of antibiotic,  F/up: 21 d | | **AAD**: 7/44 (15.9%)* p=0.03  **AE**: 21(48%) | **AAD** 16/45 (35.6%)  **AE**: 20 (44%) | **Beausoleil** M 2007  Can J Gastroe stopped early cuz she got her Masters and graduated |
| *+* | *S. boulardii* (“Reflor”) in Turkey BCX, vs nothing; both on triple therapy, open | 124 adults outpatients with *H. pylori* + dyspepsia TURKEY | 2 x 10^10^  (1g/d) **sachets** | 2 wks   F/up: 6 wks | **AAD**: 9/62 (14.5%)* Hp- (71%) ns CDI 6/62 (9.7%) p=0.8 | | 19/62 (30.6%)  Hp- 60% CDI 8/62 (12.8%) | **Cindoruk** M 2007 Helicobacter  blue: data from author |
| *-* | *S. boulardii*  vs placebo | 86 adult outpatients on amoxicillin (5-10 d) | 1 x 10^10^ /d | 12 days  F/up: 9 days  4% power | | **AAD**: 3/41 (7.3%) p=0.72 **duration diarrhea**: 11.4 +2.0 d | AAD: 5/45 (11.1%) duration: 11.5 + 2.2 d | **Bravo** MV 2008  Rev Med Chile translated from Spanish |
| *-* | *C. butyricum* CBM588 (low dose)  vs control | 19 adults with GI ulcers and H. pylori + unclear if outpatient or inpatient JAPAN | 6 **tablets** cfu/d not reported | 1 week  F/up: 0 | | **AAD**: single dose 1/12 (8.3%) ns | No probiotic control: AAD 3/7 (43%) | **Imase** K  2008  Microbiol Immunol |
| *-* | *“Lacidofil” (L helveticus R52+L. rhamnosus R11)* | not randomized in 45 children  Ukraine | 4 x 10^9^ **capsules** | 3 wk  F/up: 1 wk | | **AAD** 2/25 (8%) p=0.06 | 7/20 (35%) | **Gnaytenko** O  2009 Prac Med |

**AAD- page 8**

| **+/-** | **Probiotic** | **Population** | **Daily dose** | **Duration** | **AAD in Probiotic** | **AAD**  **in controls** | **Reference** |
| --- | --- | --- | --- | --- | --- | --- | --- |
| *-* | *L rhamnosus GG* vs placebo | N=316 inpatient adults on antibiotics  CANADA | 1.2 x 10^11^ /d | 14 days  F/up: 4 wks | **AAD**: 8/157 (5.1%), p=0.18  **AE**: 4/157 (2.5%) ns | **AAD**: 4/159 (2.5%)   AE: 0/159 (0%) | **Miller** M 2008**B** Meeting Abstract ICAAC Washington DC |
| *+* | “Lacidofil WM” *L. helveticus R52 + L. rhamnosus R11* | n=96 adult women with C-sections and cefotaxime pre-op abx. | 6 x 10^9^/d   (3 caps/d)  **capsules** | 7 days  F/up: none | (n=56) **AAD**: 0/56 (0%) p<0.05 | (n=40) **AAD**: 4/40 (10%) | **Liskovich** VV 2010 Health |
| *-* | *S. boulardii* (Bioflor) vs  S.b. + mucoprotective agent (DA9601)  vs control.  All on triple therapy | Adult outpatients, N=991, *H. pylori*+ outpatients **Korea** | 3 x 10^10^  **capsules** | 4 wks  F/up: 4 wks | Sb only: **AAD** 11/330 (3%) p=0.14  Sb+mucop agent: 9/330 (3%), n=0.06 | AAD in control: 20/331 (6%) | **Song** M 2010 Heliobacteria |
| *-* | *“Lacidofil” cap L. rhamnosus (Rosell-11) +  L. helveticus (Rosell-52) vs placebo caps* | 214 adults (>18 yrs old) on abx<48 hrs for resp infections in 10 hospitals  KOREA ITT analysis | 2 x 10^9^/cap  **capsules** | within 48 hrs for 14 days.  No f/up | **AAD** 4/103 (3.9%), ns CDI data, NS | AAD: 8/111 (7.2%) | **Song** HJ 2010  Gastr & Heptaol |

|  | **Probiotic** | **Population** | **Daily**  **dose** | **Duration** | **AAD in Probiotic** | **AAD**  **in controls** | **Reference** |
| --- | --- | --- | --- | --- | --- | --- | --- |
| *+* | *BioK+  (L. acido CL1285 + L. casei LBC80R + L. rhamnosus CLR2)    low dose (1 capsule) vs placebo* | (Oct 2008-Mar 2009)   255 eligible adults (50-70 yrs old)   on pen, ceph, clinda  at 1 hospital  **Shanghai** | Low dose (1 cap/d)  5 x 10^10^ **capsule** | duration abx’s (3-14 days plus 5 more days  Followed 21 days more. | **Low** dose Bio-K AAD 24/85, (28.2%) p=0.02  Duration AAD:  4.1+ 1.5* d **Abd pain:** 24.7% ns | placebo (n=84)  AAD 37/84 (44.1%)  Duration= 6.4+ 1.8 days  Abd pain: 40.5% | **Gao** XW  2010  Amer J Gastroenterol |
| *+* | *BioK+  (L. acido CL1285 + L. casei LBC80R + L. rhamnosus CLR2)   high dose: 2 capsules vs placebo* |  | High dose  (2 caps/d)  1 x 10^11^  **capsule** |  | **Higher** dose (2 caps) **AAD** 13/86, (15.5%) P<0.001) (duration=2.8 + 0.8* d) **Abd pain**: 12.8% |  | **Gao** XW  2010  Amer J Gastroenterol |
| *trend* | *“BioK+” (L. acido CL1285 + L. casei LBC80R + L. rhamnosus CLR2)*  vs placebo, Screened 2151, 1679 excluded (recent abx, vanco or metro) | 472 randomized, 437 done (8% attrition) Adults (>18 yrs old) on 3-14 days antibiotics (77% b-lactams)  In 8 Canadian hospitals 3/2006-10/07. CANADA | 5 x 10^10^/d  fermented **milk** | within 48 hours of abx—to 5 days post-abx (mean 12 days, ranging 29-40 days) F/up: 21 d APP | **AAD** (>2 days diarr) 47/216 **(21.8%,)** p=0.067  **Duration** AAD= 0.67 + 20 d p=0.04  **AE**: 33.3%  ns | **AAD** 65/221 **(29.4%)**  Duration AAD= 1.2 + 3.2d  **AE**: 34.4% | **Sampalis** J & Dylewski J  2010  Arch Med Sci |

**AAD ---page 9**

**AAD—page 10**

|  | **Probiotic** | **Population** | **Daily dose** | **Duration** | **AAD in Probiotic** | **AAD in controls** | **Reference** |
| --- | --- | --- | --- | --- | --- | --- | --- |
| *+* | *S boulardii* + 14 day triple therapy (Amox+Clarthirtho+Lansoprazole)  vs Controls (triple therapy only) | N=223 Hp+   randomized adults  KOREA | dose nr  form nr | 2 weeks  F/up:  4 weeks by ^13^C-urea test  ITT | **AAD**: 32/107 (29.9%)* P=0.041  **erad**: 73/107 (**68.2**%) p=0.905 NS | AAD: 50/116 (43.1%)  erad: 80/116 (69.0%) | **Lee** JY 2011 J Gastro & Hepatol Mtg Abstract. 2011; 26(S5):257 Asian Pacific Digestive Week, 1-4 October 2011, SUNTEC Singapore |
| *-* | *L. acidophilus LA-5 +  Bifido. lactis Bb-12* vs 2 control groups | 88 *H pylori*+ outpatient adults (18-65 yrs old), asymptomatic, given triple therapy at wk 5 GERMANY | 2 x 10^9^/d  fruity **yogurt** | 8 weeks  F/up: 0 | **AAD**: 5/30 (16.7%), ns  against either controls | AAD heat killed yogurt 2/29 (7%) or milk control (8/29, 27%) | **de Vrese** M 2011  J Diary Res |
| *-* | *S. boulardii* CNCM I-745  (Biocodex )  vs placebo | Hospitalized adults (>50 yrs) at Lucco Hosp in Italy, 4/2009-7/2010. Of 562 eligible, only 275 (49% randomized), 204 done , 71 (26**%** attrition), not ITT  ITALY | 1 x 10^10^/d   lost 35 in SB group and 36 in placebo (~1/2 due to death, but ~1/2 due to lost to follow-up or stopped txt – | during antibiotics and 7 more days  F/up: 12 wks | **AAD** 16/106 (15.1%, NS, mean onset 36 days. Chi=0.14, p=0.70 mean diarrhea **duration**= 2.5 d CDI 3/106 (2.8%), p=0.84 Died 12.7% ns | AAD 13/98 (13.3%) mean onset= 16 days power= 4% Duration: =3 days CDI 2/98 (2%) Power= 3% Died 15.6% | **Pozzoni** P & Riva A 2012 Am J Gastro  Notes: no data on compliance  26% attrition  -no comparison with other AAD studies. |

| *+/-* | **Probiotic** | **Population** | **Daily dose** | **Duration** | **AAD in Probiotic** | **AAD in controls** | **Reference** |
| --- | --- | --- | --- | --- | --- | --- | --- |
| *-* | *S. boulardii* (Lab Biocodex) All had triple therapy (Amox+clarithro + omeprazole)  for 14 days | 100 Hp+ adults with peptic ulcers, One hospital 0% attrition CHINA | ~5 x 10^9^/d vs no txt control  **Sachet** | 14 days   F/up:   1 yr  ITT | **AAD** 3/50 (6%) NS **Hp-:** 42/50 (84.4%)* p=0.04 all AE : 8/50 (16%)* p<0.001 | AAD: 8/50 (16%) p=0.2  Hp-: 32/50 (64.4%)all AE: 34/50 (68%) | **Chu** Y  2012 African J Pharmacy & Pharmacology |
| *-* | "PY" Probiotic yogurt. Strains not defined- emailed author. emailed author. He replied yogurt was: *Lactobacillus acidophilus* La5 + *Bifido. bifidum* Bb12.  vs non-probiotic yogurt control (blinded)  vs no yogurt control (open) | 102 Hp + adults symptomatic, [18-85 yrs old] 88 done (14% attrition)  Both double blinded controls and open (no txt) controls   All had triple therapy (Amox, Clarithro, Pantoprazaole for 7 days)  IRAN | nr cfu/day in paper  response in author email on dose:  ~2 x10^6^/d  300 mg/d  **yogurt** | 7 days  F/up: 4 weeks  APP analysis | **AAD**: 7/31 (22.6%) ns   Hp erad: 19/31 (61%) NS Any AE: 20/31 (64%) ns | Non-probiotic yogurt:   AAD: 8/31 (25.8%) p=0.77 Hp-: 20/31 (64.5%) p=0.79 Any AE: 21 (68%) p=0.79  No yogurt control: AAD: 8/26 (30.8%) p=0.48  Hp-: 19/26 (73.1%) p=0.35 Any AE: 22 (85%)p=0.13 | **Mirzaee** V 2012 Iranian Red Crescent Med J  data from author in email |
| *+* | *S. boulardii* vs open control   All got TT (AmoxClarithOmep) x 14 days. | n=82  Hp+ children peptic ulcers (n=33) or chronic gastritis (n=49)  CHINA | 250 mg/d  cfu/d nr | 2 wks  F/up 4 wks | **AAD**: 5/41 (12.2%)* p<0.05 **AE:** 5/41 12.2%* less | AAD: 13/41 (31.7%) Open cntrl: AE: 13/41 (31.7%) | **Zhang** Y 2012 J Clin Pediat  *[In Chinese]* |

**AAD page 11**

**AAD page 12**

| ***+-*** |  | **Population** | **Dose/day** | **Duration** | **AAD in SB** | **Controls** | **Reference** |
| --- | --- | --- | --- | --- | --- | --- | --- |
| *+* | *S. boulardii* lyo vs 'no treatment' controls | 333 children (6 mon-14 yrs old) Resp infections, inpatients, 283 done, (50% attrition) CHINA | 1 x 10^10^/d  **powder** | for 2 weeks   F/up: 2 wks | **AAD** (11/139, 7.9%) p<0.001 | AAD (42/144, 29.2%) | **Shan** L 2013 Bene Micro |
| *-* | *S. boulardii* vs  nothing as controls June-October 2012 St Louis Hosp open design | 140 children (6 mon-18 yrs). 136 done (3% attrition), in or outpatients, IV or oral abx  PHILLIPINES | 500 mg/d  1 x 10^10^ /d  form nr | duration of antibiotic F/up: not specifically stated ~ within 2 weeks of abx | **AAD**: 11/66 (16.7%) p=0.39 duration= 2.45 + 0.7 days, p=0.03 | **AAD**: 16/68 (23.5%) duration= 3.06 + 0.68 days | **Casem** RAO 2013 Phili Infect Dis Soc Proceed Journal 2013;14:70-76 dose from author |
| *+* | “Yomogi” *S. boulardii* All had triple txt Amox-Clarythro- lam x 14 days vs nothing | 160 *H. pylori* + adults with gastritis at 1 hospital IRAN | 1 x 10^10^ /d **capsules** | 2 wks  F/up: 8 wks ITT | **AAD**: 27/80 (33.8%) p=0.04 by end of week 10 | Open controls: 58/80 (72.5%) | **Zojaji** H 2013 Gastro & Hepatol |
| *-* | *L. rhamnosus* GG  vs placebo | 59 Hp+ symptomatic adults (>18 yrs old) 0% attrition  All had triple therapy: Amox/Clarith/Omep (7 days) Single blinded (outcome assessor) VENEZUELA | 1.2 x 10^10^ per day  **"Vial"** | 2 weeks  F/up: 0  ITT | **AAD**: 4/29 13.8% ns p=0.73 **Any AE**: 10/29 (34.4%) ns | AAD: 6/30 (20%) Any AE: 10/30 (33.3%) | **Padilla** Ruiz M 2013 Rev Gastroent Peru translated [in Spanish] |
| *+* | *S. boulardii* CNCM I-745 "UltraLevure" vs no txt **contro Single blinded** (Patients unaware of other txt arm) | Adults with dyspepsia. Of 125 screened, 70 enrolled (aged 18-75 yrs old at one Greek hospital) 60 done (14% attrition) Both groups got triple therapy ( amoxicillin (2 g/d), clarithromycin (1g/d), & omeoprazole (40 mg/d) For 14 days.  GREECE | 300 mg/d  6 x 10^6^/d  **Capsule** [50 mg with 10^6^ cfu/cap] | 14 days  F/up: 6 wks  ITT | **AAD**: 1 (2.8%)*  p=0.026 **Hp-:**  30/36 (83.4%)*, p=0.034 | **AAD**:  7 (20.6%) **Hp-:**  control 20/34 (58.8%) | **Kyriakos** N  2013  Hosp Chronicles |

**AAD- page 13**

| **+/-** | **Probiotic** | **Population** | **Daily**  **dose** | **Duration** | **AAD in Probiotic** | **AAD**  **in controls** | **Reference** |
| --- | --- | --- | --- | --- | --- | --- | --- |
| *-* | Mix of *L. acid.* La5 + *Bifido. lactis* Bb12  vs placebo | N=396 adults (18-70 yrs old) given 7 days of abx for URTI outpatients  at 4 hospitals INDIA | Dose nr in abstract | 14 days  F/up: nr in abstract | **AAD** (21/198) 10.8% p=0.2 | AAD (31/198) 15.6% | **Chatterjee** S 2013 J Asso Physician India |
| *-* | *S. boulardii* vs open control   All got TT (AmoxClarithOmep) x 14 days. | n=60  Hp+ children with chronic gastritis  CHINA | 500 mg/d | 2 wks  F/up: 4 wks | **AAD**: 0/30 (0%) p=0.11 **Erad:** 27/30 (90%)* p<0.05 **AE**: sign less but data in Chinese | Open cntrl:  AAD: 4/30 (13.3%)**Erad** 20/30 (66.7%) AE: raw data? | **Zhang** H  2013 Med J Chinese People Health *[In Chinese]* |
| - | *L. acidophilus La-5 + Bifido. bifidum Bb-12* vs no txt control.  All had triple: [Amox or Metro] + Clarith + Omeprazole (14 days) | 100 Hp+ children symptomatic one site – a GI clinic 88 done (12% attrition)  Jan 2009-June 2010 CHINA | if **<**5 yrs old:  1 x10^8^/d  if > 5 yrs old: 2.1 x 10^8^/d  **Sachets** | 6 wks  F/up: none!”  APP | **AAD**: 3/43 (7%) p=0.7 ns  **Hp-:**  36/43 (83.7%)* *X^2^*=4.3 p=0.04 **AE**: 5/43 (11.6%) p=0.07 | AAD: 5/45 (11.1%) Hp-: 29/45 (64.4%)  AE: 12/45 (26.7%) | **Wang** YH 2014  WJ Microbiol Biotech  [data supplied by author email]  not funded |
| + | *S. boulardii "*Bioflor" vs no txt control  All had triple (Amox/Clarith/ Omeproz)  (14 days) | 240 children Hp+ (5-11 yrs old) with gastritis, ulcers or inflammation 0% attrition  CHINA | 500 mg/d  1 x 10^10^  **Capsules** | 14 days  F/up:  4 wks  ITT | **AAD**: 27/120 (22.5%)* p=0.008   **Hp**-: 102/120 (85%) ns  p=0.07  **AE**: nr | AAD: 47/120 (39.1%)  Hp-: 91/120 (75.8%) | **Zhao** HM 2014  Zhongguo Dang Dai Erke Za Zhi  [in Chinese] |

| **+/-** | **Probiotic** | **Population** | **Daily dose** | **Duration** | **AAD in Probiotic** | **AAD in controls** | **Reference** |
| --- | --- | --- | --- | --- | --- | --- | --- |
| *+* | *L. casei* defensis DN114001 "Actimel" (Danone) vs   *L. casei* Shirota "Yakult" (Yakult) | 60 inpatients, adults at 1 hospital, mostly (80%) resp and GU infections, mixed types of abx 60% ampicillin or cephalosp)  No attrition reported  GERMANY | "Actimel": 2 x 10^10^/d  **drink** vs "Yakult" 2 x 10^10^/d **drink** | duration abx (mean 6 days)  F/up: 0 ITT | L. casei DN114001  **AAD** 2/30 (6.7%) p=0.02 | *L. casei* Shirota  AAD 10/30 (33.3%) | **Dietrich** CG 2014 World J Gastroenter |
| *+* | *S. boulardii* CNCM I-745 vs. no treatment controls  All had triple erad therapy:  AmoxClarrithOmeprazole or Metro ClarOmp if allergic to Amox x 14 days | N=205 enrolled, Hp+ children (22 months-16 yrs) n=194 done (5.4% attrition) CHINA | 1 x 10^10^/d 500 mg/d  **sachets** | 14 days  F/up: 4 wks  ^13^C urea breath test in subgroup of 42 kids>12 yrs old | **AAD**: 12/102 (11.8%) p=0.004  **Any AE**: nr  Compliance to std ther 100% p=0.03 | AAD: 26/92 (28.3%)    Complaince: 86/92 (93.4%) | **Bin** Z  2015 Ped Gastroentero, Hepatol & Nutrition |
| *-* | *“Lacidofil” Lacto. helveticus R52 + Lact. rhamnosus R11*  vs placebo | Phase 2 safety n=160 healthly adults  All received Amox-clavu for 1 week CANADA | 8 x 10^9^/day  **capsule** | 2 weeks  F/up:7 wks | **# AAD:** 19/76 (**25%**) p=0.3 ITT AAD on Abx: 8/77 (10%) ns Post-Abx: 12/77 (16%) ns p=-.32 **duration of diarrhea=** 2.7 + 0.4 days p=0.04  AE 9(12%) | #AAD: 23/70 (33%)  ITT AAD on Abx: 9/78 (11%)  Post-Abx: 17/78 (22%) duration of diarrhea= 3.7 + 0.4 days AE 20 (28%) | **Evans** M 2016 Br J Nutr |

**AAD page 14**

**AAD-page 15**

|  | **Probiotic** | **Population** | **Daily**  **dose** | **Duration** | **AAD in Probiotic** | **AAD**  **in controls** | **Reference** |
| --- | --- | --- | --- | --- | --- | --- | --- |
| *-* | **"SacBo Trial"** *Saccharo. boulardii* [Perenterol Forte®] HANSEN CBS5926 vs placebo [Manufactured by UCB Brussels, Belgium] due to low enrollment, RCT stopped prematurely at interim analysis  Low rate of AAD explained by authors: 1) ‘young’ pop enrolled (mean 60 yrs), 2) any abx included, not just high risk patients 3) high attrition | Adults (mean age: 60 Sb and 56 placebo yrs) inpatients at 15 hospitals in Germany (07/2010-10/2012) receiving systemtic antibiotics *(80% beta-lactams*) Screened 2444 enrolled N=477 enrolled, **292** done (39% attrition) Phase 3 trial GERMANY | 1.8 x 10^10^/d  **capsules**  Attrition high (n=185 dropped due to incomplete diaries) | duration of antibiotic (mean 8 days) plus 7 days  Max. txt was set at 8 weeks.  F/up:  6 weeks   ITT and PP | Sb  **AAD** ITT: 21/246 (8.5%) P=0.6 p=0.26 by episode [21 episodes] **3% power** Onset AAD=18.4 d   **AE:** 18/245 (7.3%)  Time in study same: 44.1 + 22.5 days, p=0.26 | placebo **AAD** ITT: 17/231 (7.4%)  [19 episodes of AAD] (2 had 2 episodes) Onset AAD=18.9 d   AE: 12/222 (5.4%)  time in study: 44 + 22 days | **Ehrhardt** S 2016 Open Forum Infect Dis   Poor study conduct due to: 1) high attrition rate due to incomplete diaries;  2) 13 patients did not receive correct study drug assignment,  3) #episodes used not #patients with AAD 4) low power due to early termination of study 5) survival curve not provided |
| *+* | *S. boulardii* CNCM I-745 vs open, no intervention control. Randomized | n=163 older inpatient adults on broad-spectrum antibiotics Jan 2014-Dec 2015 **CHINA** | 1 g/day (1 x 10^10^ cfu/d) **capsule** | for 21 days  F/up: not in abstract | **AAD**: 12/81 (14.8%) * P<0.05 **Duration**: 3.0 + 1.1 days* | **AAD**: 23/82 (28.0%)  **Duration**5**.**7 + 1.8 days | **Zhang** DM 2017 Zhonghua Nei Ke Za Zhi [in Chinese] |

## Clostridium difficile infections (CDI)- Primary prevention

CDI are due to a spore-forming gram positive anaerobe (*C. difficile*) and diagnosis was based on detection of *C. difficile* toxins along with new onset of symptomatic diarrhea not due to medications or other chronic GI conditions.

| ***+/-*** | **Probiotic** | **Population** | **Daily dose** | **Duration** | **CDI in Probiotic** | **CDI in Controls** | **Reference** |
| --- | --- | --- | --- | --- | --- | --- | --- |
| ***-*** | *S. boulardii* CNCM I-745 vs placebo  1 hosp 41% single abx., 36% cephalo | 318 enrolled, inpatients adults  180 done (43% attrition) [64 ineligible, 74 dropped] USA | 2 x 10^10^  ^within 48 hrs^  ^1 g^  **capsules** | duration + 14 days  F/up: Mean of 17 days  APP | **CDI**: 3/116 (2.6%)  p=0.13 *26.5% power*  *Of 48 CD toxin+, 3/32 (9.4%) CDI* | placebo: 5/64 (7.8%)    *Of 48 toxin +, 5/16 (31%) CDI p=0.07* | **Surawicz** C  1989  Gastroentero  Prevention of AAD study |
| ***-*** | *S. boulardii* CNCM I-745  vs placebo  4 hosp | 208 enrolled, 193 eligible all inpatients adults on beta-lactam abx, 18% single antibiotics  129 done (38% attrition)  USA | 3 x 10^10^  within 72 hrs 1 g/d  **capsules** | duration + 3 days  7 wk f/up  APP | **CDI** 3/97 (3.1%) ns p=0.72 *2.6% power*  but only 133 were tested for CD Of 10 toxin+, 3/10 (30%) CDI, 7 carriers | CDI: 4/96 (4.2%)  Of 14 toxin +, 4/14 were CDI and 10 were carriers, p=0.46 ns | **McFarland** L 1995  AJG  Prevention of b-lactam AAD study |
| ***-*** | *S. boulardii* CNCM I-745   vs placebo  1 UK hosp | 72 enrolled, 69 done (4.2% attrition), **elderly** (>65 yrs old) inpatients on abx (most multiple abx, nr types)  U.K. | 4.5 x 10^9^  within 24 hrs  226 mg/d  **capsules** | duration (mean 7 d),  no f/up  APP | **CDI**:  5/33 (15%) p=0.47 ns *7.2% power* | CDI: 3/36 (8.3%) | **Lewis** SJ  1998 J Infect  Prevention of AAD study |

**Primary CDI prevention-page 2**

| ***+/-*** | **Probiotic** | **Population** | **Daily**  **dose** | **Duration** | **CDI in Probiotic** | **CDI in Controls** | **Reference** |
| --- | --- | --- | --- | --- | --- | --- | --- |
| ***-*** | *L. rhamnosus* GG vs placebo | 167 children (2 wks-13 yrs old) enrolled, 119 done (29% attrition due to long f/up) 66% ampic. outpatients with URT Infections (26%) or otitis media (74%) FINLAND | 4 x 10^10^ **capsules** | duration abx. (mean=7-10 days)   F/up: 3 months   APP | **CDI** 1/61 (1.6%) NS p=1.0 *10% power* | 1/58 (1.7%) | **Arvola** T  1999  Pediatrics |
| ***-*** | *L. rhamnosus GG* vs placebo 1 hosp | 302 enrolled, 267 done (11.6% attrition) Adult inpatients 18-93 yrs old 07/06-10/99 69% b-lactams USA | 2 x 10^10^  within 24 hrs  **capsules** | 2 wks  F/up: 1 wk APP | **CDI** 2/133 (1.5%) ns p=1.0  *power 2.7%* | placebo 3/134 (2.2%) | **Thomas** MR 2001  Mayo Clinic Procedures  (Prevention of AAD) |
| ***-*** | *S. boulardii* CNCM I-745 vs placebo wafers  3 Polish Ped hosp and 2 outpatient clinics enrolled from 11/2002-05/2004  41% cephalo. | 269 enrolled children 6 mon-14 yrs old inpatient (27%) & outpatients on abx for resp (68%) or otitis media (29%) 246 done (8.5% attrition) POLAND | 1 x 10^10^  500 mg  within 24 hrs  **wafers** | duration (mean 7 days),  no f/up  APP | **CDI** 3/119 (2.5%) ns p=0.09 *35.9% power* | placebo 10/127 (7.9%) | **Kotowska** M 2005  APT  Prevent AAD study |

**Primary prevention of CDI-page 3**

| ***+/-*** | **Probiotic** | **Population** | **Daily**  **dose** | **Duration** | **CDI in Probiotic** | **CDI in Controls** | **Reference** |
| --- | --- | --- | --- | --- | --- | --- | --- |
| ***-*** | *S. boulardii*  CNCM I-745 vs no txt controls  All received triple therapy (Amox/Clarith/Omepr x 2wks) 9 hospitals | 389 outpatient adults Hp+ with PUD. 376 done (3.3% attrition)  TURKEY | 1 x 10^10^/d  1 g/d  **capsules** | duration abx (mean=2 weeks)  F/up: 4 weeks  ITT | **CDI**: 0/204 (0%)  p=0.48 *3.3% power  only those with diarrhea tested for Cdiff (n=5)* | open controls: 1/185 (0.5%)   *only 11 with diarrhea tested for Cdiff* | **Duman** DG 2005 Euro J Gastro Hepat |
| ***-*** | *S. boulardii*  CNCM I-745 vs placebo  1 hosp | 151 inpatient adults (25-50 yrs old), 151 done, on abx (83% beta-lactams) TURKEY | 1 x 10^10^  **capsules** | duration of abx  F/up: 4 wks  ITT | **CDI**: 0/73 (0%) ns p=0.50,   *9.1% power* | Placebo CDI: 2/78 (2.6%) | **Can** M  2006 Med Sci Monitor |
| ***-*** | "Bio-K+" *L. acido* CL1285 + *L. casei LBC80R + L rhamnosus CLR2* fermented milk  vs placebo milk 1 hosp Quebec | 89 enrolled, inpatient adults on varied Abx  (59% quinolones) for 92% resp infections 0% attrition 09/03-05/04  CANADA | 5 x 10^10^ within 48 hrs   **milk** | duration of abx (mean 7-8 days)  F/up: 21 days  ITT | **CDI** 1/44 (2.3%) P=0.06 TREND *44.2% power*  AE n=21 (48%) ns | placebo milk 7/45 (15.6%)  AE: n=20 (44%) | **Beausoleil** M 2007 CJG Prevention of AAD study |
| ***-*** | *S. boulardii*  CNCM I-745 (Reflor) in Turkey BCX, vs nothing; both on triple therapy | 124 adults with *H. pylori* + dyspepsia | 2 x 10^10^  (1g/d) | 2 wks with  6 wk f-up | **CD** toxin+: 6/62(9.7%) | Control CD toxin +: 8/62 (12.9%) | **Cindoruk** M 2007 not blinded |

**Primary prevention of CDI-page 4**

| ***+/-*** | **Probiotic** | **Population** | **Daily**  **dose** | **Duration** | **CDI in Probiotic** | **CDI in Controls** | **Reference** |
| --- | --- | --- | --- | --- | --- | --- | --- |
| ***pos*** | *L. casei* DN 114001 ‘immunitass’ + [*L. bulgaricus + Strept. thermophilus]*  (Actimel drink) vs placebo milkshake,  3 London hospitals | 135 inpatient adults > 50 yrs old 109 done (19% attrition)  11/02-01/05 61% single abx, 66% on high risk antibiotics (amoxicillin or cephalosporins) for 49% respiratory infections U.K. | 2 x 10^10^  within 48 hrs (200 g)  **drink** | duration of abx + 1 week  F/up: 4 wk   APP | **CDI**: 0/56 (0%)  p=0.001  *80.8% power* 1^o^ outcome was 7/57 (12.3%) AAD | placebo 9/53 (17%)    1^o^ outcome was 19/56 (33.9%) AAD | **Hickson** M 2007  BMJ  Prevention AAD study  Estimated cost of preventing one case of CDI with probiotic: $120.00. |
| Letters to BMJ on the Hickson 2007 paper. Criticisms: (1) Of 1760 screened, only 112 completed (only 7% potential target population). [**Wilcox** MH 2007 BMJ Letters] (2) No high risk Antibiotics given. [**Hillyard** T 2007 BMJ letters] Actually only recent prior use of high risk antibiotics were excluded in 4 weeks prior to enrollment. During the study 66% did receive high risk antibiotics. | | | | | | | **see above** |
| ***-*** | *L rhamnosus* GG vs placebo  PILOT study | 189 adults (>18 yrs old) inpatients receiving mixed types of antibiotics: b-lactams, pen, ceph (50%), single/multiple CANADA | 4 x 10^10^  **capsules** (within 72 hrs of abx) Primary outcome: prevent CDI ITT | duration abx x=14 days,   30 days of f/up | **CDI**  4/95 (4.2%) NS p=0.37 *9.2% power* No difference in AE (2.1%) | placebo (7/94, 7.4%)    AE (4.2%) | **Miller** M  2008**A** 48^th^ ICAAC mtg abstract K-4200.  Study #1  *Same abstract, but 2 pilot studies reported* |
| ***-*** | *L rhamnosus* GG vs placebo  [triple of the LGG dose of previous and larger study] stopped early | 316 adults (>18 yrs old) inpatients receiving mixed types of antibiotics: ceph (~50%), quinolones, IV vanco, metro  69% single abx. CANADA | 1.2 x 10^11^ **capsules**  Primary outcome: prevent CDI  ITT | duration abx: x=14 days, (within 72 hrs of abx. Start)  30 days of f/up | **CDI** 2/157 (1.3%) ns P=0.25 *11.2% power*  AE: 2.5%, ns | placebo (0/159, 0%)    AE: 0% | **Miller** M  2008**B** 48^th^ ICAAC mtg abstract K-4200.  Study #2   *Same abstract, but 2 pilot studies reported* |

**Primary prevention of CDI-page 5**

| ***+/-*** | **Probiotic** | **Population** | **Daily dose** | **Duration** | **CDI in Probiotic** | **CDI in Controls** | **Reference** |
| --- | --- | --- | --- | --- | --- | --- | --- |
| ***-*** | *S. boulardi*   CNCM I-745 vs placebo  1 hospital 88% resp. infections | 86 adults on amoxicillin, outpatients, 82 done (4.6% attrition) CHILE | 1 x 10^10^ (500 mg/d) **capsules** | 12 days  F/up: 9 days   ITT | **CDI** n=0/41 (0%) p=1.0 | CDI in placebo:  0/45 (0%) | **Bravo** MV 2008  Rev Med Chi original in Spanish-translated |
| ***low dose pos*** | BioK+ (L. *acidophilus* CL1285 + *L. casei* LBC80R *+ L. rhamnosus* CLR2) vs placebo  1 Shanghai hosp | 255 inpatients adults (50-70 yrs) Jan-March 2009. Randomized to low dose (9% attrition) or high dose (7% attrition) vs controls  47% resp infections Mixed abx: cephl (37-41%), pen or clinda CHINA | **Low** dose (5 x 10^10^/day)  ^within 36 hours^  **capsule** | During abx (3-14 days) plus 5 days    F/up: 21 days  ITT | **CDI**: Low dose: 8/85 (9.4%) p=0.03  *power 64.0%* | Placebo 20/84 (23.8%) | **Gao** XW 2010 AJG   [also **Musher** D 2009 IDSA abstract] |
| ***high dose pos*** |  |  | **High** dose (1 x 10^11^/d)  ^within 36 hours^ **capsule** |  | High dose 1/86 (1.2%)  p=0.002 power *99.2%* |  |  |
| ***-*** | BioK+ (L. *acidophilus* CL1285 + *L. casei* LBC80R *+ L. rhamnosus* CLR2) vs placebo fermented milks *Screened 2151, 1679 excluded (recent abx, vanco or metro)*  8 hospitals | 472 adults randomized, 437 done (7.4% attrition)  57% inpatients [35 excluded cuz too short on abx or diarrh at enrollment]  on 3-14 days antibiotics (78% b-lactams), 39% had resp infec, 3/2006-10/07 CANADA | 5 x 10^10^/d  ^within 48 hours^ **milk** | duration + 5 days post-abx (mean 12 days txt) , range of txt: 29-40 days.  F/up: 21 d  APP | **CDI** 1/216 (0.5%), p=0.40 ns *12.5% power  Only total of 46 screened for C diff!* Sub-group of CD+:  CDI   1/16 (6.2%) ns p=0.64 | n=221  4/221 (1.8%)  Sub-group of CD+ only: CDI:  4/30 (13.3%) | **Sampalis** J, Psaradellis E et al. 2010 Arch Med Sci  **incorrectly cited** Dylewski J 2010 Arch Med Sci 2010  also as: Ki 2008 Mtg abstract Am Coll Gast Oct 2008 |
| *+* | *L rhamnosus GG* vs placebo *Not all on antibiotics-only 50 (34%) with VAP (author email) mixed abx (nr types)* | 146 adult inpatients in ICU on mechanical ventilation (high risk), 138 done, 5% attrition USA | 4 x 10^9^ **capsule** | duration of intubation (mean 15 days)  F/up: none APP | **CDI** 4/68 (5.8%) p=0.02 *power 52.9%*  # on abx: n=33 | CDI 13/70  (18.6%)  # on abx: n=17 | **Morrow** LE 2010 Am J Resp Crit Care Med |

| *+/-* | **Probiotic** | **Population** | **Daily**  **dose** | **Duration** | **CDI in Probiotic** | **CDI in Controls** | **Reference** |
| --- | --- | --- | --- | --- | --- | --- | --- |
| ***-*** | *S. boulardii* ( CNCM I-745 Biocodex)  vs placebo  1 hospital 69% single abx | 275 inpatient adults on variety of antibiotics, 204 done (26% attrition) ITALY | 1 x 10^10^ [within 2 days of antibiotic] **capsules** | while on abx +7 days  F/up: 12 wk  APP | **CDI** 3/106 (2.8%) ns p=1.0 *power 3%* | CDI placebo 2/98 (2%) | **Pozzoni** P 2012 Amer J Gastro  primary outcome AAD |
| ***+*** | *S. boulardii* lyo CNCM I-745 vs ‘no treatment’ controls with acute lower resp infections,  52% on ceph 1 hospital | 333 inpatient children (6 mon-14 yrs old) with resp infections, 283 done [50 dropped] (15% attrition) CHINA | 1 x 10^10^/d  [500 mg/d]  **powder** | duration of abx: mean=2 weeks   F/up: 2 wks APP | **CDI** (1/139, 0.72%) p=0.04 *51.9% power* | open controls: CDI (8/144, 5.6%) | **Shan** L 2013  Beneficial Microbes |
| ***-*** | *L. casei* defensis DN114001 "Actimel" (Danone) vs *L. casei* Shirota "Yakult" (Yakult) | 60 inpatients, adults at 1 hospital  mostly (80%) resp and GU infections,   mixed types of abx 60% ampicillin or cephalosp)  No attrition reported GERMANY | "actimel":  2 x 10^10^/d  **drink**  vs  "yakult" 2 x 10^10^/d  **drink** | duration abx (mean 6 days)  F/up: none   ITT | *L. casei* DN114001 **CDI**: 0/30 (0%) p=0.24 *power 21.3%* | *L. casei* Shirota control: **CDI**: 3/30 (10%) | **Dietrich** CG 2014 World J Gastroenter |

**Primary prevention of CDI-page 6**

**Primary prevention of CDI-page 7**

| *+/-* | **Probiotic** | **Population** | **Daily dose** | **Duration** | **CDI in Probiotic** | **CDI in Controls** | **Reference** |
| --- | --- | --- | --- | --- | --- | --- | --- |
| *-* | **"SacBo Trial"** *Saccharo. boulardii* [Perenterol Forte®] HANSEN CBS5926 *(aka*  CNCM I-745) vs placebo [Manufactured by UCB Brussels, Belgium] due to low enrollment, RCT stopped prematurely at interim analysis | Adults (mean age: 60 Sb and 56 placebo yrs) inpatients at 15 hospitals in Germany (07/2010-10/2012) receiving systematic antibiotics *(80% beta-lactams*) Screened 2444 enrolled N=477 enrolled, 292 done (39% attrition) Phase 3 trial GERMANY | 1.8 x 10^10^/d  **capsules**  [within 2 days of antibiotic]  Attrition high (n=185 dropped due to incomplete diaries**)** | duration of antibiotic (mean 8 days) plus 7 days  Max. txt was set at 8 weeks.  F/up:  6 weeks   ITT and PP | Sb   **CDI**: 2/246 (0.8%) ns  **AE:** 18/245 (7.3%) | Placebo:  **CDI**: 2/231 (0.9%) low rate! AE: 12/222 (5.4%) | **Ehrhardt** S 2016 Open Forum Infect Dis   Poor study conduct due to: 1) high attrition rate due to incomplete diaries; 2) 13 patients did not receive correct study drug assignment, 3) low power due to early termination of study |
| *-* | *S. boulardii* CNCM I-745 vs open, no intervention control. Randomized | n=163 older inpatient adults on broad-spectrum antibiotics Jan 2014-Dec 2015 CHINA | 1 g/day (1 x 10^10^ cfu/d) **capsule** | 3 weeks  F/up: 9 weeks | (N=81)  **CDI**: 3/81 (3.7%) ns | (N=82)  **CDI**: 4/82 (4.9%) | **Zhang** DM 2017  Zhonghua Nei Ke Za Zhi [in Chinese] |

## Enteral feeding: prevention of diarrhea

Definition of outcome=days of diarrhea (score-based on >3 nonformed stools/day) while on enteral nutrition.

| **+/-** | **Probiotic** | **Population** | **No.** | **Dose** | **Duration** | **Outcome**  **in Probiotic** | **Oucome**  **in controls** | **Reference** |
| --- | --- | --- | --- | --- | --- | --- | --- | --- |
| + | *S.  boulardii* I-745 | adults in ICU | 40  ITT | 5 x 10^9^/d | 11-21 days | 34/389 (8.7%)* diarrhea days | 63/373 (16.9%) days | **Tempe** JD  1983  Sem Hop Paris |
| + | *S.  boulardii* I-745 | burnt adults  18-70 yrs | 20 enrolled, 18 done | 2 g/d  2 x 10^10^/d | 8-28 days | 3/204 (1.5%*) days | 19/208 (9.1%) days | **Schlotterer** M  1987  Nutr Clin Metabol |
| + | *S.  boulardii* I-745 | adults | 131 enrolled, 128 done | 2g/d  2 x 10^10^/d | 21 days | 91/650 (14%)* days | 134/705 (19%) days | **Bleichner** G  1997 Intens Care |

**Abbreviations**ICU=Intenstive Care Unit; *p<0.05

## Helicobacter pylori trials: prevention of adverse reactions

Outcome defined as any adverse reaction (nausea, diarrhea, abdominal pain, vomiting, etc.) associated with triple or quadruple treatments for the eradication of *H. pylori*.

| **+/-** | **Probiotic** | **Age of subjects (months)** | **No.in study** | **Dose/day** | **Duration** | **Incidence of any adverse reaction in probiotic** | **Any adverse reaction in controls** | | **Reference** |
| --- | --- | --- | --- | --- | --- | --- | --- | --- | --- |
| *+* | *Lactobacillus rhamnosus GG  "*GiFlorex*"* vs nothing.  All on triple therapy for 7 days: (claritho, **pantoprazole**, tinidazole) | Hp+ carriers Hospital staff, adults. May-July 1999, one site, 117 done (2.5% lost) ITALY | n=120 | 1.2 x 10^10^ **Sachet** | 14 days f/up:  6 wks | 26 (43%) p=0.04 | | 37 (62%) | **Armuzzi** A 2001 **A** Digestion |
| *+* | *Lactobacillus rhamnosus GG  "*GiFlorex*"* vs placebo **blinded** study All on triple therapy for 7 days (claritho, **rabeprazole**, tinidazole) | asymptom. Hp+ carriers Hospital staff, adults. Sept 1999 -Jan 2000 0% attrition ITALY | n=60 | 1.2 x 10^10^  **Sachet** | 14 days f/up:  6 wks | 12/30 (40%)* p=0.04 | | 20/30 (67%) | **Armuzzi** A 2001 **B** APT |
| *+* | **RCT with three txt arms:** *L. rhamnosus GG "*Giflorex" vs placebo  All triple therapy (7 days) ClaRanTin | adults asymptomatic 41 done  (5% attrition placebo, 0% LGG) ITALY | n=42 | 1.2 x 10^10^ **sachets** | 14 days  f/up: 5-7 wks | 3/21 (14%) p=0.004 | | 12/20 (60%) | **Cremonini** F 2002  AJG |
| *+* | *S boulardii "*Codex"vs placebo All triple therapy (7 days) ClaRanTin | adults  40 done (5% attrition in placebo, 9% Sb)  ITALY | n=42 | 1 x 10^10^  **sachets** | 14 days  f/up: 5-7 wks | 3/21 (14%) p=0.004 | | 12/20 (60%) |  |

**Prevention of AE due to *H. pylori* treatments-page 2**

| **+/-** | **Probiotic** | **Age of subjects (months)** | **No.in study** | **Dose/day** | **Duration** | **Incidence of any adverse reaction in probiotic** | **Any adverse reaction in controls** | | **Reference** |
| --- | --- | --- | --- | --- | --- | --- | --- | --- | --- |
| *-* | *SB "*Reflor*"* Randomized, OPEN trial (SB vs nothing) Triple txt (amox, clarithro, omeprazole) for 14 days | adults with peptic ulcers  (3% attrition) TURKEY | 384 enrolled, 376 done | 1 x 10^10^/d  **Capsules** | 2 wks,   Follow-up: 4 wks | 3 (1.5%) ns | | 3 (1.7%) | **Duman** DG 2005 Euro J Gastro & Hept |
| *+* | *L. acido + L. rhamnosus* vs  controls (triple therapy only) “Lacidofil” All got 10 days of triple therapy AmoxClarith +PPI | children (7-18 yrs old) with H. pylori dyspepsia or ulcers   POLAND | n=60 | 6 x 10^9^/d  **Capsules** | 20 days  F/up:  7 wks | 1/30 (3%) p=0.002* | | ]11/30 (38%) | **Plewinska** E  2006 Gastroenterol Pol translated in Polish  notes from author |
| *+* | *S. boulardii ("*Reflor", Biocodex) vs placebo. All got triple therapy (ACL) (14 days) | outpatients  TURKEY | n=124 0% attrition | 2 x 10^10^ /d  **Sachets** | 2 weeks  F/up:  6 wks | 14/62 (23%)* P<0.001 | | 37/62 (60%) | **Cindoruk** M 2007 Helicobacter |

|  | | | | | | | | |
| --- | --- | --- | --- | --- | --- | --- | --- | --- |
|  | P**revention of AE due to *H. pylori* treatments-page 3** | | | | | | | |
|  | **Probiotic** | **Age of subjects (months)** | **No.in study** | **Dose/day** | **Duration** | **Incidence of any adverse reaction in probiotic** | **Any adverse reaction in controls** | **Reference** |
| *+* | *"*Lacidofil*" L. helveticus R0052 +  L. rhamnosus* R0011  vs no txt control All got triple therapy (OmeAmoxClarith) for 7 days | adults gastritis or ulcers  UKRAINE | n=49 0% attrition | 8 x 10^9^ /d  **capsules** | 10 days  F/up: 4-6 wks | 1(4%)* p=0.049 | 6/24 (25%) | **Vdovychenko** V 2008 [Current Gastroenterol]  translated  [In Ukrainian]  [data from company] |
| *+* | *S boulardii* (Enterol, Biocodex) vs no txt control All had triple therapy (Omer or Eso [3 wks] & Amox/Clar) (7-10 days)  **Single blinded** (outcome assessor) | symptomatic children  3-18 yrs ROMANIA | n=90 | 1 x 10^10^  **Capsule** | 4 wks  fup: 4-6 wks | 4/48 (8%)* p=0.047 | 13/42 (31%) | **Hurduc** B  2009  Act Paed |
| *-* | *L. rhamnosus GG* vs placebo  all had triple therapy (amox and clarithr and Omeprazole) for 7 days | children asymptomatic  *Hp +* inpatients POLAND | n=83 66 done (20% attrition | 2x 10^9^  **Capsule** | 7 days  F/up: 6 wks | 18/35 (51%) ns  p=0.8 | 13/32 (41%) | **Szajewska** H 2009  JPGN |
| *-* | **2 txt arms:** *S. boulardii vs* vs no txt control.  All got triple therapy OAC (7 days)  **Single blinded** (UBT outcome assessor) | 991 adults Hp+  symptomatic (ulcer, gastritis)  SOUTH KOREA | n=991  932 done | 2.2 x 10^10^  **Capsule** | 4 wks F/up: 4 weeks | 48/330 (14.5%) p=0.12 | 63/331 (19%) | **Song** MJ  2010 Helicobacter |
| *+* | *S. boulardii +* **mucoprotective agent** (extract of *Aartemisia asiatica*) |  |  |  |  | 30/330 (9.1%) * p<0.001 |  |  |

**Prevention of AE due to *H. pylori* treatments-page 4**

| **+/-** | **Probiotic** | **Age of subjects (months)** | **No.in study** | **Dose/day** | **Duration** | **Incidence of any adverse reaction in probiotic** | **Any adverse reaction in controls** | | **Reference** |
| --- | --- | --- | --- | --- | --- | --- | --- | --- | --- |
| *+* | ***Concomitant*** *S. boulardii* and triple therapy x 7 days,  *controls*: triple therapy only x 7 days open (no placebo) TT: Amox + Clarith + rabeprazole for 7 days | 135 Hp+ adults  >18 yrs old active gastritis 9/2010-02/2012 CHINA | n=85 | 1.5 x 10^10^/d  **capsule** | 7 days  F/up: 4 wks | 16/41 (39%) p=0.02 | | 28/44 (63.6%) | **Gao** C, Xie R Ma T, Wu S. 2012 Chin J Gastroentero. I**n Chinese** |
| *+* | *S. boulardii* (Lab Biocodex) All had triple therapy (Amox+clarithro +  omeprazole)  for 14 days | Hp+ adults with peptic ulcers CHINA | n=100 | ~5 x 10^9^/d  **Sachet** | 14 days   F/up:   1 yr | 8/50 (16%)* p<0.001 No SAE | | 34/50 (68%) | **Chu** Y  2012 African J Pharmacy & Pharmacology |
| *+* | All got TT (AmoxClarithOmep) x 14 days. randomized to:  *S. boulardii* vs open control | Hp+ children peptic ulcers (n=33) or chronic gastritis (n=49) CHINA | n=82 | 250 mg/d | 2 wks  F/up 4 wks | 5/41 12.2%* | | 13/41 (31.7%) | **Zhang** Y 2012 J Clin Pediat  *[In Chinese]* |
| *-* | *L. rhamnosus* GG vs placebo  All had triple therapy: Amxo/Clarith/Omep  (7 days) **Single blinded** (outcome assessor) | adults (>18 yrs old) symptomatic  VENEZUELA | n=59 | 1.2 x 10^10^ per day  **"Vial"** | 2 weeks  F/up: none | 10/29 (34.4%) ns | | 10/30 (33.3%) | **Padilla** Ruiz M 2013 Rev Gastroent Peru translated  [in Spanish] |

## Necrotizing enterocolitis (NEC)- prevention

NEC outcome was new onset of Bell’s stage 2 or 3, but stage not reported in all studies.

| **+/-** | **Probiotic** | **Age of subjects (months)** | **No.in study** | **Dose/day** | **Duration** | **Incidence of NEC (Bell’s stage) in probiotic** | **NEC (stage) in controls** | | **Reference** |
| --- | --- | --- | --- | --- | --- | --- | --- | --- | --- |
| *-* | *L. rhamnosus GG* | preterm neonates (<33 wks or <1500 g) | 585 | 6 x 10^9^ | 48 d (until discharge) | NEC (nr) 4/295 (1.4%) ns  p=0.26 | | NEC (nr) 8/290 (2.7%) | **Dani** C  2002 Biol Neonate |
| *-* | *S boulardii* CNCM I-745 in formula vs control (maltodextrin) | pretermneonates(28-32 wks old) GREECE | 87 | 2 x 10^9^/d | 30 d | NEC (nr) 5/51 (9.8%) p=0.51 | | NEC (nr) 6/36 (16.7%) | **Costalos** C 2003 Early Human Devel |
| *+* | *Bifid. infantis, Strept. therm, Bifid bifidus “*ABC Dophilus” formula vs control formula | <1500 g  ISRAEL | 145 | 1 x 10^9^ | 36 wks | NEC(> 2): 1/72 (4%) p=0.01 died: 3/72 (4.2%) ns | | NEC(> 2): 10/73 (16.4%) died: 8/73 (11%) | **Bin-Nun** A 2005 J Pediat |
| *-* | *L. rhamnosus GG “Dicloflor”* | neonates (<1500g)  in NICU | 80 | 6 x 10^9^ | from day 3 of life to 6 wks | NEC (> 2) 1/39 (2.5%) p=0.62 | | NEC (> 2) 3/41 (7.3%) | **Manzoni** P 2006 Clin Infect Dis |
| *-* | *Bifido. lactis* Bb12 | Pre-term neonates (<37 wks) | 69 | 4.8 x 10^9^ | 21 d | NEC (> 2) 2/21 (9.5%) p=1.0 | | NEC (> 2) 1/17 (5.9%) | **Mohan** R 2006 J Clin Microbiol |
| *+* | *Bifido. bifidum NCDO1453+ L. acidophilus NCDO1748 “Infloran”* | low BW (<1500 g) neonates o <34 wks old TAIWAN | 434 | 2 x 10^9^/d | 6 wks | NEC(> 2): 4/217 (1.8%)* p=0.03 | | Control formula NEC(> 2): 14/217 (6.5%) | **Lin** HC 2008 Pediatrics |
| *+* | *L. casei subsp rhamnosus* GG vs placebo Both with bovine lactoferrin too | infants <1500 g ITALY | n=319 | 6 x 10^9^/d **formula** | 4 wks | NEC2: 0/151 (0%)* | | NEC2 10/168 (6%) | **Manzoni** P 2009 JAMA |

Abbreviations: NEC, necrotizing enterocolitis; NICU, neonatal intensive care unit; NS, not significant; VLBW, very low birth weight

**NEC- page 2**

| *+/-* | **Probiotic** | **Age of subjects (months)** | **No.in study** | **Daily dose** | **Duration** | **Incidence of NEC** | **NEC in Controls** | **Reference** |
| --- | --- | --- | --- | --- | --- | --- | --- | --- |
|  | *Bifido lactis* Bb12 vs placebo | Neonates (<30 wks or <1500g) VLBW | 183 | 1.2 x 10^10^/d | 6 weeks | NEC (nr) 2/93 (2.1%) ns | NEC (nr) 4/90 (4.4%) | **Mihatsch** WA 2010  Neonat |
|  | *L. reuteri* DSN 17938 “BioGaia AB” vs placebo | Neonates (<2000 g) in NICU | 750 | 1 x 10^8^ drops | until discharge | NEC (2) 9/372 (2.4%) p=0.23 NS | Placebo: NEC (2) 15/378 (4%) | **Rojas** MA 2012 Pediatrics |
| *-* | *S. boulardii*  CNCM I-745 in formula ("Reflor" France) vs control formula (no SB) Oct 2010-Nov 2011 Turkey | Premature infants (<1500 g & age <32 wks old) | n=208 | 1 x 10^9^  cfu/kg/d | duration hosp stay | NEC (> 2) 7/104 (6.7%) NS sepsis: 28.8% ns No AE | NEC (> 2) 7/104 (6.7%) sepsis: 23% | **Serce** O  2013 Early Human Develop |
| *+* | ‘ProPerms trial’ “ABC Dophilus”*: Bifido. infantis, B. lactis, Strept. thermos* vs placebo powder | < 32 wks & <1500 g | 1,099 ITT | 1 x 10^9^ (1.5 g) powder | 40 wks of life or until discharge | NEC(> 2): 11/548 (2%), p=0.03 Died 4.9% NS | NEC(> 2): 24/551 (4.4%) Died 5.1% | **Jacobs** SE 2013 Pediatrics |
| *-* | *S. boulardii*  CNCM I-745 in formula ("Reflor" France) vs control formula (no SB)  March-Nov 2011 Turkey | Premature infants <1500 g & age <32 wks old | n=278 randomized n=271 done (3% attrition) | 5 x 10^9^/d  iven in breast milk or formula | duration hosp stay  F/up: none | NEC (nr): 4.4% of 135, NS Sepsis: 35%, p=0.03died: 3.7% ns sepsis significant!: 34.8%, p=0.03 | NEC (nr): 5.1% of 136.  Sepsis: 48%  Died: 3.6% sepsis 47.8% | **Demirel** G 2013 Acta Paediatr |

**NEC page 3**

|  | **Probiotic** | **Age of subjects (months)** | **No.in study** | **Daily dose** | **Duration** | **Incidence of NEC** | **NEC in Controls** | **Reference** |
| --- | --- | --- | --- | --- | --- | --- | --- | --- |
| *+* | **3 txt arms:** *(1) L. rhamnosus* GG + bovine lactoferrin (LGG-BL)  (2) control: just lactoferrin (BL) vs (3) placebo | VLBW infants in ICU   multisite   Italy and New Zealand | 743 | 6 x 10^9^ | Until discharge or day 30 | **NEC** (LGG-BL):  0/238 (0%), p<0.001 | **NEC** (bovine lactoferrin control): 5/247 (2%), p=0.06 **Placebo**: NEC 14/258 (5.4%) | **Manzoni** P 2014 Early Hum Dev |
| *-* | “Infloran” *L. acidophilus* nr + *Bifido. bifidum* nr | neonates <34 weeks & BW <1500 g Thailand | 60 | 2 x 10^9^ capsule in liquid | 6 wks | NEC(> 2): 1/31 (3.2%) ns AE: none | NEC(> 2): 1/29 (3.4%) | **Saengtawesin** V  2014 J Med Asso Thai |
| *-* | *L. reuteri* DSN17938 “BioGaia AB” vs placebo | neonates | n=400 | 1 x 10^8^ per day formula | duration hosp stay | NEC(> 2): 8/200 (4%) NS | NEC (> 2): 10/200 (5%) | **Oncel** MY 2014 Arch Dis Child Fetal Neona Edu |
| *-* | *L. reuteri*  DSN17938 “BioGaia AB” vs nystatin | neonates | n=300 | 1 x 10^8^ per day formula | duration hosp stay  F/up: none | NEC(> 2) 7/150 (4.7%) ns Sepsis: 7.3% p=0.03 | NEC:(> 2) 9/150 (6%)  Sepsis: 14.7% | **Oncel** MY 2015 J Mat Fet Neona Med |
| *+* | *L. reuteri protectis*  DSM17938 “BioGaia” vs control | preterm infants 28-32 wks or 1400-1800 g BW IRAN | N=60 | 4 x 10^7^ /d | until first enteral feed | **NEC** (nr): 2/30 (6.7%)* **Sepsis:** 4 (13.3%)* **Died:** 1 (3.3%) ns | **NEC** (nr): 11/30 (36.7%) **Sepsis:** 10 (33.4%) **Died:** 2 (6.7%) | **Nouri**  Shadkam M 2015 Iran J Neonat |

## Nosocomial Infections-prevention

Nosocomial infections defined as new onset of disease>48 hours after admission

| **+/-** | **Probiotic** | **Given to** | **Dose and Duration** | **Outcome and Effect in Probiotic group^*^** | **Effect in Control group** | **Reference** |
| --- | --- | --- | --- | --- | --- | --- |
| - | *L rhamnosus GG* | 61 pediatric patients admitted to pediatric ICU  USA | 1 x 10^10^  **capsule** duration of stay  F/up: none | **Any nosocomial infection:** 6/31 (19.3%) ns (developed at least one nosocomial infection)   Types: bacteremia  (2, 6%) pneumonia  (2, 6%) bronchitis  (5, 16%) UTI (2, 6%)  No AE | Any noso infection: 3/30 (10%)   Types: bacteremia (n=3, 13%)   No AE | **Honeycutt** TCB  2007  Ped Crit Care Med |
| *+* | *L. rhamnosus* GG vs fermented milk control  **(*did not count AAD cases [diarrhea+abx and neg stool pathogens]   no data on AAD freq.** | 742 hospitalized children (>1 yr old, mean 10 yrs old) CROATIA | 10^9^/d in 100ml milk  duration of stay,  followed for 7 days post-discharge | **Nosocomial GI disease**: 19/376 (5.1%*) [excluded cases of AAD]   Noso Resp infections 8/376 (2.1%*)  LOS=5 days NS | Noso GI: 44/366 (12%)  & Noso Resp tract infections (mostly Upper)-20/366 (5.5%)  LOS=4 days | **Hojsak** I  2010  Pediatrics  data from author |

AAD, antibiotic-associated diarrhea; GI, gastrointestinal; LOS, length of stay, UTI, urinary tract infections

## Respiratory Infections – prevention

Respiratory infections defined as any of a variety of new onset upper respiratory infections, variety of outcomes reported by different trials.

|  | **Probiotic** | **Age of subjects (months)** | | **No.in study** | **Dose/day** | **Duration** | **Incidence of respiratory tract infection in:** | | **Reference** |
| --- | --- | --- | --- | --- | --- | --- | --- | --- | --- |
|  | | | | | | | **Probiotic** | **Control** |  |
| *-* | *L. rhamnosus* GG in **milk** vs control milk | | Healthy children  (1-6 yrs old) in 18 day cares | 571 | 1-2 x 10^8^ /d | 7 months | 21 days of resp infections in group, NS | normal milk 22 days | **Hatakka** K 2001 Brit Med J |
| + | *L. casei* DN114001 milk "Dan Active" | | Elderly volunteers (>60 yrs old) ITALY | 360 | cfu/d nr  2 bottles per day | 3 wks | n=180  20% reduction in all "winter infections” duration lower  (7.0 + 3.2 d, p=0.02) | n=180  longer duration 8.7 + 3.7 days | **Turchet** P 2003 J Nutri Health Aging |
| *-* | *L rhamnosus* GG | | pediatric patients admitted to ped ICU; followed for different types of nosocomial infections | 61 | 1 x 10^10^ | duration of stay | total **respiratory infections:** 7/31 (22.6% NS also: pneumonia (2, 6%),  bronchitis (5, 16%) | Resp infections:  0/30 (0%) | **Honeycutt** TCB  2007 Ped Crit Care Med |
| *-* | *Actimel  (L. casei DN114-001 [L. bulgaricus, Strept thermo]* | | Children  (2-5 yr old) | 187 | 1 x 10^10^/d | 12 months | Time free from rhinitis: mean 6.2 mon NS  Mean # episodes= 3.2* | Placebo drink. Time free:5.1 mon  Mean #: 4.8 | **Giovannini** M 2007 Ped Res |
| *-* | Actimel (*L. casei* DN114001) vs control dairy product | | elderly (>70 yrs old), healthy volunteers | pilot of 86, then confirm with n=222 | 2 bottles of 100 g/d, 7 wks (pilot) or 13 wks (confirm) | got Influ B vaccine  4 wks post-txt | Significantly higher antibody titers to Influ B (~90 GMT); H1N1 NS; H3N2 ns | ~60 GMT for Influ B | **Boge** T  2009  Vaccine |

**Resp Infections-page 2**

|  | **Probiotic** | **Age of subjects (months)** | **No.in study** | **Dose/day** | **Duration** | **Incidence of disease** | **controls** | **Reference** |
| --- | --- | --- | --- | --- | --- | --- | --- | --- |
| *+* | *L. rhamnosus* GG  vs  fermented milk control | hospitalized children (>1 yr old, mean 10 yrs old)   CROATIA | 742 | 10^9^/d in 100ml  **milk** | duration of stay,  followed for 7 days post-discharge | **Noso Resp infections** 8/376 (2.1%*) Noso GI disease 19/376 (5.1%*) (excluded cases of AAD) | Noso Resp tract infections (mostly Upper)-20/366 (5.5%)  Noso GI: 44/366 (12%) | **Hojsak** I 2010 Pediatrics |
| + | *L. casei* DN114001 (Actimel) drink with *L. delbrueckii* and *Strept thermo* | Elderly volunteers over 3 months of winter, >70 yrs old | 1072 | 2 x 10^10^/d vs non-fermented dairy product | 3 months with  1 mon f-up | Duration of all resp infections= 6.5 d.  cumulative incidence all resp infection=7 days, p=0.009 | Duration resp infections=8 d, p=0.008.  Cumulative incidence=8 d | **Guillemard** E  2010 Br J Nutrition |
| + | *L. rhamnosus* GG vs inert inulin-based placebo | ventilator dependent hosp patients in ICU  Not all on abx, from author email: | 146 enrolled, 138 done,  5% attrition | 4 x 10^9^/d | mean = 15 days | **Pneumon**: 9%, p=0.007  CDI (5.8%, p=0.02) | Pnemon 40%   CDI 18.6% | **Morrow** LE 2010  Am J Resp Crit Care Med |
| + | *L. rhamnosus* GG (ATCC 53103)  vs  vs placebo | preterm neonates  at Turku U Hosp,   FINLAND | n=94,  n=68 completed 1 yr f/up:.  (28% attrition) | 1 x 10^9^/d for Days 1-30 then 2 x 10^9^/d day 31-60 | betweendays 3-60 of life.  F/up:  1 yr | Mean # resp infections: 1.2 + 1.6, p<0.001 | Mean # Resp in  placebo:  2.5 + 2.0 | **Luoto** R 2014  J Aller Clin Immunol |
| - | *L. rhamnosus* GG (living) vs placebo | Prevent rhinovirus in healthy adults (18-65 yrs old) USA | 60 enrolled, 59 done  (1.7% attrition) | 1 x 10^9^/ d (~100 ml)  Fruit **JUICE** | 6 wks  F/up: none | Got colds 14/19 (74%) NS, P=0.20 **AE**: 9/19 (47.4%)* | colds: 18/20 (90%) **AE**: 7/20 (35%) | **Kumpu** M 2015 Bene Microbes |

## Surgical Infections-preventive

Defined as any type of new onset infection occurring after day of surgery within follow-up period.

|  | **Probiotic** | **Age of subjects (months)** | **No.in study** | **Daily dose** | **Duation** | **Incidence of post-surgery infections** | | **Reference** |
| --- | --- | --- | --- | --- | --- | --- | --- | --- |
| *-* | *L. plantarum 299 (*living*)* + oat fiber vs control (standard decontam treatment) | **Adults** having varied types of major abdominal surgery (liver, gastric, colon, pancreas) | 105 enroll,  90 done (14% attrition)    10/1997-03/1999 | 2 x 10^9^  **Formula** | 4 days post-surgery  F/up: 6 days | Living: # infections 3/30 (10%) NS  AE: 7/30 (23%) NS *only sign if both living and dead pooled: 6/60(10%) p=0.02* | Std control: 9/30 (30%) AE 10(33%) | **Rayes** N 2002 **A** Nutrition |
| *+* | *L. plantarum 299 (*living*)* + oat fiber vs control (standard decontamin treatment) | Adults  post-liver transplantation  Outcome #post-op infections | 105 enroll,  95 done   (9.5% attrition)   10/1997-10/1999 | 2 x 10^9^  **Formula** | 12 days post surgery  F/up: none | **Living** Synbiotic: 4/31 (13% )* infection vs std txt P<0.05  AE: 19% | Control-std bowel prep: 15/32 (48%)   AE 25% | **Rayes** N 2002 **B** Transplantation |
| *-* | *L. plantarum 299v* (“Proviva”), open trial  no placebo, just untreated controls | adults patients undergoing major abd surgery | 129 enrolled | ~2.5 x 10^9^ cfu/d | Md 9 d pre-op and Md 5 days post-op | In 64,  bacterial translocation 12%, p=0.82, **sepsis** (13%, ns) or gastric colonization with enterics (11%) ns | In 65 non-probiotic group, BT (12%), **sepsis** (15%) or gastric colonization (17%) | **McNaught** CE  2002  Gut |

**Surgical infections- page 2**

|  | **Probiotic** | **Age of subjects (months)** | **No.in study** | **Daily dose** | **Duration** | **Incidence of post-surgery infections in probiotic** | **Infections in control** | **Reference** |
| --- | --- | --- | --- | --- | --- | --- | --- | --- |
| **_+_** | *“Synbiotic 2000” ® (Pediacoccus pentosaceus  5-33:3 + Leuconostoc mesenteroides 77:1 + Lacto. paracasei ssp. Paracasei F19 + L. plantarum 2362)* and 4 fibers | adults scheduled for liver transplant | 66 enrolled  n=66 done | 2 x 10^10^ /d and 20g/d fiber  sachet | Started on day of surgery and then for 2 weeks  F/up: 2 weeks | 1/33 (3%), P<0.05 | Placebo + 4 fibers only 16/33 (48%) | **Rayes** N 2005 Am J Transplant-ation |
| **+** | *“Synbiotic 2000” ® (Pediacoccus pentosaceus  5-33:3 + Leuconostoc mesenteroides 77:1 + Lacto. paracasei ssp. Paracasei F19 + L. plantarum 2362)* and 4 fibers | Multiple trauma patients at 5 ICUs GREECE | 65 | 4 x 10^11^ per day (12 g/d of 4 fibers: inulin, oat bran, pectin, starch) | 15 days   F/up: until ICU discharge | 22/35 (63%), p=0.01 | 27/30 (90%) | **Kotzampassi** H 2006 Langenbecks Arch Surg |
| **+** | *“Synbiotic 2000” ® (Pediacoccus pentosaceus  5-33:3 + Leuconostoc mesenteroides 77:1 + Lacto. paracasei ssp. Paracasei F19 + L. plantarum 2362)* and 4 fibers | adults with pancreatic resection | 89 enrolled 80 done | 10 ^10^ and 10g fiber | 9 days | 5/40 (12.5%)* | 16/40 (40%) | **Rayes** N 2007  Annals of Surgery |
| **+** | *“Synbiotic 2000” ® (Pediacoccus pentosaceus  5-33:3 + Leuconostoc mesenteroides 77:1 + Lacto. paracasei ssp. Paracasei F19 + L. plantarum 2362)* and 4 fibers | multiple injured adults in surgical ICU. All had enteral feed   SLOVENIA | 132 enrolled n=113 done | 4 x 10^10^/d and 10 g fibers/d | nr | 5/26 (19%) p=0.03 | 46/87 (53%) | **Spindler**-Vesel A 2007 J Parent Enter Nutri |

**Post-surgical infections- page 3**

|  | **Probiotic** | **Age of subjects (months)** | **No.in study** | **Daily dose** | **Duration** | **Incidence of post-surgery infections in probiotic** | **Infections in control** | **Reference** |
| --- | --- | --- | --- | --- | --- | --- | --- | --- |
| **_-_** | *“Synbiotic 2000” ® (Pediacoccus pentosaceus  5-33:3 + Leuconostoc mesenteroides 77:1 + Lacto. paracasei ssp. Paracasei F19 + L. plantarum 2362)* and 4 fibers | Adults with hepatecomy surgery  All had enteral feeding  GERMANY | 19 | 2 x 10^10^  sachet | 10 days | 3/9 (33%) ns | 4 fiber only controls: 2/10 (20%) | **Rayes** N 2012 Benef Microbes |

## Travellers’ diarrhea- Prevention

Travellers’ Diarrhea defined as: diarrhea (>3 loose stools/day for 2 days or > 5 loose stools/48 hours) occurring during travel, not present at trip origin and not due to pre-existing chronic intestinal conditions.

|  | |  |  |  |  |  | **Frequency of traveler’s diarrhea** | |  | |
| --- | --- | --- | --- | --- | --- | --- | --- | --- | --- | --- |
| **+/-** | | **Probiotic** | **Number in study** | **Population and destination** | **Dose/day** | **Duration** | **Probiotic** | **Placebo** | **Reference** |  |
| ***+*** | | *S. boulardii* Hansen CBS5926 [now CNCM I-745] vs placebo | 832 | Austrian tourists to hot climates mean age=42 yrs old  TURKEY | Low dose 2 x 10^9^ 250 mg **capsules** | 21 d  F/up: none | 143/426 (34%)* | 173/406 (43%) | **Kollaritsch** HH 1989 Travel Med Intrl |  |
| ***+*** | | *S. boulardii* Hansen CBS5926 [now CNCM I-745] vs placebo | 805 | Austrian tourists to hot climates mean age=42 yrs old  TURKEY | High dose 5 x 10^9^ 500 mg **capsules** | 21 d  F/up: none | 127/399 (31.8%) * *best in North Africa* | 173/406 (43%) | **Kollaritsch** HH 1989 Travel Med Intrl |  |
| ***-*** | *Lactobacillus rhamnosus GG* vs placebo | 820 enrolled (402 in probiotic and 418 control), 756 done (8% attrition) | Finnish tourists to Turkey (10-80 yrs old), mean age=44 yrs  FINLAND. | 2 x 10^9^ **sachets** | 7-14 d duration of trip  **92% compliance** | 153/373 (41%) ns  p=0.06 *but p=0.04 for Alanya destination* | 178/383 (46.5%) | **Oksanen** PJ  1990 Ann Med |  |  |

**Prevention of TD continued-page 2**

|  |  | |  |  | | |  |  | **Frequency of traveler’s diarrhea** | | | |  |
| --- | --- | --- | --- | --- | --- | --- | --- | --- | --- | --- | --- | --- | --- |
|  | **Probiotic** | | **Number in study** | **Population and destination** | | | **Dose/day** | **Duration** | **Probiotic** | **Placebo** | | | **Reference** |
| ***+*** | *S. boulardii CNCM I-745* vs placebo  High dose arm | 695 [used common placebo] (392 dropped, 56% attrition) | | | Austrian tourists to hot climates  mean age=44-45 yr TURKEY | High dose: 2 x 10^10^ 1 g/day  **capsules** | | 21 d  **61% compliance** | **TD**: 87/303 (29%) * p=0.005 **AE**: 62/695 (8.5%) | | TD: 141/361 (39%) **AE**: 69/727 (9.5%) | **Kollaritsch** HH  1993 Fort. der Med [in German} | |
| ***+*** | *S. boulardii* CNCM I-745 “Perenterol” vs anti-diarrheal med (ethacridine-lactate | 60 enrolled, 43 done, (28% attrition) | | | Tourists adults with TD, Tunisia | 1 x 10^10^ **capsules** | | 5 days | 2.1 days mean diarrhea* | | 1.4 days diarrhea | **Bruns** R  1995 [in German] | |
| ***+*** | *Lactobacillus rhamnosus GG* vs placebo | 400 enrolled, 245 done (39% attrition) | | | NYC American tourists (17-80 yrs old, mean 50 yrs old) to various locations USA | 2 x 10^9^ **capsules** | | 7-21 d duration of trip  **64% compliance** | 5/126 (3.9%) p=0.05 *  n=2 abd cramps | | 9/119 (7.4%) | **Hilton** E  1997 J Travel Med | |

## Urinary Tract Infections- prevention

UTI outcome defined as new onset of urinary tract infection or recurrence of new UTI.

|  |  |  |  |  |  | **Frequency of rUTI** | |  | |
| --- | --- | --- | --- | --- | --- | --- | --- | --- | --- |
|  | **Probiotic** | **Number**  **in study** | **Population and destination** | **Dose/**  **day** | **Duration** | **Probiotic** | **Placebo** | **Reference** |  |
| **-** | *L. rhamnosus* GG “Gefilus” vs.  no juice control | 150 137 done (8.7% attrition) | outpatient women *E. coli* UTI mean age ~30 yrs old  **Finland** | 4 x 10^10^ in 100 ml  5d/wk  **drink** | 1 year  F/up: 1 yrs | UTI: 19/49 (39%) NS | control 18/27 (36%) | **Kontiokari** T 2001 BMJ |  |
| **-** | *L. rhamnosus* GG vs milk control | 585 | preterm infants **ITALY** | 6 x 10^9^ milk | 7 days F/up: none | n=290 3.4% ns | n=295 5.8% | **Dani** C  2002 Biol Neon |  |
| **-** | *L. rhamnosus GG*- vs placebo outcome was any nosocomial infection, reported types sub-groups | 61 | children in PICU (1-216 mon old) **USA** | 1 x 10^10^ /d **capsule** | until  discharge  F/up: none | (n=30) UTI: 2/30 (6%) NS | (n=31) UTI 0/31 (0%) | **Honeycutt** TCB 2007;8:452-8 Ped Crit Care Med |  |

# Treatment

## Adult Acute Diarrhea-treatment

Adult acute diarrhea defined as acute onset of diarrhea (>3 loose/watery stools/day for >2 days). Outcomes included percent reporting ‘cure’ (diarrhea resolved) or mean days of diarrhea.

| +/- | **Probiotic** | **Study population** | **Probiotic treatment** | **Probiotic group (cure or duration diarrhea)** | **Control group  (cure or duration diarrhea)** | **Reference** |
| --- | --- | --- | --- | --- | --- | --- |
| *+* | *Enterococcus faecium* SF68 | 78 Swiss adults with acute diarrhea | 2.2 x 10^8^/d for 7 days | 37/40 (92.5%)* cured | 33/38 (86.8%) | **Wunderlich** PF  1989 J Int Med Res |
| *-* | *Enterococcus faecium* SF68 | 183 adults in Bangladesh with either cholera or *E. coli* diarrhea | 4 x 10^9^/d  for 3 days | 3d (cholera) and 1 d (*E. coli*)  ns | 3 d (cholera) and 1 d (*E. coli*) | **Mitra** AK 1990 Gastroenterol |
| *+* | *S. boulardii* CNCM I-745 | 92 German outpatient adults with acute diarrhea | 1 x 10^10^  300-600 mg/d for 8 days | -17.2* diarrhea score reduction | -13.6 | **Hochter** W 1990 Munch Med Wschr |
| *+* | *S. boulardii* CNCM I-745 | 35 French AIDS patients with chronic diarrhea | 6 x 10^10^  3 g/d for 7 days | 11/18 (61%)* cured | 2/17 (12%) | **Saint-Marc** T 1992 Sem Hop Paris |
| *+* | *Enterococcus faecium* SF68 | 185 adults with diarrhea in Belgium | 4.5 x 10 ^8^ /d  for 5 days | 1.7 + 0.6d *  duration of diarrhea | 2.8 + 0.9 | **Buydens** P 1996 Scan J Gastroenterol |
| *-* | *S. boulardii* CNCM I-745 | 10 adults mixed etiologies, cross-over study | 2 x 10^10^  7 days | 3.8 stools/d by end, ns | 3.9 stools/d | **Attar** A  1999 Gastroenterol |
| *+* | *S. boulardii* CNCM I-745 + Metro vs Metro only control also see amboebiasis | 57 enrolled, n=54 done, adults with *E. histolytic* amoebic dysentery | 1.5 x 10^10^ [750 mg/d] for 10 days 4 week f/up | 100% cure* duration diarrhea:  12 + 3.7 d, p<0.001 | Metro only: 5/27 (19%) cured,  duration  48 + 18.5 d | **Mansour- ghanaei** F 2003 World J Gast |

**Treatment of acute adult diarrhea –page 2**

| **+/-** | **Probiotic** | **Study population** | **Probiotic treatment** | **Probiotic group (cure or duration diarrhea)** | **Control group (cure or duration diarrhea)** | **Reference** |
| --- | --- | --- | --- | --- | --- | --- |
| *+* | *S. boulardii* CNCM I-745 + metro vs metro only control | 65 adults with giardiasis | 1 x 10^10^  for 10 days | 100% cure* | 6/35 (17%) | **Besirbellioglu** BA 2006 J Infect Dis |
| *-* | *“Zhengchangsheng” Bacillus licheniformis* [Korea] *vs “Bioflor” (S. boulardii* [Biocodex]  2 probiotics compared, no control/placebo used | n=158 adults (20-75 yrs old) with diarrhea, n=151 done (4.4% attrition)  **KOREA** | Bacillus: 6 caps/d (250 mg/cap) or 1.6 g/d no cfu/d  Sboulardii: 1 g/d, 4 caps/d  For 5 days, F/up: none  **capsules** | (n=80) Sb **cured** by day 3: 756/80 (95%) p=0.33  **Duration diarrhea**: 3.2 + 1.0 d, p=0.70 | (n=78) B. lich. cured: 71/78 (91%)  Duration: 3.15 + 1.1 d | **Heo** J 2014 Intestinal Research |

*P<0.05

## Clostridium difficile infections (CDI): Treatment

Outcome was a new recurrence of CDI episode with 2 months of prior resolution of CDI.

**Treatment trials with primary outcomes for CDI**:

|  | **Population** | **Probiotic** | **Dose and duration of treatments** | **CDI recurred in Probiotic Group** | **CDI recurred in Control Group** | **Reference** |
| --- | --- | --- | --- | --- | --- | --- |
| + | adults with CDI. Outcome is CDI recurrence All got either vanco or metro | *S. boulardii* CNCM I-745  vs placebo  capsules | 2 x 10^10^/d for 4 wks follow-up 4 wks | overall: 15/57 (26.3%) p=0.05 Recurrent CDI patients: 9/26 (34.6%) p=0.04 but initial CDI: 19.3% ns p=0.86 | overall: 30/67 (44.8%)  Recurrent: 22/34 (65%) Initial: 24.2% | **McFarland** LV 1994 JAMA |
| + | n=170 adults, all got vanco (2 g/d or 500 g/d) or metro (1 g/d) for 10 days  adults with recurrent CDI | *S. boulardii* CNCM I-745  vs placebo  capsules | 2 x 10^10^/d for 4 wks follow-up 4 wks | **Recurred**: only in high dose vanco: 3/18 (16.7%) p=0.05 ns for low dose vanc and metro  No AE | high dose vanco & placebo: 7/14 (50%) | **Surawicz** CM 2000 Clin Infect Dis |
| - | 25 inpatient & outpatient adults on vancomycin or metronidazole (7-10 days), **recurrent** (n=9) and **initial** (n=16) CDI attrition nr USA | *Lactobacillus rhamnosus* GG in yogurt  vs placebo | cfu/d nr  **yogurt**  3 weeks   F/up: 4 weeks  ITT | **Recurred** 4/11 (36.4%) ns p=1.0 5.7% power  **Initial** CDI: 0/6 recurred, p=0.25 **RCDI**:  4/5 (80%) recurred p=0.52 | 5/14 (35.7%)  Initial CDI: 3/10 (30%) recurred  RCDI:  2/4 (50%) recurred | **Pochapin** MB 2000;95(1):S11-S13. Amer J Gastro  Meeting abstract only data from author  terminated early due to poor enrollment |
| - | 15 adults on vanco or metro (80% on metro)  doses Abx nr but mean duration Vanco or Metro (18 days), **Recurrent CDI only**, enrolled over 9 months 0% attrition Missouri USA | *L. rhamnosus GG* and inulin | 5.6 x 10^11^  *(2.8 x 10^11^ per 40 mg capsule bid)* **capsule** duration of antibiotics (median 18 days) + 21 days= total 39 days F/up: 21 days | **Recurred**:  3/8 (37.5%) p=0.57, ns 5.3% power  More bloating (25%) & gas (37.5%) | Placebo + inulin recurred: 1/7 (14.3%) | **Lawrence** SJ  2005 J Med Microbiol  poor enrollment stopped early, low power |

## Pediatric Colic-Treatment

|  | **Probiotic** | **Age of subjects (months)** | **No. in study** | **Dose given per day** | **Duration of treatment (days)** | **Percent cured or duration of diarrhea (days) in  probiotic \| controls** | | **Reference** |
| --- | --- | --- | --- | --- | --- | --- | --- | --- |
| *+* | *L. reuteri strain 55730 vs simethicone*  outcome is "crying" | infantile colic, breast-fed  21-90 days old | n=90 colicky infants, 83 done | 1 x 10^8^ live /d | 28 days | 39/41 (95%) responded (less crying), p<0.001  Crying time: 51 min/d, p<0.001 | 3/42 (7%)  Crying time: 145 min/d | **Savino** F 2007 Pediatrics |
| *+* | *L. reuteri* DSM 17938 vs placebo  Responders=50% reduction in crying | 2-16 weeks old | 50 infants with colic, 46 done, no AE | 1 x 10^8^/d | 21 days | Crying times:  Median= 35 min/d NS Responders: 24 (96%), p=0.04 | crying time: 90 min/d  Responder: 15 (71%) | **Savino** F 2010 Pediatrics |
| *+* | *L. reuteri* DSM 17938 vs placebo  Outcome: responders had >50% less crying | <5 months old with colic | N=80, mostly breast-fed | 1 x 10^8^/d | 21 days F/up: 1 wk | Med crying 52 min/day P<0.001  Responders 40/40 (100%) | Med crying 120 min/day  Responders 25/40 (62.5%) | **Szajewska** H 2013 J Pediatri |
| *+* | *L. reuteri* DSM 17938 vs placebo  Italy | 1-3 months old with colic | N=589 randomized |  | 90 days | Mean duration of crying= 38 min/d, p<0.01 | Crying: 71 min/d | **Indrio** F 2014 JAMA Pediat |

Outcome was mean or median time of infant crying/day

| **+/-** | **Probiotic** | **Number in study** | **Daily dose** | **Duration** | **Outcome in Probiotic** | **Controls**  **placebo** | **Reference** |
| --- | --- | --- | --- | --- | --- | --- | --- |
| *+* | *Activia yogurt (Bifido animalis* DN173010) + FOS vs control dessert | 266 adult women with functional constipation and 112 with normal stool function | 2 x 10^10^ (2 units of 10^8^ cfu/g) | 14 days  F/up: 0 | BM/week: in constipated:  x=6.1 + 2.7/wk p<0.001  Sign better on straining on defection and pain symptoms  in normal: NS | Control yogurt x=5 + 2.6/wk | **DePaula** JA  2008 Acta Gastroenterol Latinoam |
| *+* | *Activia yogurt (Bifido animalis* DN173010) vs acidified control milk with DEAD bacteria | 135 constipated women (25-65 yrs old), 126 done  (7% attrition)  CHINA | 1 x 10^10^ 100 g  **Milk/ yogurt** | 14 days  F/up: 0 | Living: BM/week:  4.1 + 1.7, p<0.05 | Dead:  2.6 + 1.0 | **Yang** YX 2008  World J Gastroenterol |
| *-* | *Activia yogurt (Bifido animalis* DN173010)  vs control milk (non-fermented milk) | 159 children (3-16 yrs old) with mean of 3 yrs Rome III constipation. 148 done (7% lost).  In Netherlands and Poland. | 8 x 10^9^ **yogurt** or **milk** | 3 wks   F/up: 0 | Living: cured  27/71 (38%) p=0.06 trend. What happened to 3 subjects? Not reported in paper.  Increase by 2.9 +3.2 BM/wk, p=0.5 NS | Cured in placebo: 17/72 (24%). [Missing 2 subjects]  Increase by 2.6 + 2.6 bm/wk No SAEs | **Tabbers** MM 2011**A** Pediatrics. Neg cuz? Low dose? Severe disease? Too short? No efficacy? |

## Constipation- treatment

Outcome of constipation trials was mean stools/week

## H. pylori treatment

**-page 1**

*H. pylori* clearance based on DOB <5 at 30 minutes is negative for *H. pylori* (excess labeled CO2)

| **+/-** | **Probiotic** | **Population** | **Dose** | **Duration** | **Probiotic** | **Controls** | **Reference** |
| --- | --- | --- | --- | --- | --- | --- | --- |
| *+ erad -AE AAD nr* | *L. acidophilus Lb* "Lacteol Fort" vs no txt control  All on **triple** txt (ACR x 7 days). | 120  Hp+ adults, dyspepsia 117 done (2% attrition) ITALY | 1.5 x 10^10^  **Capsules** | 10 days F/up: 6 wks  ITT | **Hp erad** 52/60 (87%) p=0.02  **AE**: 6 (10%) p=1.0 | **Hp-:** 42/60 (70%)  AE: 6 (10%), ns | **Canducci** F 2000 Alim Pharm & Therapeutics  origin of *L acido* LB strain (p1628) |
| *-erad AE nr AAD nr* | *L. acidophilus LB* culture supernatant vs no txt control  All had **double** txt: Amox (14 days) + Omeprazole (30d) | 84 consecutive adults dyspepsia or ulcers,  0% attrition ITALY | 2 x 10^10^/ day  **Capsules** | 14 days  F/up: 4-6 wks ITT | **Hp erad:** 30/47 (63.8%) ns  AE: nr AAD: nr | Hp -: 26/37 (70.3%) | **De Francesco** V 2000 Dig Liver Dis [letter] |
| *- erad- + AE + AAD* | *Lactobacillus rhamnosus GG  "*GiFlorex*"* vs nothing.  **open** study All on triple therapy for 7 days: ( claritho, **pantoprazole**, tinidazole) | 120 asymptom. Hp+ carriers Hospital staff, adults. May-July 1999, one site, 117 done (2.5% attrition) ITALY | 1.2 x 10^10^ **Sachet** | 14 days  F/up:  6 wks  ITT | **Hp-:** 48/60 (80%), p=0.6  **Any AE**: 26 (43%) p=0.04 **AAD**: 8/60 (13.2%) P<0.001 | control Hp-: 46/60 (76.6%)  Any AE: 37 (62%)  AAD: 29/60 (48.2) | **Armuzzi** A  2001 **A** Digestion |
| *- erad + AE + AAD* | *Lactobacillus rhamnosus GG  "*GiFlorex*"* vs placebo **blinded** study All on triple therapy for 7 days (claritho, **rabeprazole**, tinidazole) | 60 asymptom. Hp+ carriers Hospital staff, adults. Sept 1999 -Jan 2000 0% attrition ITALY | 1.2 x 10^10^  **Sachet** | 14 days  f/up:  6 wks  ITT | **Hp neg**: 25/30 (83.3%) p=1.0 **Any AE**: 12/30 (40%)* p=0.04 **AAD:** 1/30 (3.3%), p=0.01 **Nausea** 10%* | placebo: Hp-: 24/30 (80%)  Any AE: 20/30 (67%)  AAD: 8/30 (26.6%) Nausea 37% | **Armuzzi** A  2001 **B** Alim Pharm & Ther |

Abbreviations: AAD: antibiotic-associated diarrhea; AE, adverse events; Hp+, *H. pylori* positive; Hp-, *H. pylori* negative (eradicated); ITT, intent-to-treat analysis; nr, not reported; ns, not significant

|  | **Probiotic** | **Population** | **Dose** | **Duration** | **Probiotic** | **Controls** | **Ref** |
| --- | --- | --- | --- | --- | --- | --- | --- |
| *-*erad/ *-AAD +any AE* | **RCT with three txt arms:** *L. rhamnosus GG "*Giflorex" vs placebo  All triple therapy (7 days) ClaRanTin | 21 LGG 21 placebo  n=42 adults asymptomatic 41 done  (5% attrition placebo, 0% LGG)  ITALY+ | 1.2 x 10^10^ **sachets  double blinded** | 14 days  F/up: 5-7 wks  APP | **Hp eradicated:**16/21 (76%) ns p=1.0 **Any AE:** 3/21 (14%) p=0.004 **AAD:** 1/21 (5%), ns | **Hp eradicated:** 16/20 (80%) ns **Any AE:** 12/20 (60%)  **AAD:** 6/20 (30%) | **Cremonini** F  2002  Amer J Gastro |
| *-*erad/ ***+****AAD +any AE* | *S boulardii*  *"*Codex"  vs placebo   All triple therapy (7 days) ClaRanTin | 22 Sb 21 placebo  asymptomatic  n=42 adults  40 done (5% attrition in placebo, 9% Sb)  ITALY | 1 x 10^10^  **sachets   double blinded** | 14 days  F/up: 5-7 wks  APP | **Hp eradicated:**17/20 (81%) ns p=1.0 **Any AE:** 3/21 (14%) p=0.004 **AAD:** 1/21 (5%) p=0.045 | **Hp eradicated:** 16/20 (80%) ns **Any AE:** 12/20 (60%)  **AAD:** 6/20 (30%) | **Cremonini** F  2002  Amer J Gastro |
| + erad + AE +AAD | "AB Yogurt" *L. acidophilus La5 + Bifido. animalis subsp lactis Bb12* & 2 starters *(Strept therm + L. bulgaricus)* vs.  controls no txt All triple therapy  (7 days) ACL | 160 Hp+ adults  symptomatic  149 done,  (7% attrition) Jan-Dec 2001  TAIWAN | 1 x 10^10^/d  **yogurt** | 4 wks  f/up: 4-8 wks  ITT | **Hp-**: 73/80 (91%)* p=0.045 **Any AE:** 15/80 (18.8%)* p<0.001 **AAD**: 2/80 (2%)* p=0.03 More Bifido in stool | **Hp-**: 63/80 (78%)  **Any AE:** 53/80 (66%)   **AAD**:10/80 (12.5%) | **Sheu** BS 2002 APT  compliance to triple ther: sign: 67.5% probiotic* vs 43.8% controls |

**H pylori. page 2**

**Hp—page 3**

|  | **Probiotic** | **Population** | **Dose** | **Duration** | **Probiotic** | **Controls** | **Ref** |
| --- | --- | --- | --- | --- | --- | --- | --- |
| *- erad + AE  AAD nr* | *C. butyricum MIYAIRI 588* vs no txt controls  All got triple therapy AFO (7 days) f=furazolidone | 97 Hp+ symptomatic adults  0% attrition   CHINA | 120 mg/d,  1 x 10^7^ cfu/d   **Tablet** | 7 days  F/up:  4 wks  ITT | **Hp-:** 44/47 (94%) p=0.49 **Any AE:** 6/47 (12.8%) p=0.04 By chi^2^ **AAD**: nr | **Hp-:** 44/50 (88%)  Any AE: 15/50 (30%) **AAD**: nr | **Guo** JB 2004 Chin J Celiopathy [In Chinese, but English abstract] |
| *-erad -AAD AE: nr* | *Clost. butyricum MIYAIRI 588* vs no txt controls All on triple therapy  ACL (7 days) L=lansoprazole | 35 adults (41-64 yrs)  Hp+ ulcers 0% attrition outpatient clinic JAPAN | 3 x 10^7^ /d  3 tablets of 120 mg/tab **Tablets** | 2 wks  F/up: 6 wks  ITT | **Hp-:** 17/18 (94%) ns **AAD**: 1/18 (6%) p=0.60  no change in normal flora | **Hp-:** 13/17 (76%)  **Any AE**: nr  **AAD**: 2/17 (11.8%) | **Shimbo** I 2005 WJG  good summary of MIYAIRI strain (p. 7520) |

|  | **Probiotic** | **Population** | **Dose** | **Duration** | **Probiotic** | **Controls** | **Ref** |
| --- | --- | --- | --- | --- | --- | --- | --- |
| *-*erad  AE nr AAD nr | **Two treatment arms:** Open study *1)Lact acidophilus LB  "*Lactol Forte" only (**no triple therapy**)   vs control  triple therapy (AmoxClaritLanz x 8 days) | 120 children 5-12 yrs old, 91 completed (24% attrition)  asymptomatic   CHILE | 2 x 10^9^  **Capsules** | 8 wks  f/up:  none  APP | (n=46) DEAD  Eradication of Hp 3/46 (6.5%)  worse in probiotics  DOB*** post txt=33.4  No AAD or AE data | (n=45) triple therapy:  66% (30/45) eradication   p<0.001  DOB post triple=8.4  No AAD or AE data | **Gotteland** M 2005  Acta Paedia  *shows probiotic by itself not effective for Hp eradication*! |
| -erad AE nd | *2) S. boulardii lyo and inulin* (5 g inulin) only (**no triple therapy**) "Perenteryl"  vs 8 days of triple therapy (AmoxClaritLanz) | 119 children 5-12 yrs old, 95 completed (20% attrition)   asymptomatic  CHILE | 1 x 10^10^  500 mg  **Sachet** | 8 wks  f/up:  none APP | Living Sb (n=50) eradication of Hp was 6/51 (12%) worse in probiotics   DOB post txt =31.2  No AAD or AE data | (n=45) triple therapy:  66% (30/45) eradication   p<0.05  DOB post triple=8.4 | **Gotteland** M 2005 Acta Paedia   *shows probiotic by itself not effective for Hp eradication* |
| +erad +AE AAD nr | *L. acidophilus (helveticus) + L. rhamnosus* vs  controls (triple therapy only) “Lacidofil” All got 10 days of triple therapy AmoxClarith +PPI notes from author | 60 children (7-18 yrs old) with H. pylori dyspepsia or ulcers   POLAND | 6 x 10^9^/d  **Capsules** | 20 days  F/up:  7 wks  ITT | **Hp-:** 30/30 (100%)* P=0.011 **AE**: 1/30 (3%) p=0.002*  **AAD**: nr | **Hp-:** 23/30 (76.6%) **AE**: 11/30 (38%)   **AAD**: nr | **Plewinska** E  2006 Gastroenterol Pol translated [Abstract in Polish] |

**Hp---page 4**

**Hp—page 5**

|  | **Probiotic** | **No.** | **Dose** | **Duration** | **Probiotic** | **Controls** | **Reference** |
| --- | --- | --- | --- | --- | --- | --- | --- |
| + erad AE nd AAD neg (trend) | *"*AB Yogurt" *L acidophilus La5 + Bif. animalis subsp lactis Bb12*  [starters: *L. bulgaricus + Strept thermophilus*]. vs no txt control  All on quadruple therapy (AmoxMetroOmeBs) (7 days) | 138 failed triple Hp therapy with ulcers or gastritis,  129 done (6% attrition)  adults  TAIWAN | 400 ml/d  4 x 10^11^/d   **Yogurt**  single blinded (Hp assessor) | 4 wks  F/up:  6 wks & 3 months if neg at 6 wks.  ITT | **Hp-:** 59/69 (85%)* P=0.04 **Any AE:** patient level data nr **AAD:** 9/69 (8.7%)  p=0.053 Chi^2^ 43% Metro resistant 62% Clarithro resistance | **Hp-:** 49/69 (71%)  **Any AE:** nr  **AAD:** 18/69 (26%) | **Sheu** BS 2006 Am J Clin Nutri |
| + erad AE nr AAD nr | "Lacidofil" *Lactobacillus ~~acidophilus~~ helveticus R0052 + L. rhamnosus R0011)* 10 days triple therapy Amox + Clarith + Pantoprazole, PPI)  vs 4 triple therapy control groups:  **Control IA (Triple)**: 10 days of Amox + Clarith + pantoprazole (PPI)  **Control IB (Quadruple)**: 10 days of Tetracycline + Tinidazole +Bismuth salts + pantoprazole (PPI)  Two other control groups txt by antibiogram for their Hp strain (n=155) | 641 adults with peptic ulcers or gastritis and Hp+  (18-81 yrs old)  1999-2002  but 4 different control groups!  0% attrition  POLAND | 8 x 10^9^/d  4 capsulesper day  cfu/d not reported in paper  but 2 x 10^9^/cap  **Capsules** | 20 days  F/up:  10 days  ITT | **Hp-:** 51/53 (96%)* p=0.04 *X*^2^=4.2 BUT NOT CORRECT should be Fisher's exact test p=0.052 power 40% vs IA (same triple therapy) vs. Quad p<0.001*  No data on AE or AAD 49% resistance to Clarith & 59% to Metro found | did not use the other 2 control groups here.  **Hp-:**  **Control IA (triple):**  165/192 (85.9%)  **Control IB**: (**Quad)** 172/241 (71.4%)  p<0.001 IB vs control IA.  Quad less effective than triple therapy! | **Ziemniak** W 2006 J Physiol & Pharmacol  poor quality study: 1. poorly done randomization as group sizes not near equal  2. No adverse effects data  company emailed dose data |

**H pylori continues-- page 6**

| **+/-** | **Probiotic** | **No Hp+.** | **Dose** | **Duration** | **Probiotic** | **Controls** | **Reference** |
| --- | --- | --- | --- | --- | --- | --- | --- |
| *-*erad/ +distress *+ AAD + AE* | *S. boulardii ("*Reflor", Biocodex)  vs placebo.  All got triple therapy (ACL) (14 days) GDQ=Glasglow dyspepsia questionnaire | 124 adults with dyspepsia  0% attrition outpatients  TURKEY | 2 x 10^10^ /d  **Sachets** | 2 weeks  F/up:  6 wks   ITT | **Hp-** (44/62, 71%) p=0.3 **GDQ**=1.38 + 1.2* **Any AE**: 14/62 (23%)* P<0.001  **Epigastric distress** 9 (14.5%)*  **AAD:** 9/62 (14.5%) p=0.03 *X^2^* **CDI**: 2/62 (9.7%) ns | **Hp-** (37/62, 59.7%) NS GDQ=2.2 + 1.4 Any AE: 37/62 (60%)    Distress: 27 (43.5%)  **AAD:** 19/62 (30.6%) **CDI**: 8/62 (12.9%) ITT | **Cindoruk** M 2007  Helicobacter |
| -erad  AE nr AAD nr | "Lacidofil*" L. helveticus* R0052 + *L. rhamnosus* R0011  vs no txt control All had Amox+Clari+ Rabeprazole for 7 days randomized | 35 Hp+ adults duodenal ulcers 18-70 yrs old 0% lost  UKRAINE | 1.2 x 10^10^ per day   6 caps/d  **capsules** | 20 days  F/up: 4 wks  ITT | **Hp-:** 18/20 (90%) ns 4.5% power Restored **normal flora** n=15 (75%), p=0.08 **Dyspepsia** better in  (x=6 + 0.6 days) p<0.01 | Hp-: 13/15 (86.7%) Restored NF: 6 (40%) Dyspepsia better in (x=10 + 1 days)  No **AE** or **AAD** data | **Babak** O 2007 News of Pharmacy & Medicine  [translated: In Ukrainian] translated data from company email |
| *+erad + Any AE  AAD nr* | *"*Lacidofil*" L. helveticus R0052 +  L. rhamnosus* R0011  vs no txt control.  All got triple therapy (OmeAmoxClarith) for 7 days | 49 Hp+ adults gastritis or ulcers  0% attrition  UKRAINE | 8 x 10^9^ /d  4 caps /day  **capsules** | 10 days  F/up: 4-6 wks  ITT | **Hp-:** 24/25 (96%)* p=0.049  Healed peptic ulcer: 22 (88%) ns Any **AE** 1(4%)* p=0.049 **AAD**: nr | Hp-: 18/24 (75%)  Healed peptic ulcer 17 (70.8%) Any AE: 6/24 (25%) | **Vdovychenko** V 2008 [Current Gastroenterol]  translated  [In Ukrainian]  [data from company] |

**H pylori-page 7**

| **+/-** | **Probiotic** | **No Hp+.** | **Dose** | **Duration** | **Probiotic** | **Controls*** | **Reference** |
| --- | --- | --- | --- | --- | --- | --- | --- |
| *- erad AE nr* | *Clostridium butyricum* CBM588 vs no txt control All got triple therapy ACL for 7 days | 19 peptic ulcer  adults (32-71 yrs old) 0% attrition  JAPAN | 6 x 10^7^/d to  1.2 x 10^8^/d  **tablets** | 7 days  F/up:  none  ITT | (n=12)  **Hp-:** 11/12 (92%) ns | (n=7)  **Hp-:** 6/7 (87%) **Any AE:** no data | **Imase** K 2008  Microb Immuno |
| *-*erad/ *+all AE AAD nr* | *S boulardii* (Enterol, Biocodex) vs no txt control  All had triple therapy (Omer or Eso [3 wks] & Amox/Clar) (7-10 days)  **Single blinded** (outcome assessor) | 90 symptomatic children   3-18 yrs  dyspepsia  0% attrition   ROMANIA | 500 mg/d  1 x 10^10^  **Capsule** | 4 wks  fup: 4-6 wks  ITT | **Hp-:** 45/48 (93.3%) p=0.75  **all AE:** 4/48 (8%)* p=0.047  **AAD:** nr | Hp-: 34/42 (80.9%)  All AE: 13/42 (31%)   **AAD:** nr | **Hurduc** B 2009  Act Paed |
| *-*erad/ *-all AE - AAD* | *L. rhamnosus GG* vs placebo  all had triple therapy (amox and clarithr and Omeprazole) for 7 days | 83 children,  66 done (20% attrition)  asymptomatic  *Hp +* inpatients POLAND | 2x 10^9^  per day  **Capsule** | 7 days  F/up: 6 wks  APP | **Hp-:** 23/34 (69%) , ns **Any AE:**  18/35 (51%)ns  p=0.8 **AAD:** 2 (6%) ns p=0.8 | Hp-: 22/32 (68%)  Any AE: 13/32 (41%)  AAD: 6/30 (20%) | **Szajewska** H 2009  JPGN |

**Hp---page 8**

|  | **Probiotic** | **Pop** | **Dose** | **Dura-tion** | **Probiotic** | **Controls** | **Reference** |
| --- | --- | --- | --- | --- | --- | --- | --- |
| *+*erad/ -anyAE *+AAD* | *S. boulardii*  CNCM I-745  vs no txt control.  All got triple therapy OAC (7 days)   **Single blinded** (UBT outcome assessor) [excluded study arm with S. boulardii and muco-protective agent as no comparative control group (n=330) | 991 adults Hp+  symptomatic (ulcer, gastritis)  932 done  *Attrition:* control: n=33 (10%) Sb: n=18 (5.5%) Sb+MPA: n=8 (2%)  SOUTH KOREA | 2.2 x 10^10^  **Capsule** | 4 wks  F/up:  4 weeks ITT | **SB only:** (n=330):  **Hp-**: 264/330 (80.0%) * p=0.01 **any AE**: 48 (14.5%) p=0.12  **AAD** 9/330 (3.3%) p=0.04 *X^2^*=4 | No txt control: (n=331) **Hp-:** 237/331 (71.6%) **Any AE**: 63 (19%)  **AAD:** (20/331) 6% | **Song** MJ  2010 Helicobacter |
| *- erad AE: nr +AAD* | *S boulardii* CNCM I-745 + 14 day triple therapy  (Amox+Clarthirtho+Lansoprazole)  vs Controls (triple therapy only) | N=223 Hp+   randomized adults  KOREA | dose nr  form nr | 2 weeks  F/up:  4 weeks by ^13^C-urea test  ITT | n=107  **erad**: 73/107 (**68.2**%) p=0.905 NS  **AAD**: 32/107 (29.9%)* P=0.041 | n=116  erad: 80/116 (69.0%)   AAD: 50/116 (43.1%) | **Lee** JY 2011 Asian Pacific Digestive Week, 1-4 October 2011, SUNTEC Singapore Mtg Ab. J Gastro & Hep 2011 26(S5):257 |

**Hp---page 9**

|  | **Probiotic** | **Pop** | **Dose** | **Dura-tion** | **Probiotic** | **Controls** | **Reference** |
| --- | --- | --- | --- | --- | --- | --- | --- |
| *-erad AE nr AAD nr* | **2 control groups***: S boulardii*  CNCM I-745 + triple (LanClarAmox) vs   **1.** **Triple** therapy (ACL for 14 days) vs  **2.** **Sequential** ther  (AmoEso x 5 days) then (EsoLevoMetro x 7 days) for a total of 12 days | 285 adults Hp+ ulcer or gastritis  273 done (4% attrition)  TURKEY | 5 x 10^9^ per day  only 250 mg/d    **Capsules** | 14 days  F/up: 5 wks  ITT | (n=98) **Hp-:** 71/98 (72.4%) **WORSE!**  No AE data  AAD nr | **Triple therapy controls:** Hp-: 82/95 (86.3%) p=0.02 **Sequential**: 85/92 (92.3%) p<0.05 **Triple Txt vs Seq**=ns | **Ozdil** K 2011  Hepata-Gastro-enterology |
| *-erad +AE AAD nr* | Probiotic milk  (*L. acidophilus* La5 + *Bifido. lactis* Bb12) [starter: Strept thermo] (n=30)  **vs 2 controls**: **C1:** heat-killed fruit milk (n=29) **C2:** acidified milk control (n=29)  All had triple: AmoClarOmer (7 days) during week 5 | 88 Hp+  adults [18-65 yrs old]  asymptomatic  0% attrition  GERMANY | 7.5 x 10^8^/d  **Fermented fruit milk   Double blinded** | for: 5 wks  F/up:  3 weeks  ITT | **Hp-**: 30/30 (100%) ns  **Any AE**: nr At week5: Change in GI/Hp symptom **scores**:  -1.4 +1.1 * vs milk | **heat-killed milk control: Hp-**: 29/29 (100%) Sym scores:  -1.2 +1.1 ns **Acidified milk control**:  **Hp-**: 29/29 (100%) Sym scores: +2.6 + 1.1 | **de Vrese** M   2011     J Dairy Research |
| *- erad + AE AAD: nr* | ***Concomitant*** *S. boulardii* CNCM I-745 and triple therapy x 7 days,  vs *controls*: triple therapy only x 7 days open (no placebo) TT: Amox + Clarith + rabeprazole for 7 days excluded group (n=45) with sequential S. boulardii | 135 Hp+ adults  >18 yrs old active gastritis 128 done (5% attrit) 9/2010-02/2012 CHINA | 500 mg/d  1.5 x 10^10^/d  **capsule** | 7 days  F/up: 4 wks  ITT | **Erad**: 34/45 (75.6%)  p=0.17 ns **AE:** 16/41 (39%) p=0.02 | controls: erad: 28/45 (62.2%)  AE: 28/44 (63.6%) | **Gao** C, Xie R Ma T, Wu S. 2012 Chin J Gastroentero. 17:555-7. **In Chinese** |

| **+/-** | **Probiotic** | **No Hp+.** | **Dose** | **Duration** | **Probiotic** | **Controls*** | **Reference** |
| --- | --- | --- | --- | --- | --- | --- | --- |
| *+*erad/ *+ AE -AAD* | *S. boulardii*  CNCM I-745  (Lab Biocodex)  All had triple therapy (Amox+clarithro +  omeprazole)  for 14 days  enrolled 9/09-01/2011 | 100 Hp+ adults with peptic ulcers  One hospital  0% attrition  CHINA | SB 500 mg/d   ~5 x 10^9^/d  vs no txt control  **Sachet** | 14 days    F/up:   1 yr   ITT | **Hp-:** 42/50 (84.4%)* p=0.04  Improved symptoms: 48 (96%)*  Recurred:  5 (10%)*  all AE : 8/50 (16%)* p<0.001 No SAE  AAD 3/50 (6%) NS | Hp-: 32/50 (64.4%)  Improved symptoms: 31 (62%)  Recurred:  13 (26%)  all AE: 34/50 (68%)  AAD: 8/50 (16%) p=0.2 | **Chu** Y  2012 African J Pharmacy & Pharmacology |
| *- erad AE - AAE -* | "PY" Probiotic yogurt. Strains not defined in paper  emailed author. He replied yogurt was: *Lactobacillus acidophilus* La5 + *Bifido. bifidum* Bb12.  vs non-probiotic yogurt control (blinded)  vs no yogurt control (open)  All had triple therapy (Amox, Clarithro, Pantoprazaole for 7 days) | 102 Hp + adults symptomatic  [18-85 yrs old]  88 done (14% attrition)  **Both double blinded controls and open (no txt) controls**  IRAN | nr cfu/day in paper  response in author email on dose:  ~2 x10^6^/d  300 mg/d  **yogurt** | for 7 days  F/up: 4 weeks  **APP analysis** | **Hp erad**: 19/31 (61%) NS  2.6% power  **Any AE**: 20/31 (64%) ns  **AAD:** 7 (22.6%) ns | **Non-probiotic yogurt:**  Hp-: 20/31 (64.5%) p=0.79 **Any AE**: 21 (68%) p=0.79 **AAD**: 8 (25.8%) p=0.77  **No yogurt control:** Hp-: 19/26 (73.1%) p=0.35 Any AE: 22 (85%)p=0.13 AAD: 8/26 (30.8%) p=0.48 | **Mirzaee** V 2012  Iranian Red Crescent Med J   data from author in email |

**H. pylori page 10**

**Hp continued- page 11**

|  | **Probiotic** | **Pop** | **Dose** | **Duration** | **Probiotic** | **Controls*** | **Reference** |
| --- | --- | --- | --- | --- | --- | --- | --- |
| *- erad AE + AAD +* | All got TT (AmoxClarithOmep) x 14 days. randomized to:  *S. boulardii*   CNCM I-745  vs open control | n=82  Hp+ children peptic ulcers (n=33) or chronic gastritis (n=49) CHINA | 250 mg/d | 2 wks  F/up 4 wks | **Erad: ITT** 37/41 (90.2%) ns **APP**: 37/38 (97.4%)* p<0.05 **AE:** 5/41 12.2%* less **AAD**: 5/41 (12.2%)* | Open cntrl: **Erad ITT** 33/41 (80.5%) **APP** 33/40 (82.5%) AE: 13/41 (31.7%) AAD: 13/41 (31.7%) | **Zhang** Y 2012 J Clin Pediat  *[In Chinese]* |
| *erad + AE nr AAD neg* | All got TT (AmoxClarithOmep) x 14 days. randomized to:  *S. boulardii*   CNCM I-745  vs open control | n=60  Hp+ children with chronic gastritis  CHINA | 500 mg/d | 2 wks  F/up 4 weeks | **Erad:** 27/30 (90%)* p<0.05  **AE**: sign less but data in Chinese **AAD**: 0/30 (0%) p=0.11 | Open cntrl: **Erad** 20/30 (66.7%)  AE: raw data? AAD: 4/30 (13.3%) | **Zhang** H  2013 Med J Chinese People Health *[In Chinese]* |
| *+*erad/ *+AAD AE nr* | *S. boulardii*  CNCM I-745 "UltraLevure"  vs no txt control  Both groups got triple therapy ( amoxicillin (2 g/d), clarithromycin (1g/d), & omeoprazole (40 mg/d) For 14 days.  Single blinded (Patients unaware of other txt arm) | Adults with dyspepsia. Of 125 screened, 70 enrolled (aged 18-75 yrs old at one Greek hospital) Randomized 36 to Sb and 34 to nothing. 60 done (14% attrition)  GREECE | 300 mg/d  6 x 10^6^/d  One **Capsule** was 50 mg with 10^6^ cfu/cap | 14 days  F/up: 6 wks   ITT | **Hp-:**  30/36 (83.4%)*, p=0.034  completed 33/36 (91.7%)   **AAD**: 1 (2.8%)*  p=0.026  **Any AE:** nr | **Hp-:**  control 20/34 (58.8%)  completed: 27/34 (79.4%) p=0.2  AAD:  7 (20.6%) | **Kyriakos** N  2013  Hosp Chronicles |

**H. pylori-page 12**

|  | **Probiotic** | **# Hp+.** | **Dose** | **Duration** | **Probiotic** | **Controls*** | **Reference** |
| --- | --- | --- | --- | --- | --- | --- | --- |
| *- erad +AAD AE nr* | "Yomogi" *S. boulardii* CNCM I-745 vs no txt control  All had triple: Amox/Clarith/Omeprazole (14 days) | 160 Hp+ adults gastritis or ulcers  IRAN | 1 x 10^10^ /day 500 mg/d **Capsules** | 2 weeks  F/up: 8 wks  ITT | **Hp-:** 70/80 (87.5%) p=0.35 ns **Any AE**: nr **AAD:** 10/80 (12.5%)* p=0.04 | Hp-: 65/80 (81.2%)  Any AE: nr AAD: 21/80 (26%) | **Zojaji** H 2013 Gastro & Hepatol |
| *erad: +  AE: nr  AAD nr* | All got triple ther: AmoxClarOmepra x 14 days randomized to: open control or “Lacidofil” *L. rhamnosus* R11 + *L. helveticus* R52 | n=45 Hp+  children  Ukrain xe | 6 x 10^9^  **capsules** | 3 wks  F/up: 1 wk | **Erad**: 24/25 (96%)* p<0.05  AE: not in abstract AAD: | Open cntrls: Erad: 14/20 (70%) AE: AAD: | **Abuturov** OE 2014 Contemp Pediatri [Ukrainaine] |
| *+ erad  AE - (trend)  AAD neg* | *L. acidophilus La-5 + Bifido. bifidum Bb-12* vs no txt control.  All had triple: [Amox or Metro] + Clarith + Omeprazole (14 days) | 100 Hp+ children peptic ulcers one site – a GI clinic 88 done (12% attrition)  Jan 2009-June 2010  CHINA | **<5 yrs old:**  1 x10^8^/d  **> 5 yrs old**: 2.1 x 10^8^/d  **Sachets** | 6 wks  F/up: none!  APP data: | **Hp- erad:**  APP: 36/43 (83.7%)* *X^2^*=4.3, p=0.04 ITT¨36/49 (73%) ns  **AE**: 5/43 (11.6%) p=0.07 Trend ITT-AE: 5/49 (10.2%) p=0.2  **AAD**: 3/43 (7%) p=0.7 ns ITT-AAD: 3/49 (6.1%) p=0.4 Less E coli | Hp-: APP: 29/45 (64.4%)  ITT: 29/51 (56.9%)  AE: 12/45 (26.7%) ITT AE: 12/51 (23.5%)  AAD: 5/45 (11.1%) ITT-AAD: 5/51 (9.8%)  No change in *E. coli* flora. | **Wang** YH 2014 WJ Microbiol Biotech  [data supplied by author email]  not funded |

**H pylori-- page 13**

|  | **Probiotic** | **# Hp+.** | **Dose** | **Duration** | **Probiotic** | **Controls*** | **Reference** |
| --- | --- | --- | --- | --- | --- | --- | --- |
| *-erad AE nr +AAD* | *S. boulardii*  CNCM I-745 *"*Bioflor"  vs no txt control  All had triple (Amox/Clarith/ Omeproz)  (14 days) | 240 children Hp+  (5-11 yrs old) with gastritis, ulcers or inflammation 0% attrition  CHINA | 500 mg/d  1 x 10^10^  **Capsules** | 14 days  F/up:  4 wks  ITT | **Hp**-: 102/120 (85%) ns  p=0.07  **AAD**: 27/120 (22.5%)* p=0.008   **Any AE:** nr | Hp-: 91/120 (75.8%)  AAD: 47/120 (39.1%)   AE: nr | **Zhao** HM 2014  Zhongguo Dang Dai Erke Za Zhi  [in Chinese] |
| *- erad  Any AE nr  AAD +* | *S. boulardii* CNCM I-745 vs. no treatment controls  All had triple erad therapy:  AmoxClarrithOmeprazole or Metro ClarOmp if allergic to Amox x 14 days | N=205 enrolled, Hp+ children (22 months-16 yrs) n=194 done (5.4% attrition) CHINA | 500 mg/d  **sachets** | 14 days  F/up: 4 weeks ^13^C urea breath test in subgroup of 42 kids>12 yrs old | **Erad**: 15/21 (71.4%) p=0.5 ns **Any AE**: nr  **AAD**: 12/102 (11.8%) p=0.004 Compliance to std ther 100% p=0.03 | Erad: 13/21 (61.9%)    AAD: 26/92 (28.3%) Complaince: 86/92 (93.4%) | **Bin** Zhang  2015  Ped Gastroentero, Hepatol & Nutrition |

## Inflammatory Bowel Disease (IBD)- treatment

**Page 1**

Inflammatory bowel disease may include ulcerative colitis, pouchitis or Crohn’s disease

Outcomes included: number of stools/day, or frequency of IBD relapse or time to relapse

|  | **Probiotic** | **No.**  **in study** | **Dose** | **Duration** | **Relapse (or noted) in Probiotic** | **Relapse(or noted) in Controls** | **Reference** |
| --- | --- | --- | --- | --- | --- | --- | --- |
| *+* | *Saccharomyces boulardii* CNCM I-745 vs placebo | 20 enrolled, 17 done | 1.5 x 10^10^ | 7 wks | 3.3*stools/  day at week 9 | 4.6 stools/day at week 9 | **Plein** K  1993 Z Gastro |
| *-* | *E. coli* Nissle 1917 “Mutaflor” + prednisone vs prednisone and placebo DB, R | 24 done one center in Germany | 5 x 10^11^ | 1 year | 4/12 (33%), ns | 7/12 (58%) relapse | **Malchow** HA 1997 J Clin Gast |
| *+* | *Saccharomyces boulardii* CNCM I-745 *+* mesalamine (1g/d)  vs mesalamine alone (3g/d)  R, open | 32 in remission in  Italy | 1 x 10^10^ | 6 months | 1/16 (6% ) relapse*  p=0.04  No AE | 6/16 (38%) relapse | **Guslandi** M 2000 Dig Dis & Sciences |
| *-* | *VSL#3 +* rifaximin (1.8 g/d) vs mesalazine (4 g/d) R, open | 40 post-op in Italy | 3 x 10^11^  (4g/d) | 9 months | 4/20 (20%)  ns p=0.3 | 8/20 (40%) | **Campieri** M 2000 abstract A4179 Gastroenterol |
| *-* | *VSL#3 +*  rifaximin | 40 in remission | 1.8 x 10^12^ | 9 mon | 4/20 (20%) ns relapsed | 8/20 (40%) | **Rizzello** F 2000  Dig Liv Dis |
| *_-_* | *Lactobacillus rhamnosus* GG vs placebo DB, R | 45  post-op in remission | 1.2 x 10^10^ | 1 yr | 3/23 (10.5%), ns | 2/22 (16.6%) | **Prantera** C 2002 Gut |
| *-* | *L. rhamnosus* GG + mesalazine (2.4 g/d) | 35 adults | 1.8 x 10^10^ | 1 yr | 2/12 (17%)  relapsed, ns | 3/12 (25%)  relapsed | **Zocco** MA 2003 (abstract) Gastro |

**IBD- page 2**

| +/- | **Probiotic** | **No.**  **in study** | **Dose** | **Duration** | **Relapse(or noted) in Probiotic** | **Relapse (or noted) in Controls** | **Reference** |
| --- | --- | --- | --- | --- | --- | --- | --- |
| *-* | *Lactobacillus rhamnosus* GG vs placebo | 11 with active disease | 2 x 10^9^ | 6 months | 2/4 (50%)  ns | 3/5 (60%) | **Schultz** M 2004 BMC Gastro |
| *-* | *L. rhamnosus* GG + inulin (295 mg) + std. therapy vs  placebo+std txt  DB, R+ inulin | 75 children (5-21 yrs) in remission in 11 US centers | 2 x 10^10^ | 2 yrs | 12/39 (31%) ns, p=0.18  time to relapse=9.8 months | 6/36 (17%)  time to relapse=11 months | **Bousvaros** A 2005 Infl Bowel Dis |
| - | *S. boulardii* CNCM I-745  vs placebo  All had steroids or salicylates. 32 centers (09/04-01/10)  FRANCE | 165 adults with Crohn's disease at remission for >4 wks, 159 done (4% attrition). | 2 x 10^10^/d  1 g/d  **Capsules** | 52 weeks with 3 month f/up | Remission: 38/80 (47.5%) ns  time to relapse: 40.7 wks  But sign better in non-smokers (34.6%, p=0.02). Smoking causes intestinal cell surface damage, more inflamm)  AE: 57% SAE: 11% | Remission: 42/79 (53.2%)    time to relapse: 38 wks, ns AE: 58% SAE: 9.9%  Relapse in non-smoking controls (72%) | **Bourreille** A 2013 Clin Gastro & Help |

| +/- | **Probiotic** | **No.**  **in study** | **Dose** | **Duration** | **Relapse in Probiotic** | **Relapse in Controls** | **Reference** |
| --- | --- | --- | --- | --- | --- | --- | --- |
| *_* | *E. coli* (Nissle 1917) vs  mesalamine (1.5 g/d) | 120 UC patients enrolled, 103 done | 2 x 10^10^ | 12 wks | 8/50 (16%) relapse  ns | 6/53 (11%) relapse | **Kruis** W 1997 AP&T |
| *_* | *E. coli* (Nissle 1917 vs 1-2.4 g/d low dose mesalamine  Both on gentamicin  DB, R | 116 UC patients | 1 x 10^11^ | 1 yr | 39/57 (68%) ITT relapse, ns  APP: 26/39 (67%), ns | 44/59 (73%) relapse ITT  APP: 32/44 (73%) | **Rembacken** BJ  1999 Lancet |
| + | VSL#3 (mix) | 40 with pouchitis | 1.8 x 10^12^ (6 g) | 9 months | **Recurred**: 3/20 (15%)* | **Recurred:** 20/20 (100%) placebo | **Gionchetti** P 2000 Gastroenterol |
| - | *L. rhamnosus GG* | 20 with pouchitis | 4 x 10^10^ | 3 mon | PDAI=8.0, ns** n=10 | PDAI=8.4 placebo n=10 | **Kuisma** J  2003 Alim Pharm & Ther |
| + | VSL#3 (mix) DB, R | 40 with pouchitis | 9 x 10^11^ | 1 yr | New episode: 2/20 (10%)* | New episode 8/20 (40%) placebo | **Gionchetti** P 2003 Gastroenterol |
| *+* | *L. rhamnosus* GG | 117 with surgery after UC | 1.4 x 10^10^ | 3 yrs | 3/39 (7.7%)* | 20/78 (26%) no txt*** historic controls | **Gosselink** MP 2004  Dis Col Rectum |
| + | VSL#3 (mix) DB, R | 36 with pouchitis | 3 x 10^12^  (6 g) | 1 yr | 3/20 (15%)* relapsed | 15/16 (94%) placebo | **Mimura** T 2004 Gut |
| *-* | *E. coli* (Nissle 1917) vs mesalamine (1.5 g/d)  DB, R | 327 UC enrolled, 222 done | 2.8 x 10^10^ | 1 yr | 73/162 (45%), ns (ITT) relapse  APP: 40/110 (36%) ns | 61/165 (37%)  APP: 38/112 (34%) | **Kruis** W 2004 Gut |
| *+* | *VSL#3 +* balsalazide vs balsalazide | 90 UC patients | 9 x 10^8^ | 8 wks | 86%* cured | 73% | **Tursi** A 2004 Med Mon Sci |

**IBD- page 3**

UC, ulcerative colitis; ; **PDAI mean symptom score

**IBD page 4**

|  | **Probiotic** | **No.**  **in study** | **Dose** | **Duration** | **Relapse or noted in Probiotic** | **Relapse or noted in Controls** | **Reference** |
| --- | --- | --- | --- | --- | --- | --- | --- |
| *-* | *L. rhamnosus GG* vs mesalazine (2.4 g/d) vs LGG + mesalazine | 187 UC patients | 1.8 x 10^10^ | 1 yr | LGG only: 10/65 (15%), ns versus LGG+mes: 10/62 (16%) | Open controls: 12/60 (20%) | **Zocco** MA 2006 Ali Phar & Th |
| + | VSL#3 vs placebo | 15 adults pouchitis patients in remission,  on antibiotics | 1.8 x 10^12^ (6g/d) | 12 months, followed: no | 0/10 (0%)* also more diversity in normal flora found More E coli | 5/5 (100%) | **Kuhbacher** T 2006  Gut |
| *+* | VSL#3 vs placebo. All got steroid and mesalamine txt. | 29 children (2-16 yrs old) with new UC | varied by weight (4.5 x 10^2^-1.8 x 10^3^/d) | 1 year | 13 (92.8%) cured P<0.001 | Placebo: 4 (36.4%) cured | **Miele** E  2009 Am J Gastro |
| *+* | VSL#3 vs placebo | adults with mild to moderate active UC | 7.2 x 10^12^/d | 12 weeks | 33/77 (42.9%) remission, p<0.001 | 11/70 (15.7%) | **Sood** A  2009 Clin Gastro & Hepat |
| *-* | *E coli* Nissle 1917 (EcN) vs placebo | 90 with moderate UC | 10^8^ via enemas (10, 20 or 40 ml) | 2 weeks  followed for 6 wks | Remission:  10/23 (43.5%) 40 ml dose  11/23 (47.8%) 20 ml dose 8/22 (36.4%) 10 ml dose NS p=0.44 | placebo  7/20 (35.0%)  ITT analysis was ns, but PP was (don't count) | **Matthes** H 2010 BMC Comple & Altern Med |
| *+* | VSL#3 vs placebo | 144 patients with relapsing UC | 3.6 x 10^12^/d | 8 weeks | Decrease in UCDAI of 50% or more: 63.1%* P-.01 | placebo:  40.8% had 50% decrease in UCDAI | **Tursi** A  2010  Am J Gastro |

UC, ulcerative colitis; UCDAI, ulcerative colitis daily activity index

## Irritable Bowel Syndrome (IBS)-treatment

|  | **Probiotic** | **No. randomized vs done** | **Dose** | **Duration** | **Probiotic** | **Controls** | **Reference** |
| --- | --- | --- | --- | --- | --- | --- | --- |
| *+* | *S. boulardii I-745* vs placebo | 34  ITT | 9 x 10^9^  **capsules** | 4 wks | decrease #stools  mean -2.2/d *  13/16 (81%) improved | mean decrease -0.5/d, no raw # given  13/18 (72%) | **Maupas** JL 1983 Med Chirug Dig |
| - | *Lactobacillus rhamnosus* GG (LGG)  vs placebo arm CROSSOVER | 24,  19 done  Rome I  APP | 1 x 10^10^  **tablets** | 8 wks | 9/24 (37%) less pain, ns  10/24 (42%) bloating, ns | 3/24 (13%) less pain  7/24 (29%) bloating on  cross-over | **O'Sullivan** MA  2000 Dig Liver Dis |
| *+* | *Lactobacillus plantarum* DSM9843 (299v)  in rose hip drink vs placebo | 60  Rome II  52 done, APP, 13% lost | 2 x 10^10^  per day  (400 ml/d)  **drink** | 4 wks, 12 mon f-up | 11/25 (44%)* less gas | 5/27 (18%) less gas | **Nobaek** S 2000 Am J Gastro |
| *+* | *Lactobacillus plantarum* 299v  “ProViva” drink  ~50% IBS-C and ~49% IBS-A | 40  Manning criteria  ITT, 0% lost | 2 x 10^10^  **drink** | 4 weeks,  12 mon f-up | 9/20 (45%)* IBS resolved  no pain (0/20)* | 3/20 (15%) placebo  no pain 11/20 (55%) | **Niedzielin** K 2001 Eur J Gast Hepatol |
| - | VSL#3  (8 strains)  vs placebo | 25 IBS-D, Rome II USA | 9 x 10^11^  **powder** packet | 8 weeks | ITT:bloating score  -13.7*  4/12 (34%) cured ns | placebo bloating score  -1.7  5/13 (38%) cured | **Kim** HJ 2003 Al Pharm & Therapy |

IBS outcome diagnosed by Rome II or III criteria. Outcomes may include: individual IBS symptoms, or change in IBS-SSS (symptom scores from baseline) or decrease in number of stools/day, or number reporting more or fewer IBS symptoms

**IBS page 2**

| **+/-** | **Probiotic** | **No.** | **Dose** | **Duration** | **Probiotic** | **Controls** | **Reference** |
| --- | --- | --- | --- | --- | --- | --- | --- |
| *+* | *Bifido. infantis 35624*  *vs placebo* | 53, 49 done  Rome II  APP | 1 x 10^10^  **drink** | 8 wks | VAS=9.4 * pain  VAS=11.7*  bloating | VAS=14.9 pain  VAS=17  bloating | **O’Mahony** L 2005 Gastroentero |
| *-* | *L. rhamnosus GG vs placebo*  *underpowered for diarrhea outcome* | 58 kids Rome II  APP  50 done | 2 x 10^10^  **capsules** | 6 wks | 40% less abs pain, ns  2/17 (12%) less diarrhea, ns  0/17 (0%) bloating* | 44% less pain  0/18 (0%) less diarrhea  6/25 (24%) bloating | **Bausserman** M 2005 J Pediatr  under-powered |
| *+* | *VSL#3*  *yogurt*  *vs placebo* | 48  Rome II  ITT USA | 8 x 10^9^  **yogurt** | 4 wks | VAS=29.7* gas  VAS=31.3 bloat, ns  VAS=23 pain, ns  11/24 (46%) no bloating , ns | VAS=39.5  VAS=38.5  VAS=27  8/24 (33%) no bloating | **Kim** HJ  2005 Ali Pharm & Ther |
| *-* | *L. plantarum 299v* rose hip drink vs placebo | 66, APP 58 done (12% lost) | 2 x 10^9^  drink | 6 wks | Symptom score= 279 ns Improved: 10/29 (35%) NS | placebo mean score 245 Improved: 11/29 (38%) | **Simren** M 2006 Gastroenterol  Mtg abstract |

| **+/-** | **Probiotic** | **No.** | **Dose** | **Duration** | **Probiotic** | **Controls** | **Reference** |
| --- | --- | --- | --- | --- | --- | --- | --- |
| *+* | *3 dose arms:  Bifido. infantis 35624  (low dose)* vs placebo | 362 women  APP  n=90 | **capsules**  1 x 10^6^ | 4 wks | Symptom score (SS):  SS=2.15, ns  Cured 40/90 (44%) ns | placebo (n=92)  SS=2.09  cured 38/90 (42%) | **Whorwell** PJ  2006 Amer J Gastro |
| *+* | *Bifido. infantis 35624 (medium dose)* | n=90 | 1 x 10^8^ |  | SS=1.76* (also gas/bloat)  **cured 56/90 (62%) ***  **less pain score 10^8^ only** |  | **Whorwell** PJ  2006 Amer J Gastro |
| *-* | *Bifido. infantis 35624 (high dose)* | n=90 | 1 x 10^10^ |  | SS=2.13, ns (high dose clumped)  cured 33/90 (37%) ns |  | **Whorwell** PJ  2006 Amer J Gastro |
| *+* | *L. rhamnosus* GG vs placebo  *Large study of n=104 children with FAPD (function abd disorders: dyspepsia or IBS or abd pain). This is only IBS sub-set* | n=37  kids with IBS  POLAND | 6 x 10^9^ **capsules** | 4 wks  F/up: none | ITT IBS symptoms no abd pain: 6/18 (33%)*  AE: none | IBS sub-set: placebo: No abd pain: 1/19 (5%) | **Gawronska** A 2007  Arch Iran Med |
| *-* | **“**Activia**”** *Bifido animalis DN173-010 [starters: Strept thermo + L. bulgaricus]* yogurt vs  dead yogurt | 274 IBS-C, 267 done (2% attrition) | 2.4 x 10^10^/day  **yogurt** | 6 wks F/up: none | Living  *Responders* (>10% improved): 63% ns *Bloating score*: -0.5 ns *HRQoL* score change: +12.2 + 16.2 | **DEAD** heat-treated yogurt: *Responders* 56.8% *Bloating*: -0.31 *HRQoL* score change: +13.5 + 19.3 ns | **Guyonnet** D 2007  Alimen Pharmacol Ther |

**IBS –page 3**

**IBS page 4**

|  | **Probiotic** | **No.** | **Dose** | **Duration** | **Probiotic** | **Controls** | **Reference** |
| --- | --- | --- | --- | --- | --- | --- | --- |
| *+* | “Activia”  *L. casei* DN 173-010  now called *Bifido. lactis*   [+ 2 starters: *Strept* *thermophilus & L. bulgaricus]* vs control milk | 41 females (20-69 yrs old) IBS-C. If 41 enrolled, 38 randomized (3 dropped early). n=34 completed, but ITT. 2 had missing data | (2 x 10^10^cfu /day  **milk** | 4 wks,  F/up: 0 | (n=17)   Significant reduction in bloating & abd distension Figures show sign reductions  Overall IBS score at 24 wks:  3.3 + 0.15, p=0.03*. | Fermented milk control: (n=17)        Overall IBS-SSS: 3.8 + 0.4 | **Agrawal** A 2009 A  Alim Pharacol Therap |
| *+* | *L. plantarum* 299v vs placebo | 214 with Rome III IBS (63 female, 151 men, adults (19-70 yrs old) in 3 centers in India | 1 x10^10^  cfu/d | 4 wks  F/up: nr | Global improved: 80.6%*  Less Abd pain score: 53%*  Less bloating score:  68%* | Global improved:  8.8%  Less abd pain score:15%  Less bloating score:  20%* | **Sawant** PD & Ducrotte P 2010  DDW Mtg abstract, Chicago |
| *+* | *VSL#3* vs placebo | 59 kids (4-18 yrs old) in 5 Ped care in Italy & India | 1 sachet/  day (kids 4-11 yrs) or 2 sachets (kids 12-18 yrs old)  no cfu/d data | 6 wks then 2 wk washout then 6 wks other txt | Global improvement of symptoms: 2.1 (P<0.05) | Global improvement: 2.9 | **Guandalini** S 2010 J Ped Gastroenterol Nutr |

**IBS continues page 5**

|  | **Probiotic** | **No.** | **Dose** | **Duration** | **Probiotic** | **Controls** | **Reference** |
| --- | --- | --- | --- | --- | --- | --- | --- |
| *+* | *L. rhamnosus* GG vs placebo | n=141 children IBS or functional pain in 9 sites and a referral center ITALY | 6 x 10^8^/d | 8 wks,  F/up: least 4 wks | Success at week 12: 48/71 67.6%) p=0.03  AE: nr | Success in placebo at 12 wks:  37/70 (52.9%) | **Francavilla** R 2010  Pediatr |
| *-* | *“Bioflor” S. boulardii  I-745* [Biocodex] vs placebo | 67 IBS-D or IBS-A patients in Seoul   **KOREA** | 4 x 10 ^11^ | 4 wks | (n=34) Overall IBS symptoms-NS((1.2 at 4 wks)  but sign improved QOL 15.4% p<0.05 Score =70.9 day 1 and 80.8 at 4 wk) No AE QOL: -9.9 + 0.5 | (n=33)  IBS score=1.3 at 4 wks)  QOL improved in 7% Score: 74.8 Day 0 and 79 at 4 wks. n=1 pain/gas QOL: +4.2 + 0.4 | **Choi** CH  2011 J Clin Gastro |
| *-* | *S. boulardii* I-745 vs controls  Bangladesh June 2004-July 2005 | n=70 adults with diarrhea predominant IBS | 500 mg/d  1 x 10^10^/d | 1 month  &  f/up: 1 month | (n=35) #BM/d:  2.9 + 1.4, p=0.6 Change in abd. pain score:  0.47 + 0.7, P=0.6 | (n=35) #BM/d:  2.7 + 1.3 Change in abd. pain score: 0.4 + 0.74 | **Kabir** MA 2011 Mymenshingh Med |
| - | VSL#3 Mix of: *3 Bifido (longum and infantis and breve) +4 Lacto. (acidop and casei and delbrueckii and plantarum) and Strept thermophilus.* vs placebo | n=24 adults IBS-D   USA | 9 x 10^11^/d **capsule** | 8 wks | (n=15) Global score change: -1.3+ 0.6 ns Better “satiety” No change in flora | placebo (n=9) Global score change:  -0.7+ 0.7  AEs: none, ns | **Michail** S 2011 Probiotics Antimicrob Proteins |

|  | **Probiotic** | **No.** | **Dose** | **Duration** | **Probiotic** | **Controls** | **Reference** |
| --- | --- | --- | --- | --- | --- | --- | --- |
| *+* | *L. plantarum* 299v  (aka DSM 9843) vs placebo | IBS adults n=214 n=204 done (5% attrit) FRANCE | 1 x 10^10^/d **capsules** | 4 weeks  F/up: 0 | (n=105) Less abd pain: 51.9%*  (p<0.05) BM/d sign lower 78.1%*improved | Placebo (n=106) Less abd pain: 13.6% 8.1% improved  no AEs, ns | **Ducrotte** P 2012 World J Gastro |
| *+* | *S. boulardii*  I-745 (Biocodex) vs placebo.   All given ispaghula husk (fiber) as std txt*.* | n=72 adults with IBS-D in Pakistan  Pilot to study serum cytokines. | 750 mg/d | 6 wks  F/up: 0 | (n=37) only 1/8 diarrheal symptoms improved (abd pain score -0.04 +0.9, p=0.005).  Sign increase in anti-inflam IL-10 +1.6 +1.7* Sign reduced pro-inflam IL-8  -3.3 +4.7* and TNF -4.0 +4.3* Better QOL (no data given) | (n=35) placebo abd pain score change since baseline= +0.3+0.5.  IL-10: +0.4 +1.4  IL-8: -0.6+2.2   TNF:-0.7 + 3.4 | **Abbas** Z 2014 Euro J Gastro & Hepatol *Confirms Choi 2011 Pilot and underpoweredstudy, strong placebo effect-all better over time-due to fiber?* |

**IBS continues—page 6**

## Pediatric acute diarrhea-treatment

Pediatric diarrhea defined as new onset of acute infectious gastroenteritis symptoms (< 7 days duration) due to viral or bacterial etiologies but may be idiopathic. Outcomes defined as ‘cured’: resolution of diarrhea symptoms (typically >3 loose/watery stools/day) in subjects aged 1-18 years old, or change in Bristol stool scores, or mean duration of diarrhea

| **+/-** | **Probiotic** | **Age of subjects (months)** | **No.in study** | **Dose per day** | **Duration of treatment (days)** | **Percent cured or duration of diarrhea (days)**  **Probiotic \| Controls** | | **Reference** |
| --- | --- | --- | --- | --- | --- | --- | --- | --- |
| *+* | *S. boulardii* CNCM I-745 *+* ORT vs ORT control. Not randomized | 0.5-30 | 38 | 500 mg  1 x 10^10^ **sachet** | 5 | 18/19 (95%*) cured dura nr | ORT only 15/19 (79%) | **Chapoy** P 1985 Ann de Ped |
| *+* | *S. boulardii* CNCM I-745 vs placebo  *(SAME AS Cetina-Sauri 1994!!!!* | 3-36 kids (3 mon-3 yrs) acute diarr MEXICO | 130 | 2 x 10^10^ [800 mg/d] | 4  F/up: none | 55/65 (85%*) cured mean=2 d no Std dev | 26/65 (40%) RR cure on Day 4 yes=1.9 (1.4-2.8) mean=3 d | **Cetina-Sauri** G 1989 Trib Med [in Spanish] |
| + | *L. rhamnosus GG* | 4-45 | 71 | 2 x 10^10^-10^11^ | 5 | 1.4 d* | 2.4 d | **Isolauri** E 1991 Pediatrics |
| + | *L. rhamnosus GG* | 7-37 | 39 | 2 x 10^10^-10^11^ | 5 | 1.1 d* | 2.5 d | **Kaila** M  1992 Pediatr Res |
| *+* | *L. rhamnosus GG* | 5-28 | n=42 | 2 x 10^10^ | 5 | 1.5 d* | 2.3 | **Isolauri** E 1994 Dig Dis Sci |
| *+* | *Lactobacillus acidophilus* LB (killed strain) vs placebo vs active control (loperamide) All had either ORT or IV rehydration | 1-54 months, inpatients kids with acute diarrhea FRANCE | 103 in, 103 done (0% attrition) | varied  3 x 10^10^ Day 1, then 2 x 10^10^ for 3 days **sachets** | Mean 4 days  F/up: none | **Dead** Time to recovery: 49.7 + 30 hours (2 days)* n=38 no AE | **Placebo** 64.7 + 30 hours (2.7 days), n=33 **Loperamide** 60.9 hrs (n=32) | **Boulloche** J 1994 Ann Pediatr (in French) |
| - | *L. rhamnosus GG (alive) vs L. rhamnosus GG (dead)* | <4 yrs old txt rotaviral diarr, inpatient | 41, 26 rotov+ | 2 x 10^10-11^ **liquid** | 5 days F/up: none | Live LGG: 10/12 (83% had rotoviral IgA)* duration diarr=1.5 d NS | Dead LGG: 2/13 (15%) roto IgA+ duration diarr= 1.6 days | **Kaila** M  1995 Arch Dis in Childhood |

|  | **Probiotic** | **Age of subjects (months)** | **No.**  **in study** | **Dose given per day** | **Duration of treatment (days)** | **Probiotic** | **Controls** | **Reference** |
| --- | --- | --- | --- | --- | --- | --- | --- | --- |
| *+* | *Saccharomyces boulardii*  CNCM I-745 vs placebo | 11-35 | 18 | varied | 30 | 4/7 (57%) cured* | 2/8 (25%) dura: nr | **Chouraqui** JP 1995 J Ped Gastro Nutri |
| *+* | *S. boulardii*  CNCM I-745 vs placebo (Biocodex) Cuba | 6-36 mon chronic diarr 90% Giardiasis | 40 | 1 x 10^10^ **sachets** | 30 | 14/20  (70%) *  improved No AEs | 2/20 (10%) placebo | **Guillot** C 1995 Rev Mex de Pueric Y Ped |
| *+* | “Lacidofil” *L. acid [helveticus* R52]+ *L. rham* R11) vs control (clay, smectite) | kids with acute diarrhea **Czech** | 75 | 2 x 10^9^/d | varied by duration of diarrhea | (n=33) 4.8 + 2.1 days, p<0.05 | (n=42) 8.7 + 4.2 days | **Tlaskal** P 1995  Cesko-Solven Ped  [In Czech] |
| + | *L rhamnosus* GG vs *L. casei ST vs L. casei* LD- all active controls | 6-35  txt acute rotavir diarrhea | 49 | 1 x 10^10^ 5.5 x 10^8^ 6 x 10^9^ | 5 vs 5 vs 5 | 1.8 d*  Lr GG | vs. 2.8 d L. casei no placebo controls | **Majamaa** H 1995 J Ped Gastroenterol Nutri |
| + | *L. rhamnosus GG* | 1-24 | 32 | 2 x 10^10^-10^11^ | 2 | 69%* | vs. 25% | **Raza** S 1995 Ped Infect Dis J |
| + | *L. rhamnosus GG* | <24 | 39 | not given | 2 | 1.9 d*  but reported subgroup only | vs. 3.3 | **Pant** AR  1996 J Trop Ped |
| + | *L. rhamnosus GG* | 3-36 | 100 | 3 x 10^9^ | 5 | 3.3 d* | vs 5.9 | **Guarino** A 1997 J Pedia Gastroent Nutr |

**Ped diarrhea-page 2**

|  | **Probiotic** | **Age of subjects (months)** | **No.**  **in study** | **Dose given per day** | **Duration of treatment (days)** | **Probiotic Percent cured or duration of diarrhea (days)** | | **Controls** | **Reference** |
| --- | --- | --- | --- | --- | --- | --- | --- | --- | --- |
| *+* | *S. boulardii*  CNCM I-745 vs placebo  All got ORT DBPC Jan 1-Aug 30, 1996 | Hospitaized kids (mean 21 mon Sb or 22 mons old-plac) with mild dehyration & diarrhea, 1 site Mexico | 50 | 600 mg/d | 5 days  F/up: none | Sb: Cured by Day 4: 24/25 (96%)* p=0.01 no data on duration  No AE | placebo cured 18/25 (28%) | | **Hernandez** CL 1998 Revist Enferm Infecc Ped [in Spanish] |
| *+* | *L. casei* DN114001 [CNCM I-1518]  Actimel yogurt  txt of acute diarr | 7-32  Kids in 12 day cares | 287 | Aged 6-18 mon (3 x 10^10),^ over =6 x 10^10^) | 30 day  60 days follow-up | 4.3 + 2.7d* | Jellied milk: 8.0 + 5 days  Std yogurt: 5.3 + 2.5 | | **Pedone** CA 1999 Ind J C P |
| + | *L. rhamnosus GG* + ORT vs placebo + ORT, multi-site ITALY | 1-36 months old with acute diarrh | 287 | 4 x 10^10^ | varied | 2.4+ 1.1 d*   LOS sign. shorter | vs. 3.0 + 1.5 days | | **Guandalini** S 2000 J Pediatr Gastro Nuti |

**Txt Ped Acute Diarr---page 3**

Abbreviations: d, day; LOS, Length of stay; ORT=oral rehydration therapy

**Txt Ped Acute Diarr---page 4**

| **+/-** | **Probiotic** | **Age of subjects (months)** | **No.in study** | **Dose per day** | **Duration (days)** | **Percent cured or duration of diarrhea (days)**  **Probiotic \| Controls** | | **Reference** |
| --- | --- | --- | --- | --- | --- | --- | --- | --- |
| *+* | *Lactobacillus  acidophilus* LB (heat-killed strain) vs placebo  All had ORT  Thailand | 3-24 months, inpatients  kids with acute diarr | 73 (0% attrition)  ITT | 2 x 10^10^ (5 doses over 48 hours) | 2.5 days  F/up: 2 days   AE: nr | Dead: 1.8 d p=.03 (43.4 + 25.9 hrs) Cured: 7/37 (19%) p=0.03 | Placebo: duration:2.4 days (57 + 36.3 hrs) Cured Day 2: 1/36 (2.8%) | **Simakachorn** N 2000 J Ped Gastroent Nutri |
| *+* | *L. casei DN114001* [aka CNCM  I-1518] [starters: L. bulgaricus + Strept thermophiles] (Actimel) vs Indian Dahi yogurt vs heat-treated yogurt | 6 mon-5 yrs with diarrhea either inpatients or community | Pilot. n=110 | 3 x 10^10^/d  **yogurt** | not given in paper | duration= 1.5d inpatients P<0.05, but not community 2.0d. | duration inpatient yogurt (2d) vs heated yogurt (2 d) | **Agarwal** KN 2001  Indian Ped |
| + | *S. boulardii* CNCM I-745 vs placebo | 2-29 month  TURKEY | 100 | 5 x 10^9^ | 3 d  f/up: none | 42/50 (84%)* cured no duration data | 32/50 (64%) | **Urganci** N 2001 Arch Gastro |
| *+* | *S boulardii* CNCM I-745 *+ ORS* vs ORS open control | 6 mon-5 yrs    PAKISTAN | 101 | 500 mg/d | 6 days | On Day 6, % with diarrhea: 9/51 (17%)*  Also -1.1d diarrhea: x=3.6 +1.6 days (n=51) | ORS given only % diarrhea: 18/50 (37%) x=4.5 + 1.6 (n=50) | **Hafeez** A 2002  J Coll Phy Sur Pak |
| + | *L. casei DN114001 [CNCM I-1518] & starters: [L. bulgaricus + Strept thermophiles] (*Actimel) vs Indian Dahi yogurt vs heat-treated yogurt | 6 mon-5 yrs in India | 150 (75 hosp and 75 comm) | 300 mg/d~ 3 x 10^10^/d | not stated in paper | hosp kids duration 1.5 + 0.5 d*  comm.-unity:  1.9 + 0.8 days* no normal flora done | heat-killed yogurt:  hosp 2.1 + 0.7 comm  2.4 + 0.9 | **Agarwal** KN 2002  Eur J Clin Nutr |

| *+/-* | **Probiotic** | **Age of subjects (months)** | **No.in study** | **Dose per day** | **Duration (days)** | **Percent cured or duration of diarrhea (days)**  **Probiotic \| Controls** | | **Reference** | |
| --- | --- | --- | --- | --- | --- | --- | --- | --- | --- |
| - | *L. rhamnosus GG* | <24 | 124 | 1 x 10^6^ | 7 | 1.6 d | vs. 1.6 ns | **Costa-Ribeiro** H 2003 J Ped Gastroenterol Nutr |  |
| - | *S. boulardii* CNCM I-745 *(n=30)* vs controls (n=29) also see below Argentina | 6-24 mon persistent diarrhea (>14 days) | 59 | 2 x 10^12^  per day **powder in milk** | 5 days | #stools/d:  2 + 2  Duration diarr: 25+3 d NS n=30 | n=29 Milk control 5+3 stools/day 22+2 days duration | **Gaon** D 2003 Medicina |  |
| + | *Saccharomyces boulardii* CNCM I-745 vs placebo.   All had ORT  Turkey | 3-84 mon   41% rotavirus or Shigella | 200 kids acute diarr | 250 mg 5 x 10^9^  powder  diluted in liquid | 5 days  and 14 days f-up | (n=100) **duration**: all diarrhea 4.7 +2.5d*, p=0.03 watery diarr 2.8 + 1.0 d  **LOS**:  2.9 + 1.2* | (n=100) duration: all diarrhea  5.5 + 3.2  watery diarr 3.8 + 1.4 d **LOS**:  3.9 + 1.5 d no serious AEs | **Kurugol** Z 2005 Acta Paediatri | |
| + | 3 arms: *1. Lacidofil (L. acido[helveticus R52] + L. rhamn R11)* vs 2. placebo vs  3. 'Hylac"*-prebiotic* | kids acute gastritis (12-72 mon old) **CZECH** | 113 | 2 x 10^9^/d  **capsules** | 10 days F/up: none | 1. probiotic: (n=42) duration= 4.0 +2.0 d* p<0.05 2. “Hylac" (n=29) ns 6.1 + 3.2 d | (n=39) placebo: duration= 5.4 + 2.3 d | **Tlaskal** P  2005  Nut Aliments Fonction Alimen Sante | |

**Txt Ped Acute Diarr---page 5**

**Txt Ped Acute Diarr---page 6**

|  | **Probiotic** | **Age** | **No. in study** | **Dose given per day** | **Duration** | **Probiotic [** | **Control** | **Reference** |
| --- | --- | --- | --- | --- | --- | --- | --- | --- |
| **+** | *S. boulardii* CNCM I-745 *+* ORT vs controls (ORT only)   Pakistan | 2-12 yrs old mild-moderate acute diar | 100 | 500 mg  1 x 10^10^  **powder**  (diluted in water or food) | 5 days,  followed 2 mon | (n=50) 3.6 + 1.0 days diarr* p=0.001 no AE | (n=50) 4.8 + 1.4 d (ORT only control) | **Billoo** AG  2006  World J Gastro |
| **+** | *S boulardii* CNCM I-745 + ORT  vs placebo + ORT  Argentina  All also got ORT | 3-24 mon old,  outpats  with mild-moderate diarrhea | 100  88 done txt, 72 done f/up  not ITT | 5 x 10^9^ if < 1yr old  1 x 10^10^ if > 1 yr old  **capsules** | 6 d  F/up: 1 month | (n=44 ITT) or [n=35 APP] duration: 4.7 +1.9d*  (2-13 days range) | (n=44 ITT) or [n=37 APP]  mean duration: 6.2 + 3.2 d  (range 2-10 d) | **Villarruel** G 2007**A** Acta Ped  [also in Villarruel 2007**B** meeting abstract in J Ped Gastro & Nutr] |
| + | *L. acidophilus LB (heat killed)* + ORT "Lacteol Forte" vs placebo  All had ORT ECUADOR | Txt ped diarrhea in kids: mean age=10 months eligible if <2 yrs, inpatients | 80  0 lost,  0% attrition | 2 x 10^10^ per day   **sachets** | 3 days  F/up: 1 day  ITT | Dead cured 36/42 (86%)*  duration diarrhea: 39.5 + 10.5 hrs, p<0.05 | placebo + ORT cured 20/38 (53%)  duration= 63.4 + 30 hrs | **Lievin-Le Moal** V  2007 Pediatrics |

|  | **Probiotic** | **Age of subjects (months)** | **No.**  **in study** | **Dose**  **given per**  **day** | **Duration of treatment**  **(days)** | **Percent cured or duration of diarrhea (days) in**  **probiotic \| ORT + ORT \| controls** | | **Reference** |
| --- | --- | --- | --- | --- | --- | --- | --- | --- |
| + | *L. rhamnosus GG* + ORT “Dicoflor” vs ORT control | 3-36 | 192 | 6 x 10^9^ **in water** | 5 | 3.3 + 1.5 d *  n=100 | (n=92)    4.8 + 1.0 d    Median= 115.5 hrs | **Canani** RB 2007 BMJ |
| - | *S. boulardii I-745* + ORT “Codex” vs ORT control | 3-36 | 183 | 5 x 10^9^  **in water** | 5 | 4.4 + 0.93 days ns Md=105 hrs n=91 |  | **Canani** RB 2007 BMJ |
| - | 4 strains of *Bacillus clausii* (O/C84, N/R84, T84,SIN84)+ ORT “Enterogermnia” vs ORT control | 3-36 | 192 | 1 x 10^9^  **in water** | 5 | 4.9 + 1.03d ns Md=118 hrs n=100 |  | **Canani** RB 2007 BMJ |
| - | *E. faecium SF68* + ORT “Bioflorin” vs ORT control | 3-36 | 183 | 7.5 x 10^7^  **in water** | 5 | 4.7 + 1.7 d (n=91) ns |  | **Canani** RB 2007 BMJ |
| *FROM CANINI 2007: Strains Daily dose Brand name  Lactobacillus casei rhamnosus GG 6×10^9^ CFU/dose Dicoflor  Saccharomyces boulardii S boulardii It 5×10^9^ Codex  Bacillus clausii O/C84, N/R84, T84, SIN8 4x 10^9^ CFU/dose Enterogermina  Enterococcus faecium SF 68 7.5×10^7^ CFU/dose Bioflorin†  10^9^ CFU, 10^9^ CFU, 10^9^ CFU, 5×10^8^ CFU/dose Lactogermina | | | | | | | | |

**Txt Ped Acute Diarr---page 7**

**Txt Ped Acute Diarr---page 8**

| **+/-** | **Probiotic** | **Age of subjects (months)** | **No.**  **in study** | **Dose**  **given per**  **day** | **Duration of treatment**  **(days)** | **Percent cured or duration of diarrhea (days) in**  **probiotic \| controls** | | **Reference** |
| --- | --- | --- | --- | --- | --- | --- | --- | --- |
| *+* | *S. boulardii* CNCM I-745 with ORT  vs control placebo | Children 6 mon-10 yrs with acute diarrhea Oct 2004-March 2005 TURKEY | 27 | 0.5 g /d | 7 days  F/up: none no duration diarrhea data | (n=16) By day 4, sign less stools/d: 0.4 + 0.2* also more IgA | (n=11) ORT only, day 4: 1.8 + 0.4, p<0.001 No AEs | **Ozkan** TB 2007  J Int Med Res |
| *-* | *"*Lacteol*"  L. acidophilus LB (heat-killed)* + ORS vs   placebo + ORS | Txt of kids with acute ped diarrhea (3 months-4 years old) outpatients  PERU | n=80  3 lost,  77 done, (4% attrition), but ITT done | 3 x 10^10^ Day 2, then  2 x 10^10^ max 4.5 days  **sachet** | 4.5 days  F/up: none | **DEAD**: 36/40 (90%) cured, p=0.33 NS Median duration diarrhea= 10 hrs, p=0.3 NS AE: 30% | Placebo:cured: 35/40 (87%)  Median duration= 16.6 hrs  AE: 15% | **Salazar-Lindo** E  2007 J Ped Gastroenterol & Nutri |
| + | *L rhamnosus GG* vs ORS only control | kids with persistent diarrhea 1 hosp, inpatients  INDIA | 253 random. (7.1% attrition), 235 done | 1.2 x 10^8^/d | minimum of 7 days or until no more diarrhea. No f/up | Mean duration all diarrhea: 5.3 + 2.1 d, p<0.05 Duration CDI: 3.2 + 2.4 d p<0.05 | Mean duration all diarrhea: 9.2 + 2.8 d Duration CDI: 8.0 + 2.8 d p<0.05 | **Basu** S  2007 J Clin Gastroenterol |
| + | *S. boulardii* CNCM I-745 vs placebo  All had ORT | India and Indonesia kids (3-33 months old) acute diarrhea | 202 kids, 188 done (7% attrition) | ORT+ 500 mg/d **formulation not reported** | 5 days  F/up: none | (n=93) Duration: 2.2 + 1.6 d* Cured day 5: 97% p=0.13 ns | (n=95) Duration: 2.8 + 2.2 d Cured day 5: 90% | **Vandenplas** Y 2007  J Ped Gastro Nut [Euro Soc Ped Gastro Hep & Nut Mtg abstract] |

|  | **Probiotic** | **Age of subjects (months)** | **No. in study** | **Dose per day** | **Duration (days)** | **Percent cured or duration of diarrhea (days) in**  **probiotic \| controls** | | **Reference** |
| --- | --- | --- | --- | --- | --- | --- | --- | --- |
| **+** | *S. boulardii*  CNCM I-745 *+ ORT* vs placebo + ORT  Myanmar | 3 mon-10 yrs 21% E. coli acute diarrhea. May not be randomized “groups assigned alternatively” | 100 (n=50 each group) | 500mg/d  **formulation not reported** | 5 days | duration 3.08 + 0.95 d* n=50 | 4.7 + 1.2 d n=50 | **Htwe** K  2008  Am J Prop Med Hyg |
| *+* | *S. boulardii*  CNCM I-745 *+* metro  vs control (metro only)   open RCT | mean 11.7 + 2.1 yrs old acute bloody diarrhea by *Entamoeba histolytica*  TURKEY | n=50 | 500 mg/d 1 x 10^10^  metro (60 mg/kg/d)  **capsules** | 7 days,  1 month follow-up | (n=25) duration diarr: 46.1 + 18.2 hr * 1.8 + 0.7d*  all cysts cleared by day 5 | (n=25) 73.9 + 32.4 hrs or 3.0 + 1.2 d  On day 5: 6/25 still cyst positive | **Dinleyici** EC 2009  Am J Trop Med |
| *+* | *VSL#3* vs placebo Italy | 1-16 yrs old, kids with ulcerative colitis, all given steroids and mesalamine | n=29 | 4.5-18 x 10^11^/d | 1 year | cured 13/14 (92.8%)p<0.05* no AE | placebo 4/15 (36.4%) cured | **Miele** E  2009 Am J Gast |
| *-* | *S. boulardii*  CNCM I-745 (Reflor) + metro vs metro only control Open trial  Turkey | 1-15 yrs old with *E. histolytica* diarr | n=90 n=5 lost in Sb (non-compliant) 6% attrition | 250 mg/d (5 x 10^6^/d)  Metro: 30-50 mg/kg/d | 10 days  F/up: 10 d  no std dev data | (n=40) duration diarr: median 4.5 days , p=0.96 no mean sd data | (n=45) metro only median= 5 days No AEs | **Savas-Erdeve** S & Gokay S  2009  Turk J Ped |

**Txt Ped Acute Diarr---page 9**

**Txt Ped Acute Diarr---page 10**

|  | **Probiotic** | **Age of subjects (months)** | **No. in study** | **Dose per day** | **Duration (days)** | **Percent cured or duration of diarrhea (days) in  probiotic** | **Controls** | **Reference** |
| --- | --- | --- | --- | --- | --- | --- | --- | --- |
| *+* | *S. boulardii*  ( CNCM I-745 Reflor®, Sanofi-Aventis, Turkey)   vs fluid-extract from Pinar (*L. bulgaricus and S. thermophilus)* 10^7^/100 ml yogurt   not blinded, open trial ORT & Zinc as needed | children with acute non-bloody diarrhea;x=  21 + 28  mon old  [5-168 mon] 61-81% de hydrated  TURKEY | 67 enrolled, 55 done  (18% attrition) | SB: 500 mg (kids >2 yrs) or 250 mg if <2 yrs.  in **fluid**  30-60 ml/d dependent on age | 5 days | APP:  Diarrhea resolved by Day 3: SB 13/28 (46.4%)   ITT was Sb 48.5%*  ITT Duration diarrhea: 4.45 + 2.5d ns effect not sustained by Day 5 | Cured  yogurt (6/27, 22.2%) p=0.06  ITT 25.5%, p=0.03  denominator not given  ITT duration: 5.4 + 3.1 day NS | **Eren** M  2010  Am J Trop Med Hyg |
| *+* | *S. boulardii + ORT* vs   placebo + ORT | n=76 Children  (1-23 mon) with rotaviral diarrhea  n=64 done (16% attrition)  BOLIVIA | n=41 | 8 x 10^10^/d  **powder in water** | 5 days  no follow-up | (n=21) ORT+ SB: median duration diarr= 58 (IQR 41) or 2.42 + 1.27d (p=0.04) | (n=20) ORT + placebo:  median (IQR) duration diarr= 84.5 hrs (94 IQR)  or est. 3.5 +2.9**d** | **Grandy** G 2010 BMC Infect Dis  std dev from   Dinleyici EC 2012] |

|  | **Probiotic** | **Age of subjects (months)** | **No. in study** | **Dose per day** | **Duration (days)** | **Percent cured or duration of diarrhea (days) in  probiotic** | **Controls** | **Reference** |
| --- | --- | --- | --- | --- | --- | --- | --- | --- |
| *+* | *VSL#3* vs placebo | 4-18 yrs old with IBD, cross-over trial with 2 wk wash-out | 59 | 1 sachet (4-11 yrs old)  or  2 sachets (12-18 yrs old) | 6 weeks | 44/59 (74.6%) respond on VSL3. Mean Global symptom score= 2.2 (p<0.01) | 17 did not respond while on placebo Mean Global symptom score= 3.0 | **Guandalini** S 2010  J Ped Gastroent Nutr |
| *+* | *S. boulardii* CNCM I-745 in specific formula vs std formula controls (not the same formula as Sb patients) France | infants (1-9 mon old)(5.4 + 0.4 mon old) with acute diarrhea 4-5% de hydrated | n=70 | 200 mg/d **formula** | 6 days  F/up: none | (n=34) duration diarrhea: 35.4 + 3.7 hrs or 1.5 + 0.1 d, p<0.001 | (n=36) duration: 67.1 + 5 hrs or 2.8 + 0.2 days | **Le Luyer** B 2010 Archives de Pediatrie (in French) |
| *+* | *S. boulardii*  CNCM I-745 (Reflor) vs metro  vs control (no txt) Turkey | kids with *Blastocystis hominis* (leading GI pathogen in Turkey) | 48 | SB (500 mg/d)  OR  metro (60 mg/kg) | 10 days F:up: 5 days | cured **SB**: 14/18 (77.7%)* vs **metro** 10/15 (66.6%)ns | control: 6/15 (40%) | **Dinleyici** EC 2011 Parasito Res |
| *+* | *S. boulardii +*  CNCM I-745 Zinc vs Zinc only vs 6 other control groups (+ formula, + Zinc) vs ORS only control  Turkey | 1-28 mon peds with rotavirus diarrhea | 480 | 250 mg/d  **not reported formulation** | minimum of 5 days | (n=60) SB+Zn only: less diarrhea duration= 3.1 + 1.8 days, P<0.05 if SB only (n=60)  (4.8 + 1.5 days, NS) | (n=60) ORT control  duration: 5.3 + 1.8 days | **Dalgic** N 2011 Ped Int |
| *+* | *S boulardii*  CNCM I-745 (Floratil-Merck) vs placebo, Brazil | 6-48 mon 57% rotaviral, acute | 176 | 8 x 10^9^/d | 5 days  ITT no duration data | On day 3, 66/95 (69.5%) cured* | placebo 40/91 (44%) cured | **Correa** NB 2011  J Ped Gastroent Nutri |

**Txt Ped Acute Diarr---page 11**

|  | **Probiotic** | **pop** | **N** | **dose** | **duration** | **probiotic** | **controls** | **reference** |
| --- | --- | --- | --- | --- | --- | --- | --- | --- |
| + | *Bacillus clausii* + ORT versus open control ORT only  Phase 3 RCT  *See full publications Lahiri 2015A and 2015B of other site #2* | kids 6 mon-5 yrs old with acute diarrhea <48 hrs 1 of 6 sites  Mumbai, India | n=56   site #1: Seth GS Med College KEM Hospital  (from author) | 4 x 10^9^  per d  **spores** | 5 days  F/up: 5 days | (n=28) duration diarrhea= 44.8 hrs * p<0.01 no std dev data Md=41.3 Cured Day 3 40.7%* | (n=28) duration in ORT only: 74.5 hrs no std dev Md=67.5 hrs Cured Day 3 9.1% | **Lahiri** KR 2011 Abstract from 48^th^ Annual National Conference of Indian Acad of Ped Jan 20-23 2011 Jaipur INDIA **see other sites full papers Lahiri KR 2015 A-C** |
| *+* | *S. boulardii* + CNCM I-745 ORT vs placebo + ORT in puffed rice powder | Children  3-59 mon with acute diarrhea, hospitalized, 58% dehydrated India | 108 | 500 mg  **sachet** | 5 days | (n=54) duration= 2.2 + 1.0 days p<0.05 or [52.1 +24.6 hrs] | (n=54) duration 2.7 + 1.3 days   r [64.0 + 30.4 hrs] | **Riaz** M 2012 Indian J Pediatr |
| *-* | *S. boulardii*  CNCM I-745 Biocodex  ORT versus controls (ORT) | 5 mon-5 yrs old, all rotaviral diarrhea Turkey | n=50: Sb (n=25)and n= 25 control | 6 x 10^9^/d 282.5 mg/d “Reflor” **sachet** note, low dose!! | varied  f/up: none | duration SB= 6.6 + 1.7 days, ns p=0.4 n=25 | 7.0 + 1.6 days  n=25 | **Erdogan** O 2012  J Trop Med |

**Txt ed Acute Diarr---page 12**

|  | **Probiotic** | **pop** | **N** | **dose** | **duration** | **probiotic** | **controls** | **reference** |
| --- | --- | --- | --- | --- | --- | --- | --- | --- |
| *+* | *L. reuteri* DSM 17938 vs placebo  *Italy*  all given ORT | 74 children (6-38 months), inpatients 3 hosp in Italy with acute diar. | Of 74 enrolled, 69 done (7% attrition) | 4 x 10^8^/d | given 7 days  no AE | **duration**= 2.1 + 1.7 days, p<0.03 **Cured** day 3 (52%), p<0.02 | duration 3.3 +2.1.  Cured by day 3: 24% | **Francavilla** R 2012 APT vol 36 |
| *+* | *S. boulardii* CNCM I-745 *+*ORT vs std ORT (open study) | inpatient kids (2 mon-5 yr) with watery diarrhea 0% attrit **Pakistan** | N=420 | 500 mg/d **sachet** | 5 days  note: sd nr in paper, calculated | (n=210) Duration: 3.43 + 1.69 days, p<0.05  WMD duration: less by -25.7 hrs, p<0.05 | (n=210) Duration: 4.5 + 1.69 days .  RR day 3= 0.09 (0.05-0.16) | **Khan** A 2012 Pakistan Paed J |
| *+* | *S. boulardii*  CNCM I-745 *+ ORT vs control (ORT)  All had Zinc* | outpatient kids **India** | N=72 n=70 done (3% attrition) 07/09-07/11 | 500 mg/d **sachet** | 5 days | (n=35) duration: 3.4 + 1.4 days p<0.01 | (n=35) duration5.5 + 2.1 days | **Burande** MA 2013 J Pharm & Pharm |
| *+* | *S. boulardii*  CNCM I-745 *+* ORT vs ORT only control (open, randomized) *as part of a larger AAD trial* | Inpatient children (6 mon-14 yrs old) with acute AAD  **CHINA** | n=42 on placebo who developed diarrhea | 500 mg/d  **powder** | 5 days  F/up: none | (n=23) 91% **cured** p<0.05 & **duration** 2.3 + 0.95 d, P<0.01, No AE | (n=19) 21% cured  duration 8.97 + 1.07 days | **Shan** LS 2013 Beneficial Microbes |
| *-* | *L. rhamnosus GG* (ATCC 53103) vs placebo | Children (6 mon-5 yrs) with either rotavirus (n=82) or Crypto. (n=42) diarrhea **INDIA** | N=124 enrol, n=123 done (0.8% attrition) | 1 x 10^10^ /d  **capsules** | 4 weeks  F/up: none  *72% were 100% compliant,  all 123 done had >80%* | Duration diarr Med= 4 (IQR 3-6) NS  Repeated diarrhea episode: 25%* More IgG to rotavirus | Duration: Md=4 d (IQR 3-6)  Repeat episode: 46% | **Sindhu** KN 2014 Clin  Infect Dis |

**Treatment ped diarrhea-page 13**

|  | **Probiotic** | **pop** | **N** | **dose** | **duration** | **probiotic** | **controls** | **reference** |
| --- | --- | --- | --- | --- | --- | --- | --- | --- |
| *+* | *L. reuteri* DSM 17938  vs. open control group (Just ORS). All had ORS *single blinded* | Inpatient children with diarr (3-60 mon old) **Turkey** | n=140 n=127 done  (9% attri) | 1 x 10^8^/d **drops** | 5 days  F/up: none | diarr **duration**: 70.7 + 26 hrs P<0.001 **Cure** by Day 3 69%* Less **LOS**: 4.3 + 1.3 d* No AE | control: 103.8 + 28.4 hr  Cured by Day 3: 12%  LOS: 5.5 + 1.8 d No AE | **Dinleyici** EC  2014 Acta Paediatrica |
| *-* | Mix of 2 strains: *L. helveticus* Rosell-52 + *L. rhamnosus* Rosell-11 “Lacidofil”   vs. placebo | kids 4-48 mon old with gastro-enteritis 3 sites DBPC **Canada** | n=132  n=123 done (7% attrition) | Low dose: 4 x 10^9^/d vs high dose 8 x 10^9^/d **sachets** | 5 days  F/up: none | missed >1 day daycare (37/61, 61% ns) **Duration** diarr: 71.1 + 78.3 hrs, p=0.39 NS | missed >1 day: 39/62, 63%)  **Duration** diarr: 63.5 + 64.3 hrs | **Freedman** SB 2015 Clinical Pediat |
| *+* | *S. boulardii* CNCM I-745 + ORT/IV “Reflor”  vs control (ORT/IV only)  open trial  RCT | 400 children in 8 hospitals (3-60 months old) 363 done (9% attrition)  TURKEY | n=363  all watery diarrhea  Inpatients (n=220), ER (n=51) or outpatient (n=92) | 500 mg/d  1 x 10^10^/d  **sachet** | 5 days  F/up:  none | (n=220) **Duration:** 75.4 + 33.1 h* P<0.001 [3.1 + 1.4 days] **LOS**: 4.6+ 1.7 days* P<0.001 | (n=143)Duration diarr:  99.8 + 32.5 hrs [4.2 + 1.3 dys] LOS:  6.1 + 1.7 No AE | **Dinleyici** EC  2015**A** Beneficial Microbes |
| *+* | *L. reuteri* DSM 17938  vs control, open   All had ORS *single blinded* | Outpatient kids (3-60 mon old) with acute infectious diarr **Turkey** | n=64, 60 done  (6% attrit) | 1 x 10^8^/d  **drops** | 5 days  F/up: none | (n=29) diarr **duration**: 2.5 + 1.0d p=0.01  No AEs | (n=31) diarr **dura**: 3.1 + 0.6 days | **Dinleyici** EC  2015**B** J Pediatrics |

**Treatment ped diarrhea-page 13**

|  | **Probiotic** | **pop** | **N** | **dose** | **duration** | **probiotic** | **controls** | **reference** |
| --- | --- | --- | --- | --- | --- | --- | --- | --- |
| *+* | *Bacillus clausii*  O/C, SIN/N/R, T strains) + ORT + Zinc  vs  ORT+Zinc control  RCT open label | 131 hospitalized children (6 mon-12 yrs old) with acute diarrhea  admitted to ped ward at one hospital  Mumbia India | 131 inpatient kids    enrolled at site #2: Dr DY Patil Hosp in Mumbia India | 2 x 10^9^/day  **spores in vials** | 5 days data only collected until Day 3 | (n=69)  duration diarr: **22.6** + nr hours, p<0.01  #bm/day=1 p<0.05  LOS=2.8 days p<0.05,  less Cost too | (n=62)  duration diarr: **47.05** + nr hours  # bm/day= 2.5/d   LOS=4.3 d | **Lahiri** K 2015**A** IOSR J Dental & Med Sciences  email from Keya Lahiri: 2015A and 2015B enrolled different periods from 2011-2014 |
| *+* | *Bacillus clausii (*O/C, SIN/N/R, T strains)+ ORT + Zinc vs  ORT+Zinc control  RCT open label | hospitalized children (6 mon-6 yrs) at one ped ward at a tertiary hospital with acute diarrhea  Mumbia India | 160   enrolled at site #2: Dr DY Patil Hosp in Mumbia India | 2 x 10^9^/day  **spores in vials** | 5 days data only collected until 72 hrs  F/up: none | (n=80) Duration diarrhea= **22.26** hrs no std dev data p<0.05  No data on AEs | (n=80) durat diarr= **34.16** hrs no std dev data | **Lahiri** K 2015**B** J Harmonized Research  email from Keya Lahiri: 2015A and 2015B enrolled different periods from 2011-2014 |

**Treatment ped diarrhea-page 14**
